# Reversible Dihydrogen Activation and Catalytic H/D Exchange with Group 10 Heterometallic Complexes**

**DOI:** 10.1002/anie.202213001

**Published:** 2022-12-07

**Authors:** Martí Garçon, Andreas Phanopoulos, Andrew J. P. White, Mark R. Crimmin

**Affiliations:** ^1^ Department of Chemistry Imperial College London Molecular Sciences Research Hub 82 Wood Lane London W12 0BZ UK

**Keywords:** H_2_ Activation, Heterometallics, Hydrides, Ligand-Assisted Oxidative Addition, Metalloligands

## Abstract

Reaction of a hexagonal planar palladium complex featuring a [PdMg_3_H_3_] core with H_2_ is reversible and leads to the formation of a new [PdMg_2_H_4_] tetrahydride species alongside an equivalent of a magnesium hydride co‐product [MgH]. While the reversibility of this process prevented isolation of [PdMg_2_H_4_], analogous [PtMg_2_H_4_] and [PtZn_2_H_4_] complexes could be isolated and characterised through independent syntheses. Computational analysis (DFT, AIM, NCIPlot) of the bonding in a series of heterometallic tetrahydride compounds (Ni–Pt; Mg and Zn) suggests that these complexes are best described as square planar with marginal metal‐metal interactions; the strength of which increases slightly as group 10 is descended and increases from Mg to Zn. DFT calculations support a mechanism for H_2_ activation involving a ligand‐assisted oxidative addition to Pd. These findings were exploited to develop a catalytic protocol for H/D exchange into magnesium hydride and zinc hydride bonds.

## Graphical abstract

Reaction of a hexagonal planar palladium complex having a [PdMg_3_H_3_] core with H_2_ is reversible and leads to the formation of a new [PdMg_2_H_4_] tetrahydride species. DFT calculations support a mechanism for H_2_ activation involving a ligand‐assisted oxidative addition to Pd. These findings were exploited to develop a catalytic protocol for H/D exchange into magnesium hydride and zinc hydride bonds.

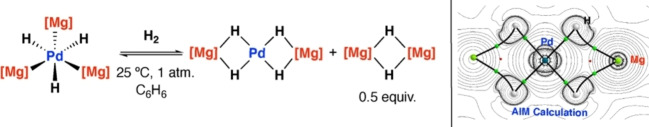

The reversible addition of H_2_ to metal sites is a fundamental step in catalysis. The importance of the reversibility of this step is perhaps most clearly illustrated in hydrogen‐for‐deuterium (H/D) exchange reactions. In its simplest form, H/D‐exchange involves the selective isotopic labelling of molecules through reaction with D_2_. Catalysts which promote this type of H/D‐exchange must be capable of reversible activation of H_2_ (and its isotopomers).

In recent years, there has been growing interest in the use of transition metal complexes bearing main group ligands for small molecule activation. Several systems incorporating B,[^1^, ^2^, ^3^, ^4^, ^5^, ^6^, ^7^, ^8^] Al‐In,[^9^, ^10^, ^11^, ^12^] Zn[^13^, ^14^, ^15^] and Sn^[16]^ ligands have been reported for H_2_ activation.^[17]^ A number of potential mechanisms for H_2_ activation, some of which involve cooperative behaviour of the main group ligand, have been discussed.^[18]^ Defined examples of reversible behaviour are more limited, the majority of these involve boron‐based ligands. For example, Sabo‐Etienne and co‐workers reported the reversible addition of H_2_ to a ruthenium complex supported by a borylene ligand.^[19]^ Peters and co‐workers documented reversible reactions of H_2_ to nickel‐borane, iron‐borane, and cobalt‐borane complexes.[^2^, ^4^, ^5^, ^6^] More recently, Okuda and co‐workers reported the reversible reaction of H_2_ with a Ga‐Zn compound, mediated by changes in solvent polarity.^[20]^ Reversible dihydrogen activation by these systems offers a potential approach to catalytic H/D‐exchange. However, reports of such reactivity are limited. Lu and co‐workers have demonstrated isotopic scrambling of H_2_+D_2_ mixtures to form HD using 1^st^ row transition metal complexes bearing Al, Ga or In based ligands.^[11]^

In this paper, we report a rare example of H_2_ activation using a transition metal complex supported by Mg‐based ligands. We show that this reaction is reversible and leads to a palladium tetrahydride complex, featuring an unusual structural motif. Based on DFT calculations, a mechanism for H_2_ activation involving a ligand‐assisted pathway is proposed. The understanding was used to develop a catalytic protocol for the selective isotopic labelling of molecular magnesium and zinc hydrides by H/D‐exchange using D_2_. During the preparation of this manuscript, Xu and co‐workers reported H_2_ activation by a Ni‐Mg heterometallic complex.^[21]^

We recently reported the hexagonal planar palladium complex **1**.^[22]^ Addition of 1 bar of H_2_ to a C_6_H_6_ solution of **1** at 25 °C resulted in clean formation of **Pd‐Mg** and 0.5 equiv of the molecular magnesium hydride **[2]_2_** (Scheme 1).

**Scheme 1:**
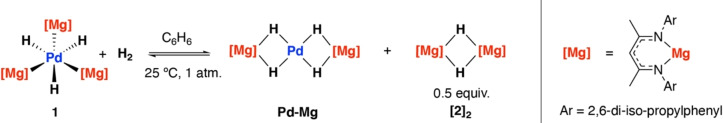
Reversible reaction of **1** with H_2_ to form **Pd‐Mg** and **[2]_2_**.

In C_6_H_6_ solution, **Pd‐Mg** demonstrated a highly symmetric ^1^H spectrum, featuring two equivalent Mg metalloligands and four hydride ligands, which presented a characteristic singlet resonance *δ*=−6.64 ppm at 298 K. VT NMR experiments in toluene‐d_8_ across a 213–298 K temperature range showed no evidence for decoalescence of this resonance.

H_2_ activation to **1** was found to be reversible. Removal of H_2_ from mixtures of **Pd‐Mg**+**[2]_2_** by freeze‐pump‐thaw cycles results in the regeneration of **1**. Similarly, the position of the equilibrium was found to be temperature dependent. A van't Hoff analysis between 213 and 298 K in toluene‐d_8_ provides thermodynamic parameters consistent with a reversible process, Δ*H*°=0.5 kcal mol^−1^, Δ*S*°=−3.6 cal K^−1^ mol^−1^, Δ*G*°_298K_=1.5 kcal mol^−1^. This measurement should be treated with caution as it is based on assumed dihydrogen concentrations and there are errors associated with quantification of species at low concentration by ^1^H NMR spectroscopy. Nonetheless, the thermodynamic parameters are consistent with the calculated Gibbs free energy value (Δ*G*°_298K_=2.7 kcal mol^−1^). Also, pressures ≥1 bar of H_2_ are necessary to drive the equilibrium towards **Pd‐Mg** (see Supporting Information), consistent with a marginally endergonic transformation. Due to its reversible formation, attempts to isolate **Pd‐Mg** were unsuccessful; **1** crystallises preferentially from solution. As such, we undertook alternative synthetic approaches to verify the structure of **Pd‐Mg**.

The reaction of [Pt(Me)_2_(κ^2^‐TMEDA)] with excess **[2]_2_** in C_6_H_6_ at 25 °C for 4 days, yielded **Pt‐Mg⋅TMEDA** as the major reaction product (Scheme 2).^[23]^ The ^195^Pt NMR spectrum shows a characteristic quintet resonance at *δ*=−5851 ppm (^1^ *J* ${}_{{}^{195}\mathrm{Pt}\text{-}{}^{1}H}$ =836 Hz) due to the coupling of the Pt centre to four equivalent hydride ligands. The corresponding hydride resonance appears at *δ*=−7.24 ppm in the ^1^H NMR spectrum. VT NMR data in toluene‐d_8_ between 243–298 K showed no evidence for decoalescence of this resonance. The data are again consistent with a highly symmetric structure, most likely due to TMEDA dissociation from **Pt‐Mg⋅TMEDA** in solution to form **Pt‐Mg**. DOSY studies on **Pt‐Mg⋅TMEDA** support the possibility that ligand dissociation from this complex is fast and reversible. Distinct diffusion coefficients for TMEDA (1.65×10^−9^ m s^−2^) and, what is proposed to be, **Pt‐Mg** (6.11×10^−10^ m s^−2^) are observed in C_6_H_6_ solution at 298 K. Below 243 K, the hydride resonance begins to broaden, possibly due to the slowing of this fluxional process. The presence of four hydride ligands rather than two in this complex arises from reaction of intermediate species with the solvent (C_6_H_6_). We have previously shown that related Mg‐ and Zn‐ reagents are capable of the C−H functionalisation of benzene with a Pd catalyst and isolated the resulting metal aryl complexes.[^24^, ^25^]

**Scheme 2:**
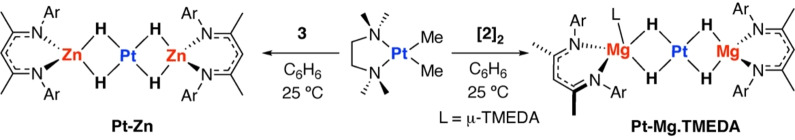
Synthesis of tetrahydride complexes **Pt‐Mg⋅TMEDA** and **Pt‐Zn**.

Reaction of [Pt(Me)_2_(κ^2^‐TMEDA)] with excess of the molecular zinc hydride [{(ArNCMe)_2_CH}Zn(H)] (Ar=2,6‐di‐iso‐propylphenyl, **3**) afforded colourless crystals of **Pt‐Zn** in 63 % yield (Scheme 2). A quintet resonance was observed in the ^195^Pt NMR spectrum at *δ*=−6079 ppm (^1^ *J* ${}_{{}^{195}\mathrm{Pt}\text{-}{}^{1}H}$ =772 Hz) and a diagnostic hydride resonance with Pt satellites in the ^1^H NMR spectrum at δ=−6.05 ppm. In this case, small amounts of [{(ArNCMe)_2_CH}ZnPh] (Ar=2,6‐di‐iso‐propylphenyl) were observed during the formation of **Pt‐Zn** confirming the role of the solvent as a source of hydride ligands. T_1_(min) relaxation times of **Pt‐Mg⋅TMEDA** and **Pt‐Zn** are long (0.5–0.6 s) and exclude the possibility of significant H−H interactions or dihydrogen character.[^26^, ^27^] A closely related species **Ni‐Mg** has very recently been reported by Xu and co‐workers.^[21]^

Both **Pt‐Mg⋅TMEDA** and **Pt‐Zn** could be isolated as crystalline solids. X‐ray diffraction studies confirmed that the coordination geometry at the transition metal is square planar (Figure 1).^[28]^ In the solid state, the four hydride ligands of **Pt‐Zn** are organised in a near symmetric configuration around the transition metal and sit in the same plane as both the Pt and Zn atoms. The hydride atoms were located within the Fourier difference map and their positions confirmed by DFT calculations. The geometry at Zn is near tetrahedral and the β‐diketiminate chelate twisted and orthogonal to the plane created by the hydrides. The Pt‐Zn distance in **Pt‐Zn** is 2.4466(4) Å. Despite being close to the sum of the covalent radii (Pauling, 2.53 Å; Pyykkö, 2.38 Å), computational analysis of the bonding suggests weak metal‐metal interactions (see below), which supports assignment of the geometry at Pt as 4‐coordinate square planar. The average H‐Pt‐H angle across all unique solid‐state structures for **Pt‐Mg⋅TMEDA** and **Pt‐Zn** is 89.9(3)° (74.2(3)–105.7(3)°). This tetrahydride core is reminiscent of a Ru‐Zn_2_ tetrahydride complex reported by Fischer and co‐workers.^[29]^

**Figure 1:**
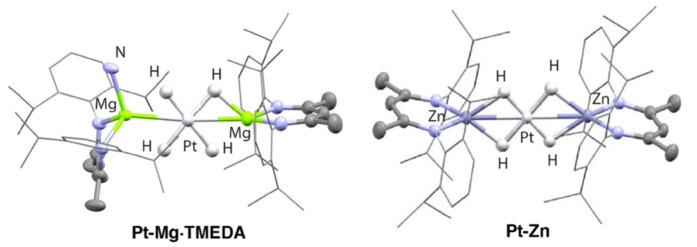
Molecular structures of **Pt‐Mg⋅TMEDA** (truncated for clarity) and **Pt‐Zn** from single crystal X‐ray diffraction studies.

**Pt‐Mg⋅TMEDA** appears as a dimer in the solid state due to the ability of TMEDA to act as a bridging ligand. As a result, there are some differences in the heterometallic core of **Pt‐Mg⋅TMEDA** compared to **Pt‐Zn**. Increasing the coordination at one magnesium site results in a distortion of the position of the β‐diketiminate ligand and induces an asymmetry in the Pt‐Mg distances. These take values of 2.5605(15) and 2.7124(15) Å to the 4‐ and 5‐coordinate magnesium centres respectively, with the longer distance consistent with the higher coordination number at Mg. Ternary hydrides of s‐block and group 10 metals have been reported and characterised by neutron diffraction,[^30^, ^31^] and one homometallic complex featuring a [Ni_3_H_4_]^2−^ core has been reported.^[32]^

The bonding in a series of heterometallic tetrahydride complexes (M^1^=Ni–Pt; M^2^=Mg or Zn) was investigated by computational methods. Superficially this structural type resembles that of **1**, in that it contains six co‐planar atoms closely arranged around a palladium centre. However, the electronic structure of these species is different. The analysis supports only a very small component to the metal–metal bonding in these tetrahydride complexes relative to the metal‐hydride interactions. In general, while weak the significance of the metal‐metal interaction marginally increases for Pt>Pd>Ni and Zn>Mg.

NBO calculations are consistent with a large ionic contribution to the bonding based on the large amount of charge separation with positive charges on the main group atoms (>1.5) and negative charges on the hydrides (<−0.3). To a first approximation these species can be formalised as containing [NiH_4_]^4−^, [PdH_4_]^4−^ or [PtH_4_]^4−^ fragments interacting with two cationic Mg or Zn moieties. The covalent character is higher for the zinc analogues compared with their magnesium counterparts. Interaction between the two fragments occurs almost exclusively by bridging hydride interactions. This conclusion is supported by comparison of the Wiberg Bond Indicies (WBIs). For example, in **Pt‐Mg** the WBI follow the trend Pt‐H≫Mg‐H>Pt‐Mg. The Pt‐Mg WBI of 0.07 is low. The same trend is observed in **Pt‐Zn** but with larger absolute values of the WBIs for the Pt‐Zn and Zn‐H bonds (Table 1). The conclusions are supported by QTAIM data which for **Pt‐Mg** returns bond paths and associated bond critical points consistent with a bonding interaction dominated by metal hydride interactions. As with the NBO data, QTAIM calculations on **Pt‐Zn** suggest the metal‐metal interaction is slightly more significant than **Pt‐Mg**, as bond paths are present between the metals (Figure 2a). This bonding picture is further supported by NCIplot data which show attractive interactions between the transition and main group metals in **Pt‐Zn** (Supporting Information, Figure S14). In combination, the analysis suggests that the Zn‐H bonds are partially activated in **Pt‐Zn**. A related bonding situation has been described for a σ‐dihydrideborate complex of osmium.^[33]^

**Table 1:** Average NPA charges and Wiberg Bond Indices.

	**Ni‐Mg**	**Pd‐Mg**	**Pt‐Mg** ^[a]^	**Ni‐Zn**	**Pd‐Zn**	**Pt‐Zn**
*NPA Charges* ^[b]^						
M^1^=Ni, Pd, Pt	0.19	0.03	−0.10	0.15	0.03	−0.10
M^2^=Mg, Zn	1.69	1.68	1.70	1.53	1.53	1.54
H	−0.45	−0.41	−0.38	−0.39	−0.35	−0.33
*Wiberg Bond Indices* ^[b]^						
M^1^−M^2^	0.05	0.06	0.07	0.07	0.08	0.09
M^1^−H	0.42	0.39	0.43	0.39	0.37	0.41
M^2^−H	0.13	0.14	0.12	0.19	0.20	0.17

[a] **Pt‐Mg** not **Pt‐Mg⋅TMEDA**. [b] NBO 6.0. ωB97X/Def2‐TZVPP (Mg, Zn, C, N, H)/SDD (Ni, Pd, Pt).

**Figure 2:**
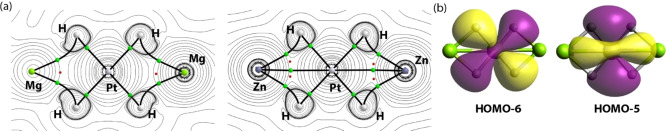
a) QTAIM molecular graphs for **Pt‐Mg** and **Pt‐Zn**. Showing key bond critical points (BCPs, green spheres) and ring critical points (RCPs, red spheres). b) Selected bonding molecular orbitals of [PtH_4_Mg_2_]^2+^.

A MO diagram for [Pt(μ‐H_4_)Mg_2_]^2+^, a simplified model of **Pt‐Mg** with *D*_2h_ symmetry, was derived (Supporting Information). The key bonding orbital interactions involve orbitals delocalised across Pt‐H and Mg‐H units. For example, the HOMO‐6 (50 % Pt *d*, 44 % H *s* AOs) and HOMO‐5 (90 % Pt *d* AOs) are heavily Pt‐H based (Figure 2b). There are practically no MOs showing significant metal–metal interactions; aside from HOMO‐5, which presents little overlap (mainly Pt based). These findings are completely consistent with the NBO and QTAIM calculations and suggest that the metal–metal interactions in these compounds are weak at best and less important than metal‐hydrogen interactions.

To gain further insight into the mechanism of H_2_ activation by **1** a series of pathways were calculated by DFT.^[34]^ Both associative and dissociative mechanisms were considered. The HOMO of **1** is mainly composed of the Pd $d_{z^{2}}$ orbital, lying perpendicular to the tetrahydride plane.^[22]^ Although this has the appropriate orientation and symmetry to interact with H_2_ in an associative step, calculations did not lead to identification of clear minima for the hydrogen adduct of **1** (**1‐H_2_**) on the potential energy surface. In contrast, a dissociative mechanism for H_2_ splitting was calculated to be a facile and reversible process (Figure 3). Hence, dissociation of an equiv. of **2** from **1** is calculated to form **Int‐1** with a small energy cost (Δ*G*°_298K_=10.3 kcal mol^−1^). This process occurs with a shift in the bonding of **1** along the continuum from hexagonal planar toward trigonal planar,^[35]^ strengthening the Mg‐H interactions in the equatorial plane, and pre‐organising the complex for dissociation of an equivalent of **2**. Similar coordinatively unsaturated intermediates were recently described in related systems for the zincation of C−H bonds.^[24]^ Association of H_2_ generates the dihydrogen complex **Int‐2** with subsequent H_2_ activation occurring by a ligand‐assisted oxidative addition through **TS‐1** (Δ*G* ^≠^_298K_=13.6 kcal mol^−1^) leading to the intermediate **Int‐3**. A subsequent rotation through **TS‐2** (Δ*G* ^≠^_298K_=15.7 kcal mol^−1^) forms the experimentally observed tetrahydride product **Pd‐Mg**. The overall reaction is calculated to occur with Δ*G*°_298K_=2.7 kcal mol^−1^. Hence the calculations provide reasonable model for a reversible process that occurs at 25 °C through addition and removal of H_2_.

**Figure 3:**
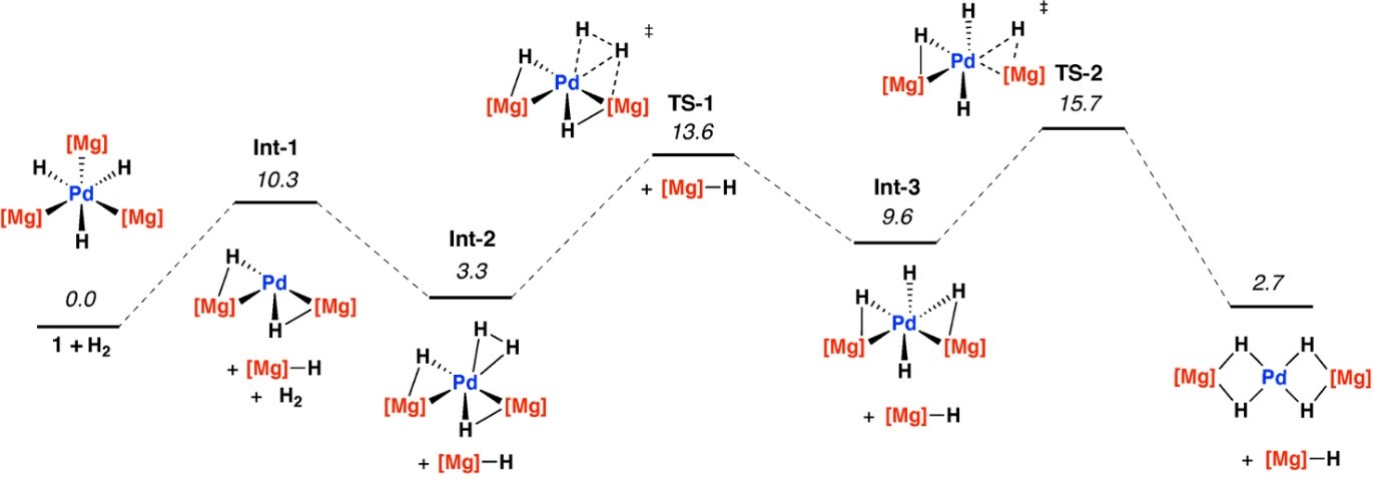
Calculated pathway for H_2_ activation by a dissociative mechanism from **1**.

The calculated mechanism bears some resemblance to a σ‐complex assisted metathesis pathway.[^36^, ^37^] H_2_ addition to a Ni complex related to **1** has been calculated to occur by a σ‐bond metathesis pathway.^[21]^ Despite these precedents, NBO analysis of key intermediates is consistent with the assignment as a ligand‐assisted oxidative addition. Throughout the pathway the *NPA* charge on Pd becomes more positive (**Int‐1**=−0.37; **Int‐2**=−0.17; **TS‐1**=−0.16; **Int‐3**=−0.07; **TS‐2**=−0.05) suggestive of an increase in formal oxidation state of the transition metal on addition of H_2_. The WBI of the breaking H^1^‐H^2^ bond is 0.82 in **Int‐2** and 0.45 in **TS‐1**. The involvement of both the Pd and a Mg centre in this transition state is supported by NBO calculations which show a charge asymmetry across the dihydrogen unit (H^1^=−0.21; H^2^=0.12) along with a small but significant H^1^‐Mg interaction characterised by a WBI of 0.09. Ligand‐assisted oxidative addition is increasingly recognised as a mechanism of importance for heterometallic complexes. Previous calculations have suggested this mechanism may be in operation for addition of C‐F to Pd‐Mg complexes^[38]^ and H_2_ addition to Ru‐Zn complexes.^[13]^

The proposed mechanism of H_2_ activation by **1** involves both breakage and formation of H−H and Mg−H bonds in the equatorial plane and strongly suggests that the activation of both these types of bonds is possible at the same transition metal centre. This idea was further exploited through the development of a catalytic protocol for H/D‐exchange (Scheme 3).

**Scheme 3:**
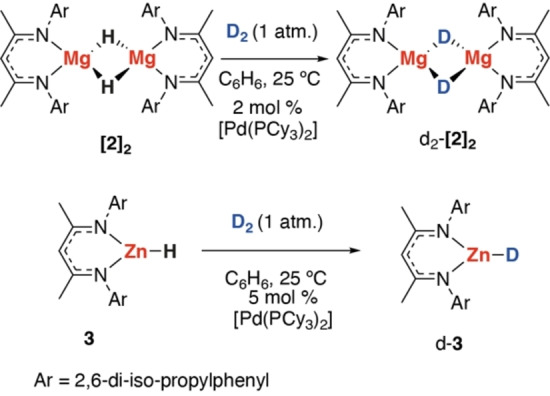
Palladium catalysed H/D‐exchange between **[2]_2_** and **3**.

The reaction of **[2]_2_** with 1 bar D_2_ in C_6_D_6_ in the presence of 2 mol % of [Pd(PCy_3_)_2_] led to partial deuteration of the Mg−H bond. The reaction occurred rapidly at 25 °C, and heating the mixture did not result in further D‐incorporation. Formation of H_2_ (*δ*=4.46 ppm) and HD (*δ*=4.42 ppm, ^1^ *J*_H‐D_=43 Hz) was observed by ^1^H NMR spectroscopy. A low‐intensity resonance at *δ*=−6.64 ppm belonging to complex **Pd‐Mg** was also observed, suggesting that this complex may play a role in the catalytic reaction. Evacuation of the atmosphere and recharging with fresh D_2_ gas resulted in further D‐incorporation. After three recharges, the Mg−H bond had been fully deuterated (99 % D‐incorporation). Evaporation of the volatiles and washing the crude with n‐hexane allowed the isolation of d^2^‐**[2]_2_** in excellent purity and yield (90 %). Similarly, the reaction of **3** with 1 bar D_2_ catalysed by 2–5 mol % [Pd(PCy_3_)_2_] led to exclusive formation of d‐**3** in 90 % yield under the same conditions (99 % D‐incorporation). While routes to synthesise isotopically labelled Mg and Zn hydrides are known, this new approach is operationally simple and does not rely on (expensive) specialised isotope sources such as deuterated silanes or boranes.

In summary, we report a rare example of reversible H_2_ activation at a palladium complex bearing Mg‐based metalloligands. The product of H_2_ addition is an unusual tetrahydride complex of palladium containing a [PdH_4_Mg_2_] core, and analogous [PtH_4_Mg_2_] and [PtH_4_Zn_2_] containing species were isolated by independent synthesis. DFT calculations support a dissociative mechanism in which, following generation of a coordinatively unsaturated intermediate, H_2_ splitting occurs by ligand‐assisted oxidative addition. These findings were used to develop a simple approach to catalytic H/D‐exchange into main group hydrides using D_2_; a reaction that may have wide synthetic applications given the utility of these reagents and scarcity of suitable methods (and deuterium sources) for preparation of isotopically labelled analogues.

## Conflict of interest

The authors declare no conflict of interest.

## Supporting information

As a service to our authors and readers, this journal provides supporting information supplied by the authors. Such materials are peer reviewed and may be re‐organized for online delivery, but are not copy‐edited or typeset. Technical support issues arising from supporting information (other than missing files) should be addressed to the authors.

Supporting Information

Supporting Information

Supporting Information

## Data Availability

The data that support the findings of this study are available in the Supporting Information of this article.
