# Supplementary material for: Reversible Dihydrogen Activation and Catalytic H/D Exchange with Group 10 Heterometallic Complexes
Source: Angew Chem Int Ed Engl. 2022 Dec 7;62(2):e202213001. doi: 10.1002/anie.202213001 (PMC10107683; doi:10.1002/anie.202213001)
Supplement: Supplementary file 1 — Supporting Information [file ANIE-62-0-s003.pdf]

Angewandte Chemie
Eine Zeitschrift der Gesellschaft Deutscher Chemiker

Supporting Information

Reversible Dihydrogen Activation and Catalytic H/D Exchange with Group 10 Heterometallic Complexes

*M. Garçon, A. Phanopoulos, A. J. P. White, M. R. Crimmin**

Supporting Information

Table of Contents

1.	General Experimental	S2
2.1	Hydrogenation of **1**	S3
2.2	Synthesis of **Pt-Mg** an **Pt-Zn**	S8
2.3	Catalytic H/D exchange of magnesium and zinc hydrides	S13
3.	X-ray Crystallographic Data	S15
4.1	Computational Methods	S18
4.2	NBO Calculations	S19
4.3	AIM Calculations	S22
4.4	NCIPlot Calculations	S24
4.4	Molecular Orbital Analysis	S25
4.5	Mechanism of H_2 Activation	S26
4.7	XYZ Coordinates	S30
5.	References	S51

1. General Experimental

Unless otherwise specified, all manipulations were carried out using standard Schlenk and glovebox techniques, under inert atmosphere (nitrogen or argon). A MBRAUN Labmaster glovebox was employed operating with concentrations of H_2O and O_2 below 0.1 ppm. Anhydrous solvents were obtained from a Grubbs type SPS system and stored over activated 3Å molecular sieves under inert atmosphere. Alternatively, they were dried using molecular sieves and degassed by freeze-pump-thaw procedures. Stable liquid organic reagents were dried over 3Å molecular sieves and degassed by freeze-pump-thaw cycles before use. All other reagents were obtained from commercial suppliers (Sigma-Aldrich, Alfa Aesar, Fluorochem) and used without further purification. The synthesis and characterisation of the β-diketiminate ligand[1] and compounds **1**,[2] **[2]$_2$**,[3] **3**[4] have been described elsewhere. [PtMe$_2$(κ^2-TMEDA)] was synthesised from [PtCl$_2$(1,5-COD)] as described below.[5]

^{1}H, ^{2}H, ^{13}C{^{1}H}, and ^{195}Pt NMR spectra and two-dimensional experiments (*e.g.* COSY, NOESY, DOSY, HSQC, HMBC, ^{1}H-^{195}Pt-HMQC) were conducted in J. Young's NMR tubes on BRUKER 400 MHz or 500 MHz spectrometers. Chemical shifts (δ) were referenced to internal solvent resonances. Data was processed using the MestreNova or TopSpin software. The coupling constants (*J*) are reported in Hertz (Hz). The following abbreviations are used to define multiplicities: s (singlet), d (doublet), quint. (quintet), sept. (septet), m (multiplet), br s (broad signal).

Single crystal X-ray data was collected using an Agilent Xcalibur PX Ultra A diffractometer, and the structures were refined using the SHELXTL and SHELX-2013 program systems.[6]

2.1 Hydrogenation of 1

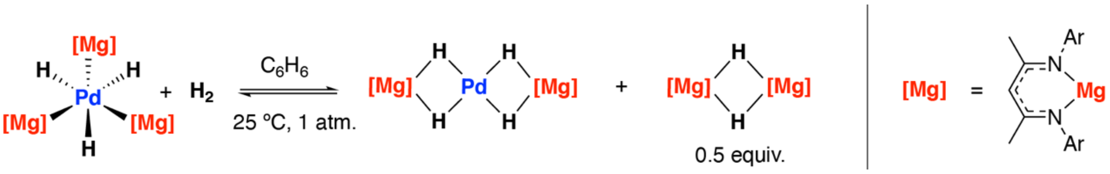

In a J. Young's NMR tube, **1** (7 mg, 0.005 mmol, 1 equiv.) was dissolved in dry C_6H_6 (0.6 mL). The atmosphere in the headspace was evacuated and the tube charged with H_2 gas (1 bar). Clean formation of **[2]₂** (0.5 equiv.) and **Pd-Mg** was observed by ^{1}H NMR spectroscopy (quantitative yield, as measured against a ferrocene standard in C_6D_6). Removal of the volatiles *in vacuo* resulted in partial reformation of **1**. Our attempts to crystallise the product under H_2 atmosphere failed. Nonetheless, the spectroscopic signals for **Pd-Mg** could be unambiguously assigned.

^{1}H NMR (400 MHz, C_6H_6) δ (ppm): –6.64 (s, 4H, Pd*H*₄), 1.11 (d, $^3J_{H-H}$ = 6.5 Hz, 24H, CH*Me*₂), 1.12 (d, $^3J_{H-H}$ = 6.4 Hz, 24H, CH*Me*₂), 1.64 (s, 12H, *Me*), 3.13 (sept., $^3J_{H-H}$ = 6.9 Hz, 8H, C*H*Me₂), 4.91 (s, 2H, β-C*H*), aromatic region (*ca*. 7.00–7.15) obscured by solvent.

***T₁*(min) relaxation time** (Pd*H*₄ signal, 298 K): 0.5 s.

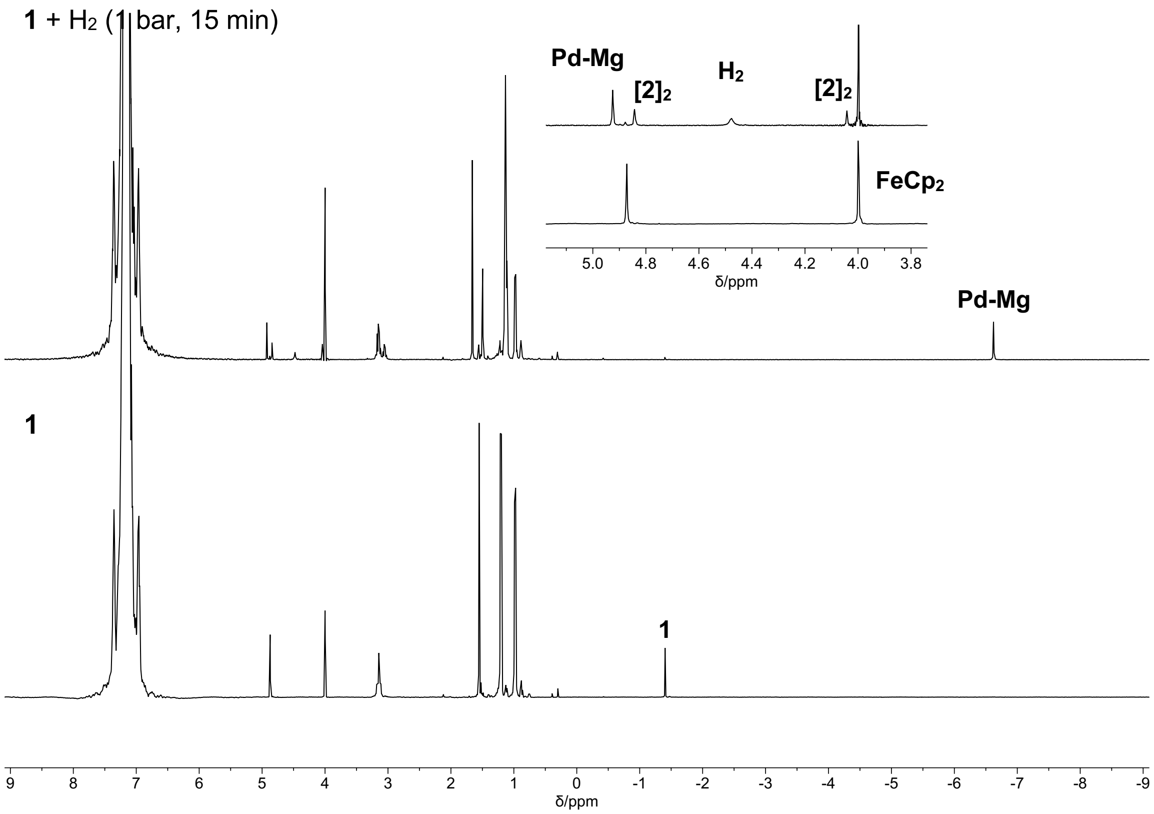

Figure S1. *Stacked ^{1}H NMR spectra showing reaction between* ***1*** *and H_2.*

Variable temperature ^{1}H NMR spectra were recorded for the hydrogenation of **1** (5 mg, 0.003 mmol) using both 100% H_2 and 95:5 N_2/H_2, both at a total pressure of 1 bar between 213–298 K. 1,2,4,5-tetramethylbenzene (6 mg, 0.0447 mmol) was used as an internal standard and the total volume was 0.6 mL. Toluene was used as the solvent with a sealed toluene-d_8 lock tube to lock and shim the spectrometer. With 100% H_2 at 1 bar (p_{H2} = 1 bar) the equilibrium is shifted almost completely to **Pd-Mg**, while with 95:5 N_2/H_2 at 1 bar (p_{H2} = 0.05 bar), a ratio of 3.4:1 **1**:**Pd-Mg** is observed at 298 K. The ratio of **1**:**Pd-Mg** at the lower H_2 pressure remains constant over the entire temperature range and no dissolved H_2 is observed in the spectra (δ_H: 4.55 ppm), suggesting insufficient H_2 is dissolved to observe a shift in equilibrium (Figure S2).

Despite, the low concentration of **1** under 1 bar H_2, small variations in the concentrations of **1** and **Pd-Mg** over the temperature range 213–283 K allowed the equilibrium to be probed *via* a van't Hoff plot (Figures S3 and S4) with the following caveats/assumptions which cause errors and deviations from linearity:

- The absolute amount of **1** in solution remains low over the temperature range, resulting in errors associated with accurately determining [**1**].
- The total concentration of [**1** + **Pd-Mg**] remained constant over the course of the experiment despite decomposition being observed after the measurements were complete.
- The low solubility of $[\mathbf{2}]_2$ means accurate determination of its concentration was not possible. To maintain stoichiometry, the assumption that [**2**] = [**Pd-Mg**] was used.
- The concentration of dissolved H_2 at any given temperature is difficult to determine.[7] This is a result of line broadening of the signal due to exchange with **Pd-Mg**, as well as the change in pressure and solubility at various temperatures. The $[H_2]$ at each temperature was estimated using the same assumptions as have been previously described. The data was modelled using empirically determined temperature dependant solubility molar ratios (χ_{H2}).[8] This is used in preference to the standard state of gas which states $[H_2]$ = 1 M at 298 K. Empirically, 1 atm H_2 in toluene at 298 K gives $[H_2] \approx 0.003$ M, orders of magnitude lower than 1 M, and hence more realistic and closer to actual experimental conditions.

Consequently, van't Hoff analysis gives the following approximate thermodynamic parameters for the equilibrium between **1** and **Pd-Mg**: $\Delta H = +0.5$ kcal mol^{-1}, $\Delta S = -3.6$ cal mol^{-1} and $\Delta G_{298K} = +1.5$ kcal mol^{-1}. These are broadly in agreement with observations of an equilibrium as well as with the calculated thermodynamics.

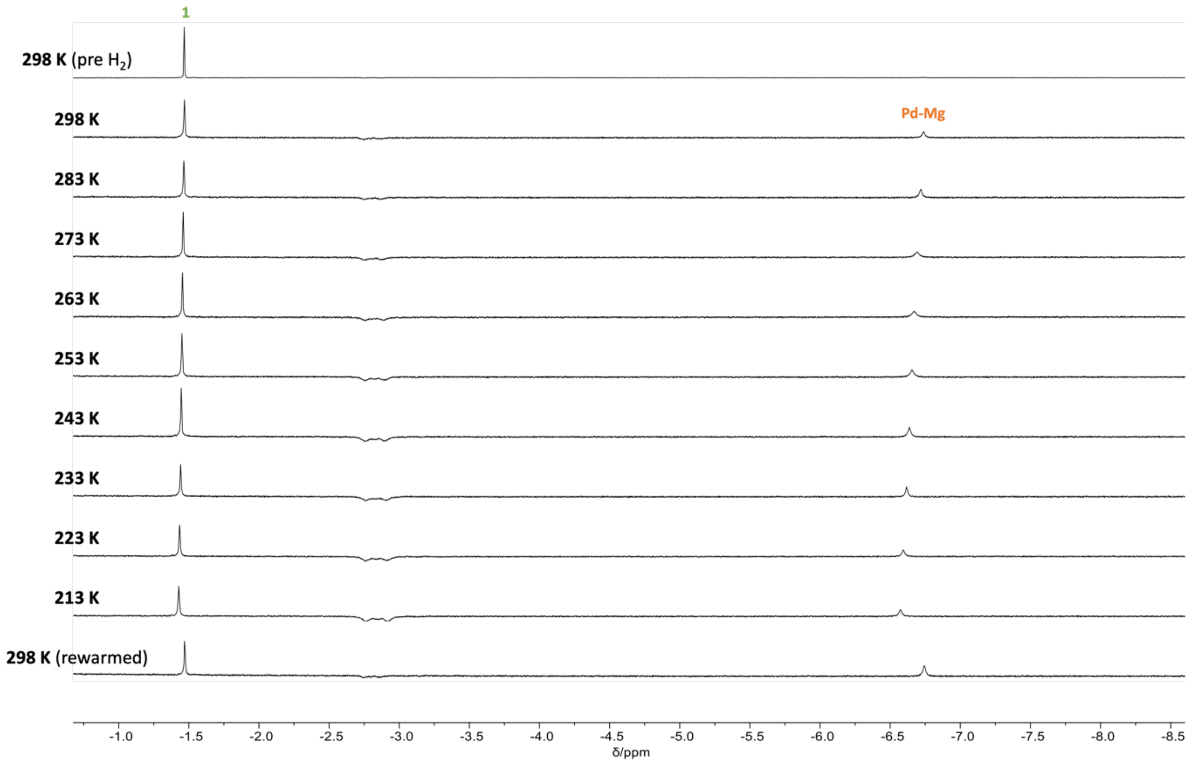

Figure S2. *Stacked variable temperature* [1]*H NMR spectra (213–298 K) showing reaction between* ***1*** *and* H_2 *(0.05 bar)*

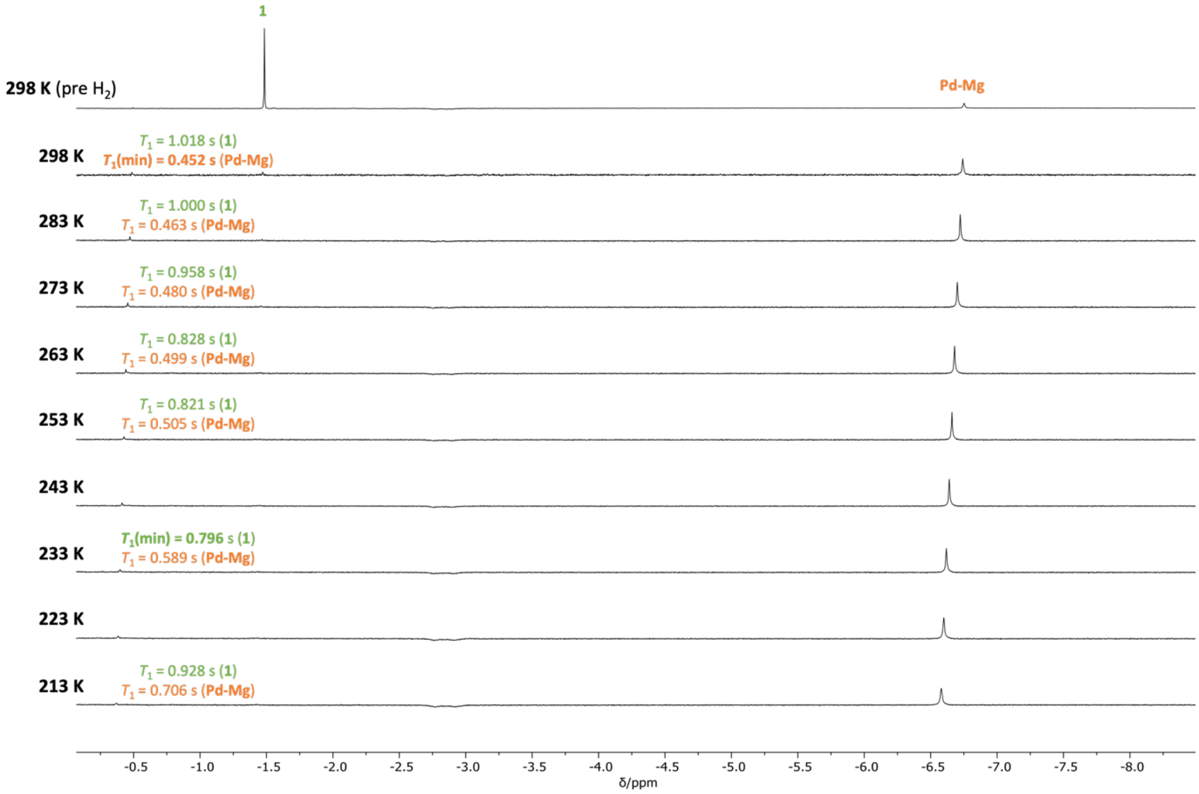

Figure S3. *Stacked variable temperature* [1]*H NMR spectra (213–298 K) showing reaction between* ***1*** *and* H_2 *(1 bar)*

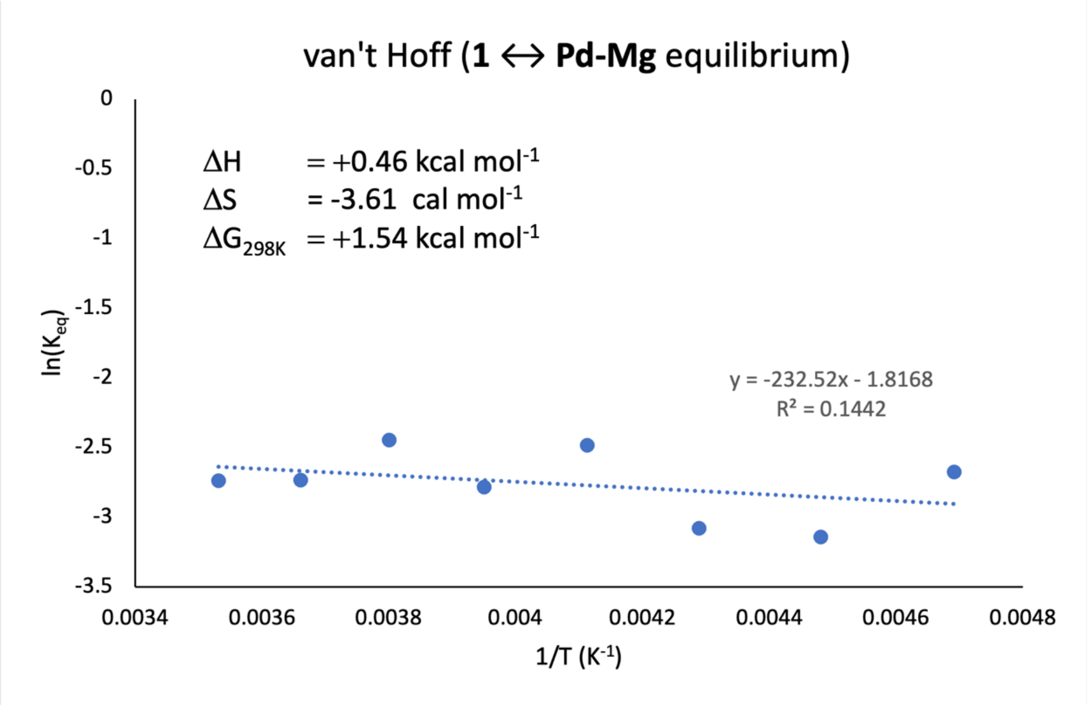

Figure S4. *Van't Hoff analysis for equilibrium between* ***1*** *+ H_2 and* ***Pd-Mg*** *+ [**2**]$_2$ under 1 bar H_2 between 213–283 K*

While variable temperature NMR spectra were being recorded, T_1 relaxation times were also recorded (Figure S3). Values for both **1** and **Pd-Mg** were recorded to determine T_1(min). **1** showed a T_1(min) of 0.796 s at 233 K while **Pd-Mg** showed a T_1(min) of 0.452 s at 298 K (Figure S5). The lower overall T_1(min) for **Pd-Mg** compared to **1** is likely a result of fast exchange with H_2, artificially lowering its value. Nevertheless, these values are still large enough to rule out the presence of any coordinated dihydrogen character in **Pd-Mg**.

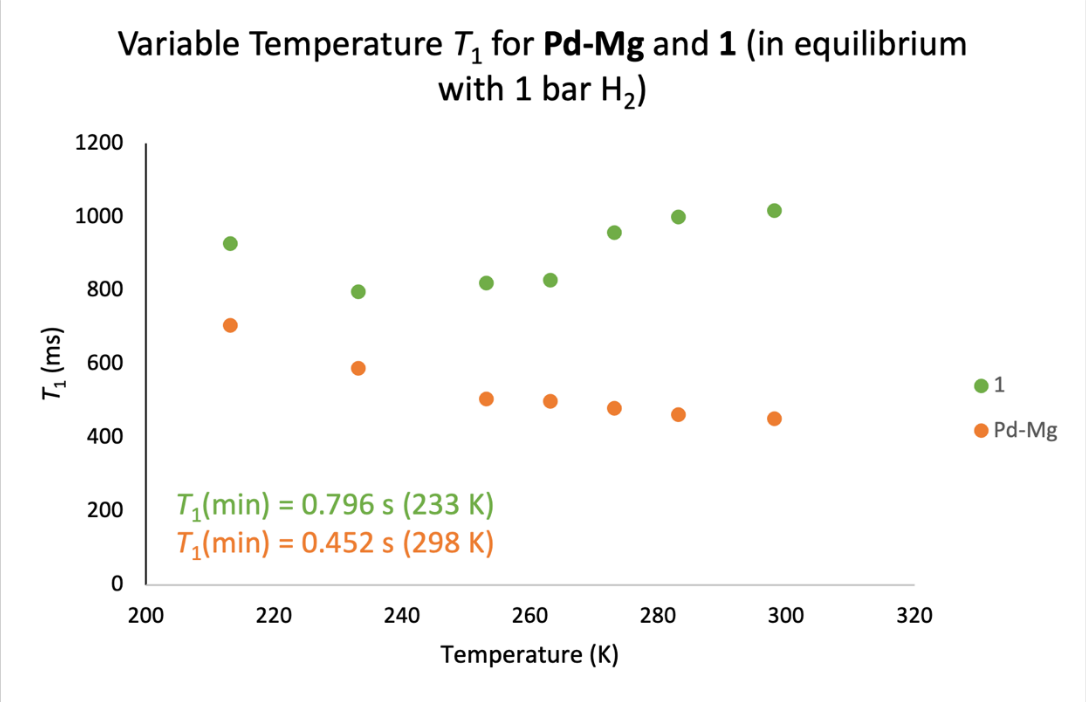

Figure S5. *Variable temperature T_1 relaxation times for **1** and **Pd-Mg** over 213–298 K*

2.2 Synthesis of Pt-Mg and Pt-Zn

Synthesis of [Pt(Me)$_2$(κ^2-TMEDA)]: [PtMe$_2$(1,5-COD)] (600 mg, 1.8 mmol, 1 equiv.) was dissolved in neat TMEDA (5 mL) in a Schlenk flask and the mixture heated at 100°C for 1h. The volatiles were then removed *in vacuo* and the solid washed with dry Et$_2$O (2 x 6 mL). The solid was dried under vacuum and taken into the glovebox. The solid was dissolved in C$_6$H$_6$, filtered through a glass fibre and the solvent evaporated. The desired product was isolated as a colourless crystalline solid (310 mg, 0.91 mmol, 50% yield). **^{1}H-NMR (400 MHz, C$_6$D$_6$) δ (ppm):** 1.09 (s, $^2J_{H\text{-}Pt}$ = 89 Hz – satellites, 6H), 1.64 (s, 4H), 2.19 (s, $^3J_{H\text{-}Pt}$ = 21 Hz – satellites, 12H). **^{13}C-{^{1}H}-NMR (100 MHz, C$_6$D$_6$) δ (ppm):** -22.3 (s, $^1J_{C\text{-}Pt}$ = 863 Hz – satellites, 2xCH$_3$), 47.9 (4xCH$_3$), 61.2 (2xCH$_2$).

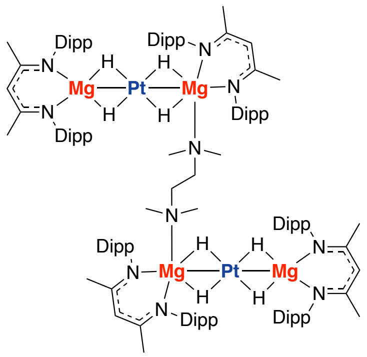

Synthesis of Pt-Mg·TMEDA: In a J. Young's NMR tube, **[2]$_2$** (78 mg, 0.088 mmol, 2.0 equiv.) and [PtMe$_2$(κ^2-TMEDA)] (15 mg, 0.044 mmol, 1.0 equiv.) were dissolved in benzene (1 mL). The resulting solution was left at 25 °C for 4 days and then heated to 40 °C for 1 day. The precipitate was filtered off through a glass fibre and the solvent and volatiles were removed *in vacuo*. The crude product was treated with a small amount of *n*-hexane (0.5 mL), the solution filtered through a glass fibre and the clear solution was stored at –35 °C to afford colourless crystals of the desired product **Pt-Mg·TMEDA** (42 mg, 0.018 mmol, 42% yield).

^{1}H NMR (500 MHz, C$_6$D$_6$) δ (ppm): –7.24 (s, $^1J_{H–Pt}$ = 836 Hz (satellites), 8H, Pt*H*$_4$), 1.07 (d, $^3J_{H–H}$ = 6.8 Hz, 48H, CH*Me*$_2$), 1.08 (d, $^3J_{H–H}$ = 6.8 Hz, 48H, CH*Me*$_2$), 1.59 (s, 24H, *Me*), 2.02 (br s, 12H, N*Me*$_2$), 2.27 (br s, 4H, C*H*$_2$), 3.08 (sept., $^3J_{H–H}$ = 6.9 Hz, 16H, C*H*Me$_2$), 4.85 (s, 4H, β-C*H*), 6.96–7.07 (m, 24H, *Ar*).

***T_1* relaxation time** (Pt*H*$_4$ signal, 298 K): 0.6 s.

***T_1*(min) relaxation time** (Pt*H*$_4$ signal, 273 K): 0.5 s.

DOSY: TMEDA resonances diffuse (D_{ave} = 1.65 x 10^{-9} m^2/s) separately to β-diketiminate unit (D_{ave} = 6.11 x 10^{-10} m^2/s).

^{13}C{^{1}H} NMR (126 MHz, C$_6$D$_6$) δ (ppm): 23.7 (8xCH$_3$), 24.1 (16xCH$_3$), 25.1 (16xCH$_3$), 28.5 (16xCH), 45.8 (4xCH$_3$), 57.9 (2xCH$_2$), 95.5 (4xCH), 123.9 (16xCH), 125.7 (8xCH), 142.1 (16xC), 143.1 (8xC), 169.6 (8xC).

^{195}Pt NMR (108 Hz, C$_6$H$_6$) δ (ppm): –5851.3 (quint., $^1J_{H–Pt}$ = 836 Hz).

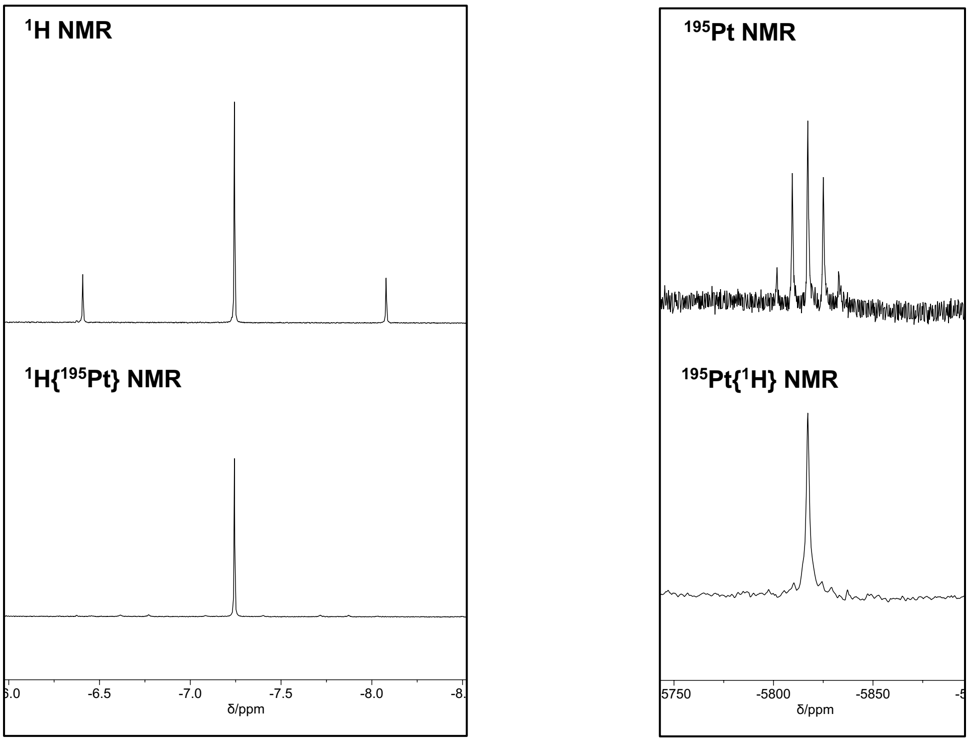

Figure S6. *Stacked ^{1}H (left) and ^{195}Pt (right) NMR spectra showing the coupled (top) and decoupled (bottom) spectra of **Pd-Mg·TMEDA.***

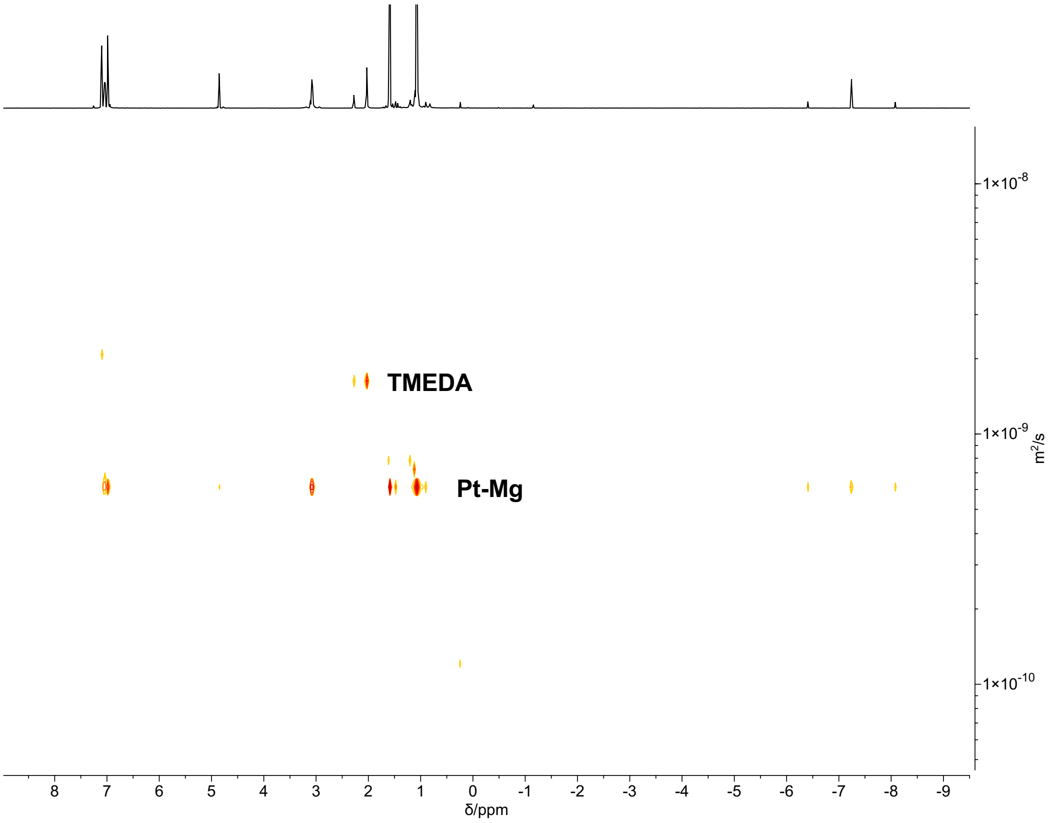

Figure S7. *^{1}H DOSY NMR spectrum of **Pd-Mg·TMEDA** showing different diffusion coefficients for TMEDA and ligand fragments.*

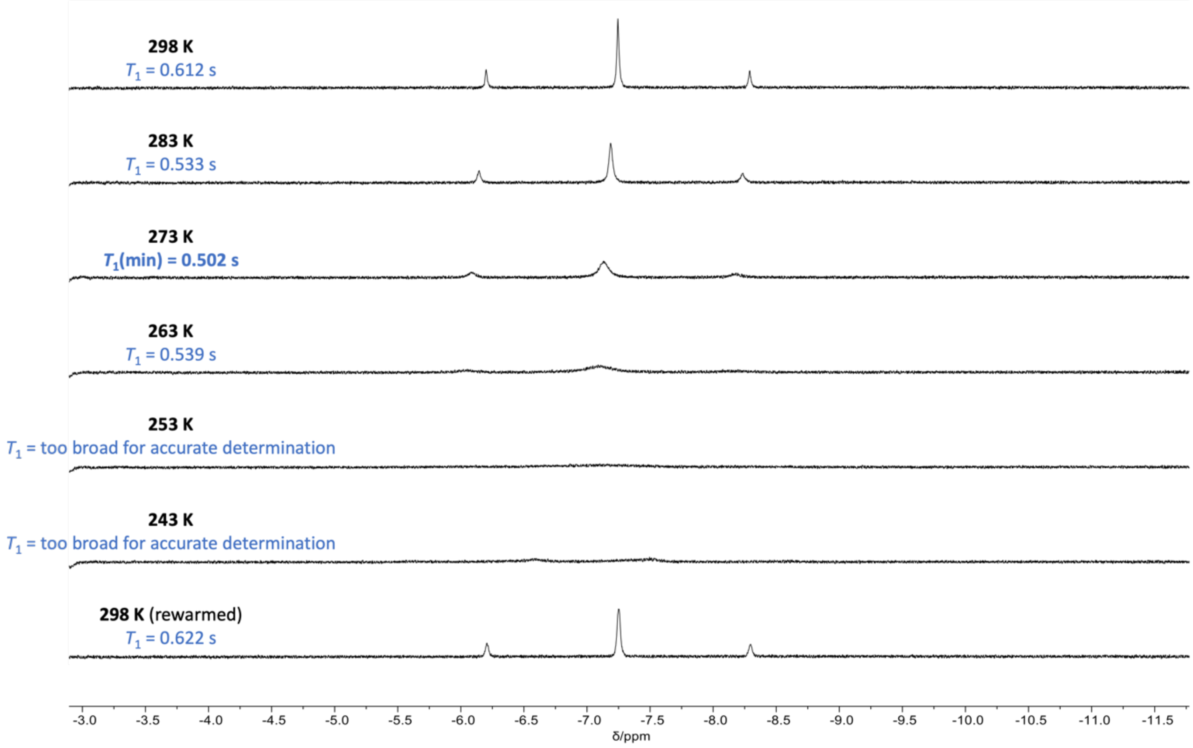

Figure S8. *Stacked variable temperature ^{1}H NMR spectra (243–298 K) for* ***Pt-Mg***

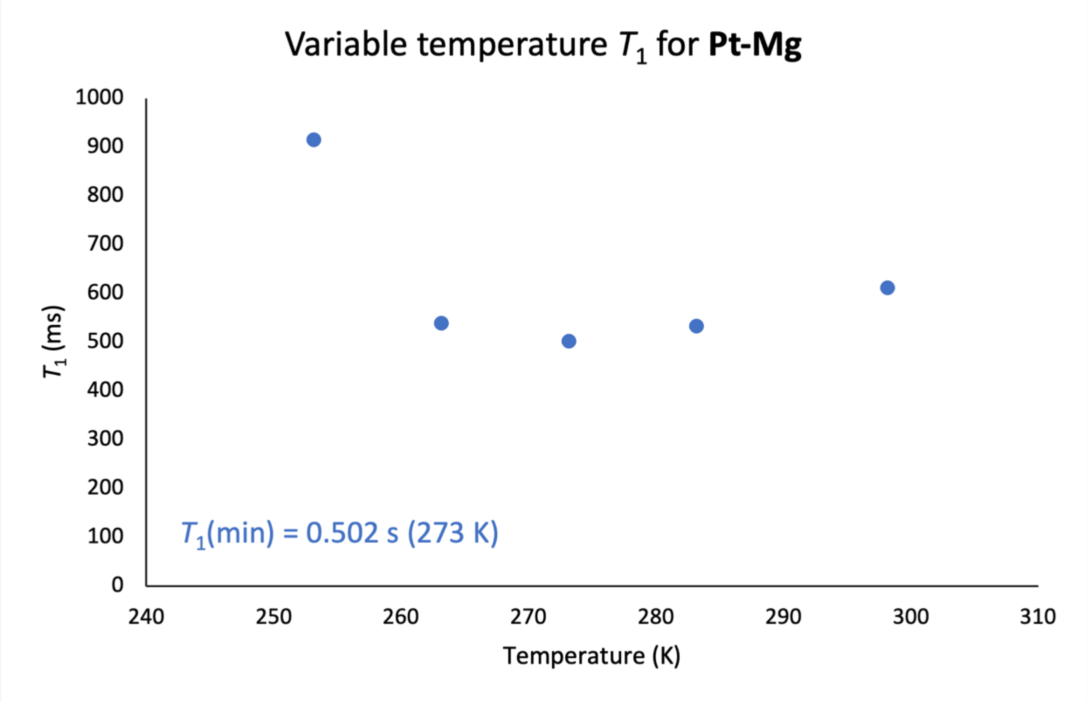

Figure S9. *Variable temperature T_1 relaxation times for* ***Pt-Mg*** *over 243–298 K*

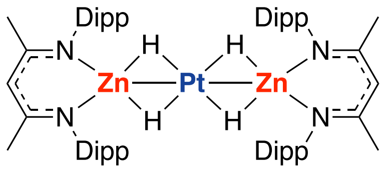

Synthesis of Pt-Zn: In a J. Young's NMR tube, **3** (39.7 mg, 0.082 mmol, 4.0 equiv.) and [$PtMe_2$(κ^2-TMEDA)] (7 mg, 0.021 mmol, 1.0 equiv.) were dissolved in benzene (0.7 mL). The resulting solution was left at 25 °C for 4 days. The solvent and volatiles were removed *in vacuo*. The crude product was treated with a small amount of *n*-hexane (0.5 mL), the solution filtered through a glass fibre and the clear solution was stored at –35 °C to afford colourless crystals. The solid was then washed with *n*-hexane (2 × 0.5 mL) to afford the desired product **Pt-Zn** as a colourless microcrystalline solid (15 mg, 0.013 mmol, 63% yield).

^{1}H NMR (400 MHz, C_6D_6) δ (ppm): –6.05 (s, $^1J_{H\text{-}Pt}$ = 772 Hz (satellites), 4H, PtH_4), 1.05 (d, $^3J_{H\text{-}H}$ = 6.4 Hz, 24H, CHMe_2), 1.14 (d, $^3J_{H\text{-}H}$ = 6.9 Hz, 24H, CHMe_2), 1.68 (s, 12H, *Me*), 3.16 (sept., $^3J_{H\text{-}H}$ = 6.5 Hz, 8H, C*H*Me_2), 4.94 (s, 2H, β-C*H*), 6.98–7.14 (series of overlapping m, 12H, *Ar*).

***T_1* relaxation time** (PtH_4 signal, 298 K): 0.64 s.

^{13}C{^{1}H} NMR (101 MHz, C_6H_6) δ (ppm): 23.4 (4xCH_3), 24.3 (8xCH_3), 25.1 (8xCH_3), 28.3 (8xCH), 95.6 (2xCH), 123.8 (8xCH), 125.8 (4xCH), 142.0 (8xC), 143.3 (4xC), 168.2 (4xC).

^{195}Pt NMR (108 Hz, C_6H_6) δ (ppm): –6079.2 (quint., $^1J_{H\text{-}Pt}$ = 772 Hz).

ATR IR (cm^{-1}): 3055, 2956, 2922, 2863, 1735, 1698, 1547, 1521, 1457, 1437, 1396, 1316, 1178, 1019, 794, 755.

Anal. Calc. ($C_{58}H_{86}N_4PtZn_2$): C, 59.79; H, 7.44; N, 4.81. Found: C, 59.74; H, 7.56; N, 4.73.

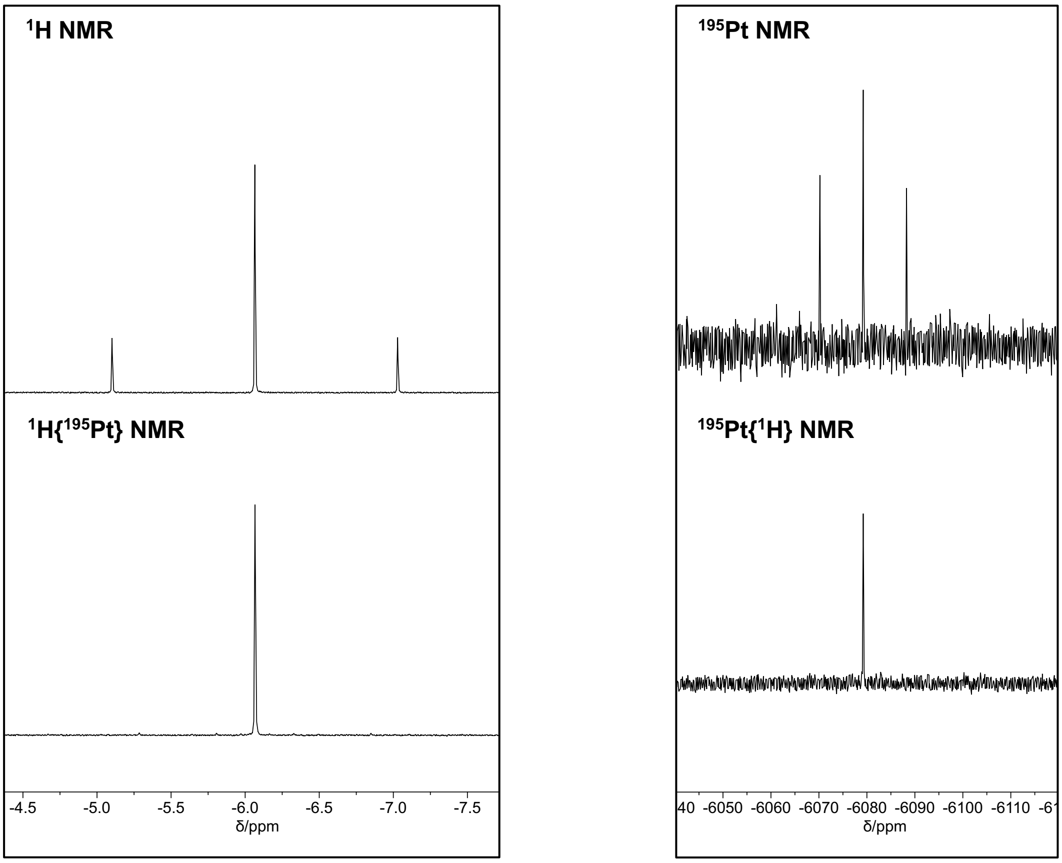

Figure S10. *Stacked ^1H (left) and ^{195}Pt (right) NMR spectra showing the coupled (top) and decoupled (bottom) spectra of **Pt-Zn.***

2.3 Catalytic H/D exchange of magnesium and zinc hydrides

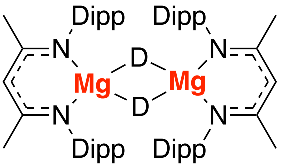

Synthesis of d^2-[3]$_2$: In a J. Young's NMR tube, **[3]$_2$** (26.6 mg, 0.06 mmol, 1 equiv.) and [Pd(PCy$_3$)$_2$] (0.8 mg, 1.2 × 10^{-3} mmol, 0.02 equiv.) were dissolved in C_6D_6 (0.7 mL). The atmosphere in the headspace was evacuated and the tube charged with D_2 gas (1 bar) and the mixture was left 1 day at 25 °C.[i] This evacuation/recharge procedure was repeated three times to maximise D-incorporation. The volatiles were then removed *in vacuo* and the solid washed with cold *n*-hexane (0.5 mL). The solid was then dried *in vacuo* to afford pure d^2-**[1]$_2$** as a white solid (24 mg, 0.054 mmol, 90% yield, 99% D-incorporation).

^{1}H NMR (400 MHz, C_6D_6) δ (ppm): 0.96 (d, $^3J_{H\text{-}H}$ = 6.9 Hz, 24H, CH*Me*$_2$), 1.10 (d, $^3J_{H\text{-}H}$ = 6.9 Hz, 24H, CH*Me*$_2$), 1.48 (s, 12H, *Me*), 3.04 (sept., $^3J_{H\text{-}H}$ = 6.9 Hz, 8H, C*H*Me$_2$), 4.82 (s, 2H, β-C*H*), 6.98–7.11 (m, 12H, *Ar*).

^{2}H NMR (61.4 MHz, C_6H_6) δ (ppm): 4.05 (s, 2H, Mg-*D*).

---

[i] After the first charge with D_2, both H_2 and HD gas can be identified in the ^{1}H NMR spectra, as well as a small amount of heterometallic complex **5c** which could be a potential intermediate.

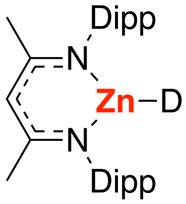

Synthesis of d-4: In a J. Young's NMR tube, **4** (29 mg, 0.06 mmol, 1 equiv.) and [Pd(PCy_3)$_2$] (2 mg, 3×10^{-3} mmol, 0.05 equiv.) were dissolved in C_6D_6 (0.7 mL). The atmosphere in the headspace was evacuated and the tube charged with D_2 gas (1 bar). The mixture was left 1 day at 25 °C.[ii] The atmosphere was then evacuated and recharged with D_2 gas (1 bar) and the mixture left at 25 °C for 1h. This recharge procedure was repeated three times to further increase D-incorporation. The volatiles were then removed *in vacuo* and the solid washed with cold *n*-hexane (0.5 mL). The solid was then dried *in vacuo* to afford pure d-**2** as a white solid (26 mg, 0.054 mmol, 90% yield, 99% D-incorporation).

^{1}H NMR (400 MHz, C_6D_6) δ (ppm): 1.16 (d, $^3J_{H\text{-}H}$ = 6.9 Hz, 12H, CH*Me*$_2$), 1.26 (d, $^3J_{H\text{-}H}$ = 6.8 Hz, 12H, CH*Me*$_2$), 1.68 (s, 6H, *Me*), 3.18 (sept., $^3J_{H\text{-}H}$ = 6.9 Hz, 4H, C*H*Me$_2$), 5.02 (s, 1H, β-C*H*), 7.09–7.14 (m, 6H, *Ar*).

^{2}H NMR (76.8 MHz, C_6H_6) δ (ppm): 4.42 (s, 1H, Zn-*D*).

[ii] Heating the mixture was found to have a very small effect on D-incorporation, and undesired formation of Pd black was observed.

3. X-ray Crystallographic Data

data	Pt-Mg·TMEDA	Pt-Zn
**formula**	$C_{122}H_{188}Mg_4N_{10}Pt$	$C_{58}H_{86}N_4PtZn_2$
**solvent**	$2(C_7H_8)\cdot 2.25(C_6H$	$2(C_6H_{14})$
**formula weight**	2660.39	1337.48
**colour, habit**	colourless needles	colourless blocks
**temperature / K**	173	173
**crystal system**	monoclinic	monoclinic
**space group**	$P2_1/n$ (no. 14)	$I2/m$ (no. 12)
a **/ Å**	13.7691(2)	13.8262(3)
b **/ Å**	25.5240(4)	19.0434(3)
c **/ Å**	21.1334(4)	14.1627(3)
**α / deg**	90	90
**β / deg**	95.1714(17)	108.739(3)
**γ / deg**	90	90
**V / Å^3**	7396.9(2)	3531.34(14)
Z	2 [c]	2 [d]
**D_c / g cm^{-3}**	1.194	1.258
**radiation used**	Cu-Kα	Cu-Kα
**μ / mm^{-1}**	4.017	4.703
**no. of unique reflns**		
**measured (R_{int})**	14179 (0.0436)	3476 (0.0270)
**obs, $\|F_o\| > 4\sigma(\|F_o\|)$**	10088	3288
**completeness (%)** [a]	98.6	98.2
**no. of variables**	728	217
**R_1(obs), wR_2(all)** [b]	0.0442, 0.1150	0.0285, 0.0750
**CCDC code**	2107424	2107425

[a] Completeness to 0.84 Å resolution. [b] $R_1 = \Sigma||F_o| - |F_c||/\Sigma|F_o|$; $wR_2 = \{\Sigma[w(F_o^2 - F_c^2)^2] / \Sigma[w(F_o^2)^2]\}^{1/2}$; $w^{-1} = \sigma^2(F_o^2) + (aP)^2 + bP$. [c] The complex has crystallographic C_i symmetry. [d] The complex has crystallographic C_{2h} symmetry.

Table S1. *Crystal data, data collection and refinement parameters for the structures of* ***Pt-Mg·TMEDA*** *and* ***Pt-Zn***

X-ray crystal structure of Pt-Mg·TMEDA. The structure of **Pt-Mg·TMEDA** was found to sit across a centre of symmetry at the middle of the central C–C bond of the bridging N61-based TMEDA ligand. The C71-based included toluene solvent molecule was found to be disordered. Two orientations were identified of *ca*. 58 and 42% occupancy, their geometries were optimised, the thermal parameters of adjacent atoms were restrained to be similar, and only the non-hydrogen atoms of the major occupancy orientation were refined anisotropically (those of the minor occupancy orientation were refined isotropically). Conversely, the remaining included solvent was found to be highly disordered, and the best approach to handling this diffuse electron density was found to be the SQUEEZE routine of PLATON.[9] This suggested a total of 222 electrons per unit cell, equivalent to 111 electrons per complex. Before the use of SQUEEZE this solvent most resembled hexane (C_6H_{14}, 50 electrons), and 2.25 hexane molecules corresponds to 112.5 electrons, so this was used as the solvent present. As a result, the atom list for the asymmetric unit is low by 0.5 x 2.25(C_6H_{14}) = $C_{6.75}H_{15.75}$ (and that for the unit cell low by $C_{27}H_{63}$) compared to what is actually presumed to be present. The four unique Pt–H–Mg bridging hydrogen atoms were all located from Δ*F* maps and refined freely.

X-ray crystal structure of Pt-Zn. The structure of **Pt-Zn** was found to have crystallographic C_{2h} symmetry with the mirror plane passing through Pt1, Zn1, Zn1A, C2 and C2A, and bisecting each of the two N–Zn–N angles whilst the C_2 axis passes through the platinum centre and bisects the Zn1···Zn1A and N1···N1B vectors. The C21-based included hexane solvent molecule was found to be disordered across a mirror plane, and this was modelled by using one complete, 50% occupancy orientation, with a further orientation being generated by operation of the mirror plane. The geometry of the unique orientation was optimised, and all the non-hydrogen atoms were refined anisotropically. The two unique Pt–H–Zn bridging hydrogen atoms were both located from Δ*F* maps and refined freely.

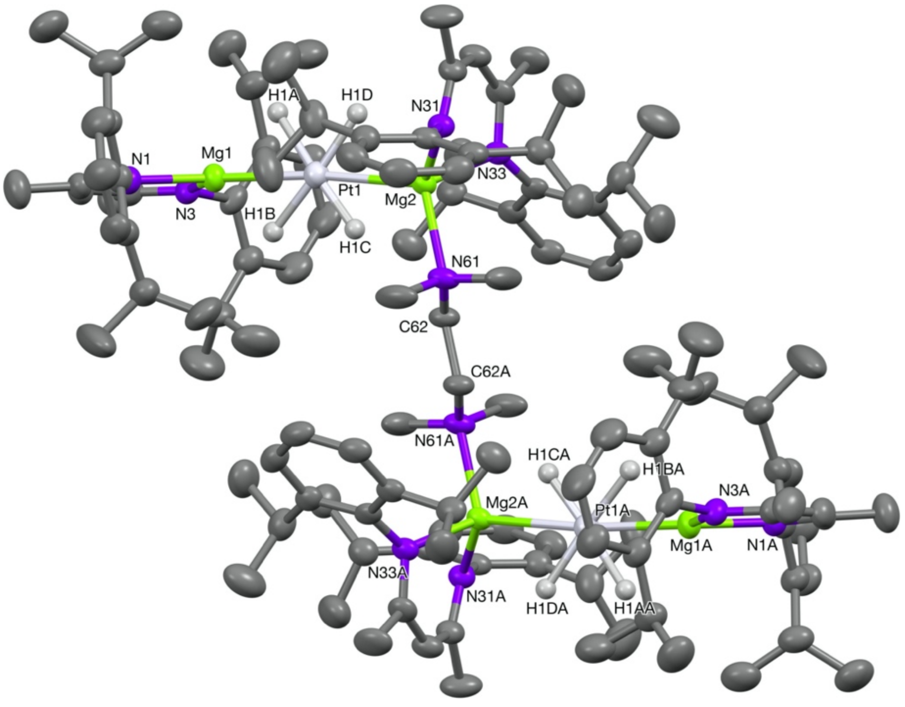

Figure S11. *Structure of* ***Pt-Zn·TMEDA****. Solvent and selected hydrogens removed for clarity. Thermal ellipsoids drawn at 50% probability.*

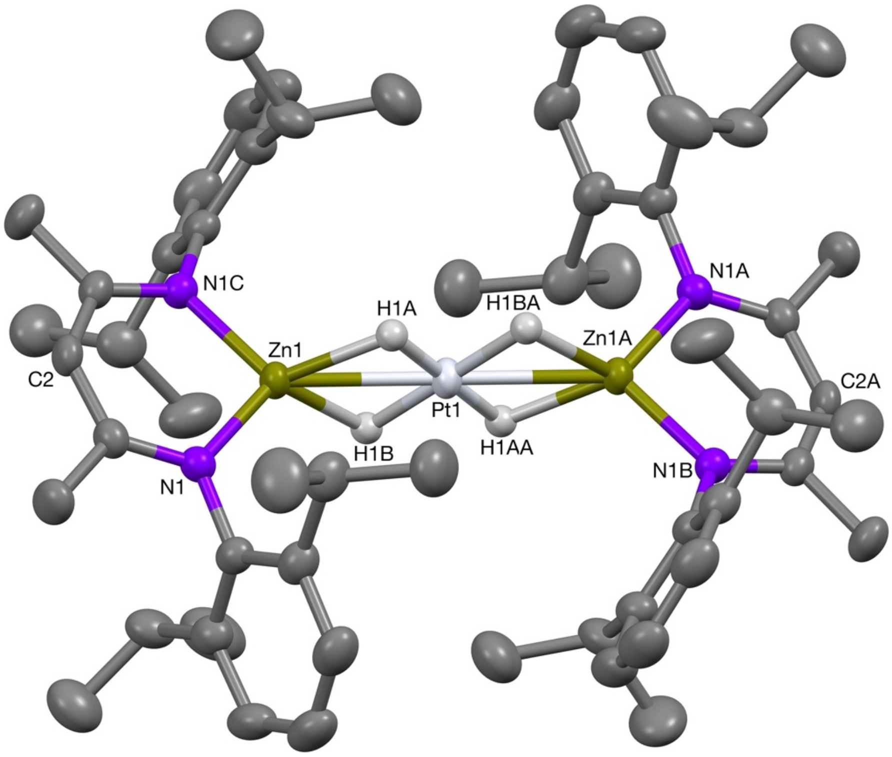

Figure S12. *Structure of* ***Pt-Zn****. Solvent and selected hydrogens removed for clarity. Thermal ellipsoids drawn at 50% probability.*

4.1 Methods

DFT calculations were run using Gaussian 09 (Revision D.01)[10] using the ωB97X hybrid exchange-correlation functional.[11] Geometry optimisations were performed without symmetry constraints, unless otherwise specified, and the nature of the stationary points was confirmed as minima by frequency calculations (no imaginary frequencies). The default numerical integration grid was improved using a pruned grid with 99 radial shells and 590 angular points per shell (int=ultrafine). It should be noted that for the geometry and bonding analysis performed in this work, the use of dispersion or solvent corrections was deemed unnecessary. The level of theory used has previously been benchmarked in our group and shown to reproduce accurately the experimental results.[2-4,12] Geometry optimisations on the complexes were performed with ωB97X/BS1. Frequency calculations using a series of functionals (ωB97x, M06L, B3PW91-GD3BJ) included solvation model (PCM = benzene) were run with BS1. Single point corrections to the SCF energy using the larger basis set, BS2, were applied to the energies reported for the pathway for dissociative H_2 activation (Table S7).

BS1 was built as follows.[1] The SDD effective core potential was used for all metals (SDDAll). The split-valence 6-31G* basis set was used for C and H atoms. The basis set for metal hydrides was expanded by adding one extra set of diffuse functions and three sets of p- and one set of d- polarisation functions, *i.e.* formally [6-31++G(d,3pd)]. The triple-ξ 6-311+G* basis set was used for heteroatoms.

BS2 was built as follows. Pd and Pt were described with the SDD effective core potential, while the other metals and atoms (C, H, N, Mg or Zn) were described using Ahlrichs triple-ξ basis set def2-TZVPP.

NBO analysis was performed at the ωB97X/BS2 level using NBO 6.0 or 3.1 and stated as such.[13] QTAIM analysis was conducted using the AIMAll package.[14] Data are presented from *veryfine mesh* calculations but there are no differences in the appearance of bcps when calculations were run with a *superfine* or *ultrafine mesh*. Standard cut-offs for plotting the data with AIMAll were used. Non-covalent interactions were analysed using the NCIPLOT 3.0 program.[15]

4.2 NBO Calculations

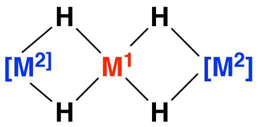

	Ni-Mg	Pd-Mg	Pt-Mg	Ni-Zn	Pd-Zn	Pt-Zn
			NPA charges			
M^1 = Ni,Pd,Pt	-0.58	-0.57	-0.73	-0.45	-0.34	-0.51
M^2 = Mg, Zn	1.50	1.53	1.55	1.18	1.12	1.16
	1.53	1.54	1.55	1.18	1.12	1.16
H	-0.23	-0.24	-0.22	-0.18	-0.18	-0.15
	-0.24	-0.26	-0.22	-0.19	-0.19	-0.16
	-0.25	-0.25	-0.21	-0.18	-0.18	-0.15
	-0.25	-0.26	-0.23	-0.19	-0.19	-0.16
			Wiberg Bond Indices			
M^1---M^2	0.13	0.13	0.14	0.23	0.23	0.26
	0.14	0.13	0.15	0.23	0.23	0.26
M^1–H	0.66	0.62	0.66	0.56	0.48	0.54
	0.67	0.61	0.66	0.57	0.48	0.54
	0.67	0.61	0.66	0.56	0.48	0.54
	0.69	0.61	0.67	0.57	0.48	0.54
M^2–H	0.13	0.12	0.11	0.25	0.28	0.24
	0.11	0.14	0.11	0.26	0.29	0.25
	0.12	0.13	0.11	0.25	0.28	0.24
	0.15	0.13	0.11	0.26	0.29	0.25

Table S2. *NBO data for tetrahydride complexes (Ni,Pd, Pt; Mg, Zn) calculated with BS1 using NBO v 3.1.*

	Ni-Mg	Pd-Mg	Pt-Mg	Ni-Zn	Pd-Zn	Pt-Zn
			NPA Charges			
M^1 = Ni,Pd,Pt	0.16	0.03	-0.09	0.15	0.03	-0.10
M^2 = Mg, Zn	1.68	1.70	1.71	1.50	1.50	1.51
	1.70	1.71	1.72	1.50	1.50	1.51
H	-0.43	-0.40	-0.39	-0.37	-0.34	-0.31
	-0.45	-0.42	-0.39	-0.38	-0.35	-0.32
	-0.42	-0.42	-0.38	-0.37	-0.34	-0.31
	-0.44	-0.42	-0.39	-0.38	-0.35	-0.32
			Wiberg Bond Indices			
M^1---M^2	0.05	0.04	0.05	0.07	0.08	0.09
	0.05	0.04	0.05	0.07	0.08	0.09
M^1–H	0.43	0.40	0.43	0.39	0.38	0.41
	0.43	0.40	0.43	0.39	0.37	0.41
	0.43	0.40	0.43	0.39	0.38	0.41
	0.41	0.40	0.44	0.39	0.37	0.41
M^2–H	0.14	0.12	0.13	0.20	0.20	0.17
	0.12	0.13	0.13	0.21	0.21	0.18
	0.13	0.12	0.11	0.20	0.20	0.17
	0.15	0.13	0.12	0.21	0.21	0.18

Table S3. *NBO data for tetrahydride complexes (Ni,Pd, Pt; Mg, Zn) calculated with BS1 using NBO v 6.0*

	Ni-Mg	Pd-Mg	Pt-Mg	Ni-Zn	Pd-Zn	Pt-Zn
			NPA Charges			
M^1 = Ni,Pd,Pt	0.19	0.03	-0.10	0.15	0.03	-0.10
M^2 = Mg, Zn	1.69	1.68	1.70	1.53	1.53	1.54
	1.69	1.68	1.70	1.53	1.53	1.54
H	-0.45	-0.40	-0.38	-0.38	-0.35	-0.32
	-0.45	-0.41	-0.38	-0.39	-0.35	-0.33
	-0.44	-0.41	-0.38	-0.38	-0.35	-0.32
	-0.46	-0.41	-0.38	-0.39	-0.36	-0.33
			Wiberg Bond Indicies			
M^1---M^2	0.05	0.06	0.07	0.07	0.08	0.09
	0.05	0.06	0.07	0.07	0.08	0.09
M^1–H	0.41	0.38	0.42	0.39	0.37	0.41
	0.42	0.39	0.43	0.39	0.37	0.41
	0.41	0.39	0.43	0.39	0.37	0.41
	0.42	0.39	0.43	0.39	0.37	0.41
M^2–H	0.13	0.13	0.11	0.19	0.19	0.17
	0.12	0.14	0.12	0.19	0.20	0.17
	0.12	0.13	0.11	0.19	0.19	0.17
	0.14	0.14	0.12	0.20	0.20	0.17

Table S4. *NBO data for tetrahydride complexes (Ni,Pd, Pt; Mg, Zn) calculated with BS2 using NBO v 6.0*

4.3 AIM Calculations

	Ni-Mg	Pd-Mg	Pt-Mg	Ni-Zn	Pd-Zn	Pt-Zn
M^1---M^2	-	-	-	-	-	0.06(0.13)
						0.06(0.13)
M^1–H	0.12(0.17)	0.13(0.14)	0.11(0.22)	0.12(0.16)	0.13(0.13)	
	0.12(0.17)	0.13(0.14)	0.11(0.22)	0.11(0.16)	0.13(0.13)	
	0.12(0.17)	0.13(0.14)	0.11(0.22)	0.11(0.16)	0.13(0.13)	
	0.12(0.17)	0.13(0.14)	0.11(0.22)	0.12(0.16)	0.13(0.13)	
M^2–H	0.03 (0.13)	0.03(0.13)	0.03(0.13)	0.07(0.14)	0.06(0.12)	0.06(0.12)
	0.04 (0.16)	0.03(0.13)	0.03(0.13)	0.07(0.15)	0.07(0.13)	0.06(0.12)
	0.03(0.12)	0.03(0.13)	0.03(0.13)	0.07(0.15)	0.06(0.12)	0.06(0.12)
	0.03(0.12)	0.03(0.13)	0.03(0.13)	0.07(0.14)	0.07(0.13)	0.06(0.12)

Table S5. *AIM parameters* $\rho(r)$ *and* $\nabla^2\rho\ (r)$ *in parentheses from in a.u calculated with BS1 using AIMAll.*

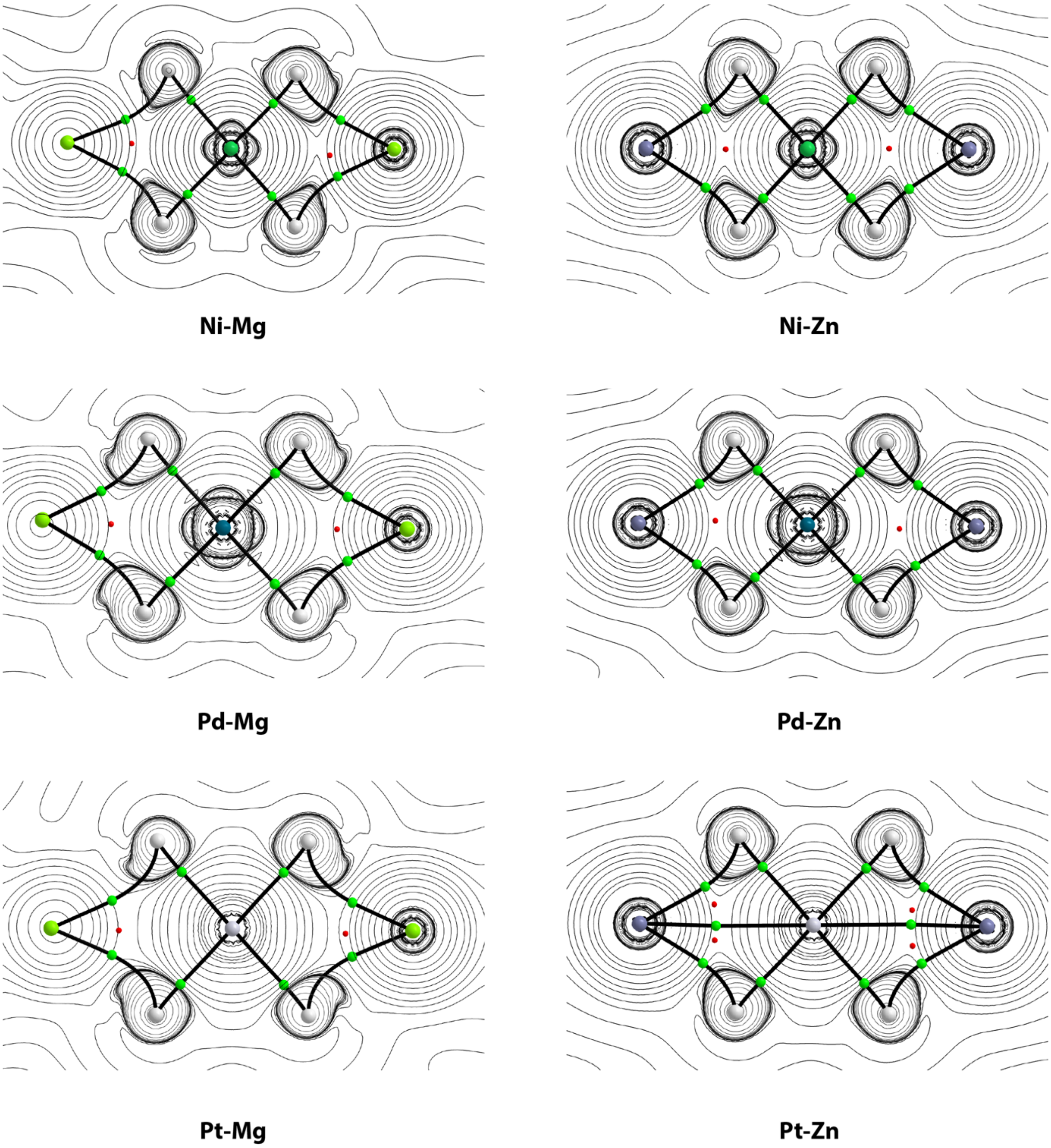

Figure S13. *AIM images for the series of heterometallic hydrides showing key bond critical points (BCPs, green spheres). Associated BCP electron densities, ρ(r) and $\nabla^2\rho$ (r), are given in Table S5.*

4.4 NCI Plot Calculations

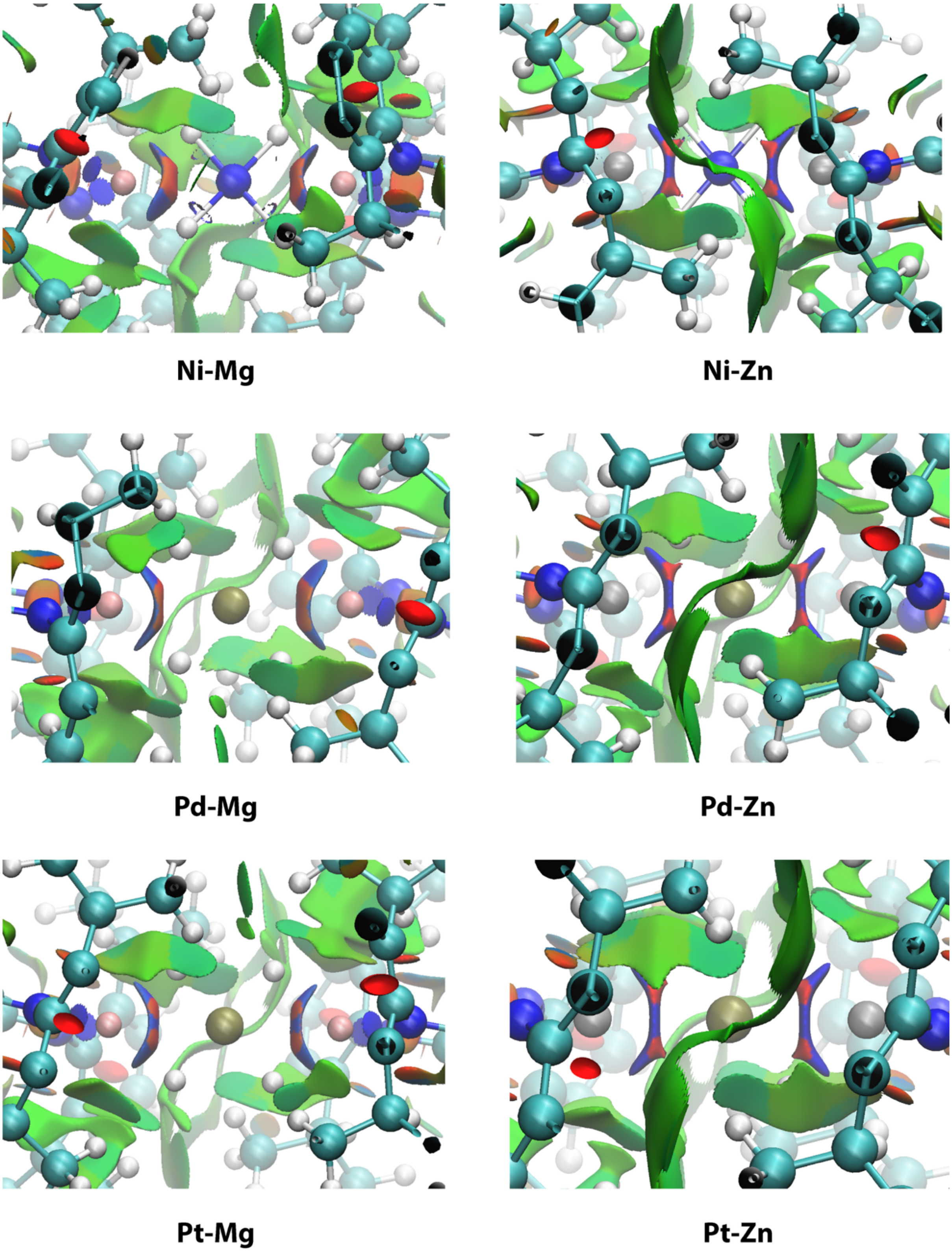

Figure S14. *NCIPlot for the series of heterometallic hydrides showing regions of attractive (blue, green) and repulsive (red) interactions.*

4.5 Molecular Orbital Analysis

The MO diagram was constructed by running DFT calculations on the isolated ligand SALC alongside the dicationic model $[PtMg_2H_4]^{2+}$. Frequency calculations were used to derived shapes and energies of molecular orbitals. AO contributions were derived from NBO analysis. These results were compared with the theoretical MO diagram derived from group theory.

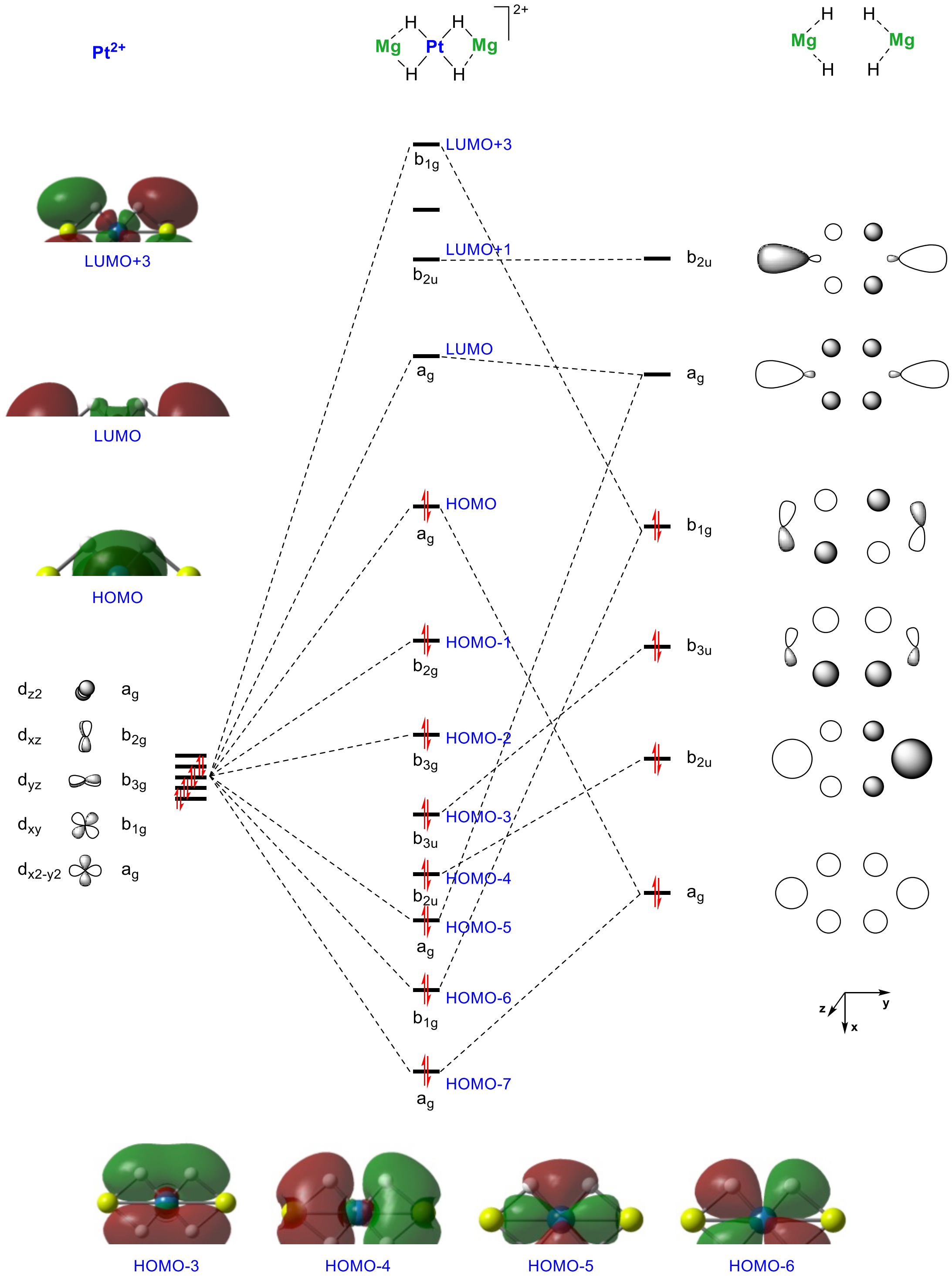

***Figure S15**. Qualitative MO diagram for D_{2h}-symmetric $[PtH_4Mg_2]^{2+}$ annotated with selected calculated MOs.*

4.6 Mechanism of H_2 Activation

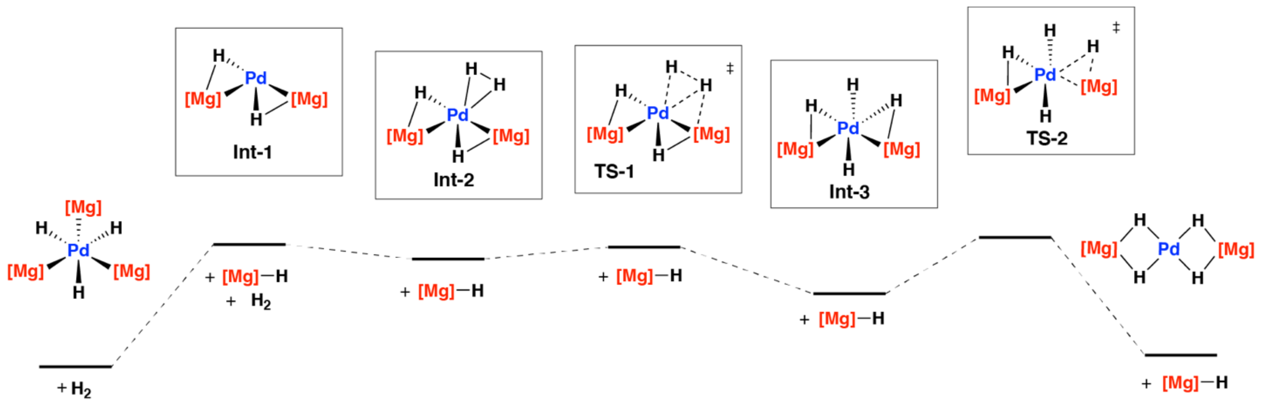

Figure S16. *Calculated dissociative reaction pathway for H_2 activation. Geometries optimised with wB97X / BS1.*

Method	1 + H_2	Int-1 + 2 + H_2	Int-2 + 2	TS-1 + 2	Int-3 + 2	TS-2 + 2	Pd-Mg + 2
ωB97X	0	14.1	5.6	16.2	12.0	19.0	5.6
ωB97X-D	0	29.9	29.9	35.9	30.4	36.2	27.5
M06L	0	21.2	24.6	26.5	22.8	30.5	22.2
B3PW91-GD3BJ	0	33.7	33.0	37.9	33.1	38.6	31.3

Table S6. *Gibbs energies in kcal mol^{-1} for calculated dissociative reaction pathway for H_2 activation with **1**. Geometries optimised with wB97X / BS1. In all cases a solvent correction (PCM = benzene) was included.*

Method	1 + H_2	Int-1 + 2 + H_2	Int-2 + 2	TS-1 + 2	Int-3 + 2	TS-2 + 2	Pd-Mg + 2
ωB97X / BS1	0	14.1	5.6	16.2	12.0	19.0	5.6
ωB97X / BS2	0	10.3	3.3	13.6	9.6	15.7	2.7

Table S7. *Gibbs energies in kcal mol^{-1} for calculated dissociative reaction pathway for H_2 activation with **1**. Geometries optimised with wB97X / BS1. In all cases a solvent correction (PCM = benzene) was included.*

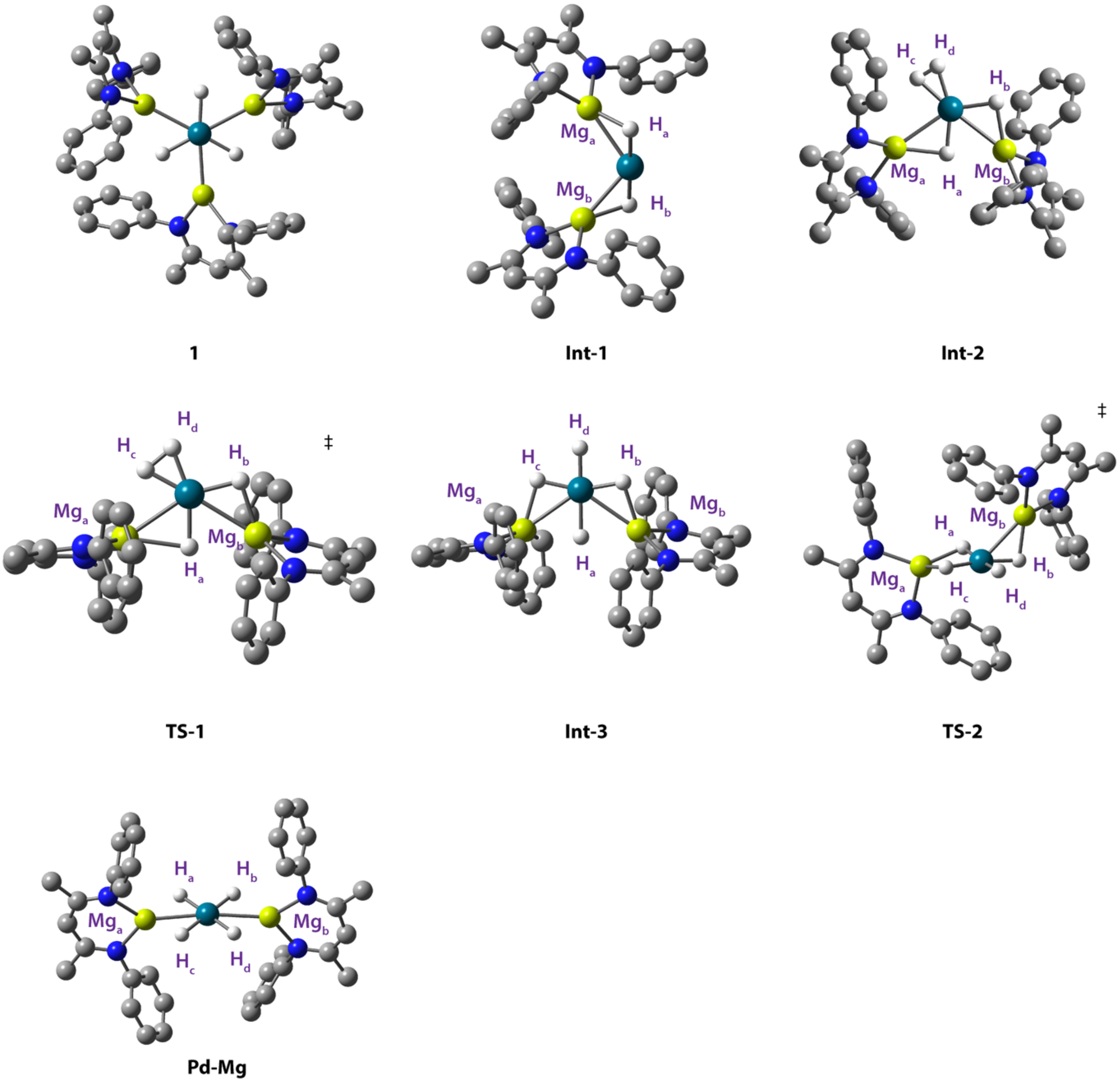

***Figure S17**. Stationary points calculated for H_2 activation with **1**. Geometries optimised with wB97X / BS1.*

Length (Å)	Mg_a–H_a	Mg_b–H_b	H_c–H_d	Mg_b–H_a	Mg_a–H_c
**Int-1**	1.87	1.87	–	–	–
**Int-2**	1.99	1.89	0.90	2.19	2.76
**TS-1**	2.11	1.89	1.14	2.26	2.31
**Int-3**	2.15	1.92	2.05	2.15	1.92
**TS-2**	1.95	1.86	–	–	1.98

***Table S8**. Key bond lengths (Å) in stationary points for H_2 activation with **1**. Geometries optimised with wB97X / BS1.*

NPA	Pd	Mg_a	Mg_b	H_a	H_b	H_c	H_d
**Int-1**	–0.37	1.62	1.62	–0.52	–0.52	-	-
**Int-2**	–0.17	1.64	1.69	–0.59	–0.65	–0.10	–0.01
**TS-1**	–0.16	1.67	1.70	–0.57	–0.60	–0.21	–0.04
**Int-3**	–0.07	1.72	1.72	–0.58	–0.44	–0.44	–0.12
**TS-2**	–0.05	1.70	1.71	–0.53	–0.42	–0.43	–0.23

Table S9. *NPA charges for selected stationary points optimised with wB97X / BS1. Single point NBO v6.0 with BS2.*

WBI	Mg_a–H_a	Mg_b–H_b	H_c–H_d	Mg_b–H_a	Mg_a–H_c
**Int-1**	0.22	0.22	-	-	-
**Int-2**	0.17	0.21	0.82	0.05	0.06
**TS-1**	0.14	0.17	0.45	0.06	0.09
**Int-3**	0.09	0.13	0.12	0.09	0.13
**TS-2**	0.13	0.14	0.09	0.01	0.12

Table S10. *WBI for selected stationary points optimised with wB97X / BS1. NBO v6.0 with BS2.*

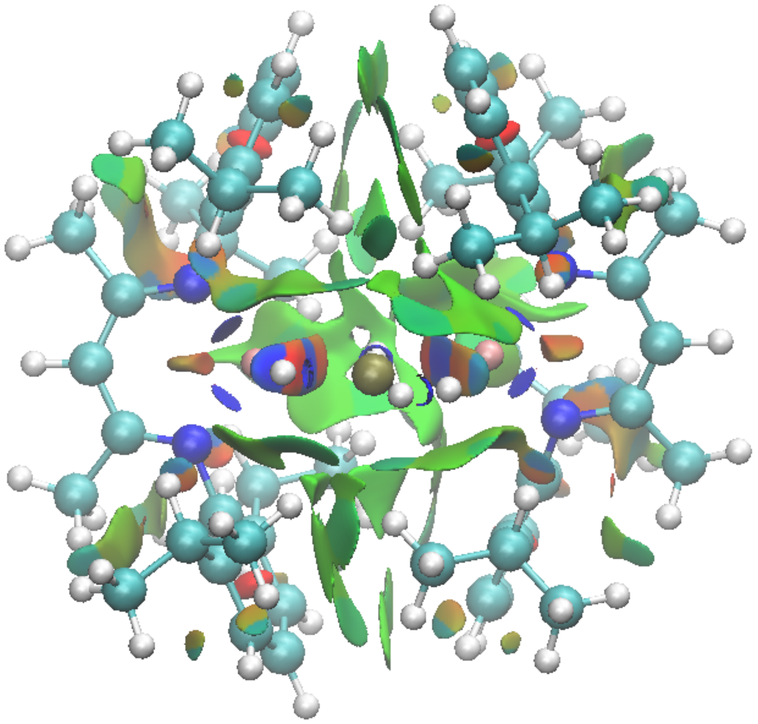

Figure S18. *NCI plot on **TS-1**. Geometries optimised with wB97X / BS1.*

All attempts to identify an associative pathway by coordination of H_2 to the axial position of **1** were unsuccessful. A stable dihydrogen complex could not be identified, not could a viable TS for direct H2 addition to **1**. A TS for H_2 splitting from an isomer of **Int-1** was identified (Figure S13). This TS led to a degenerate exchange process between the hydrides in **Pd-Mg** but at 26.1 kcal mol^{-1} (*wB97X / BS1*) is higher in energy than the dissociative pathway presented in Figure S11.

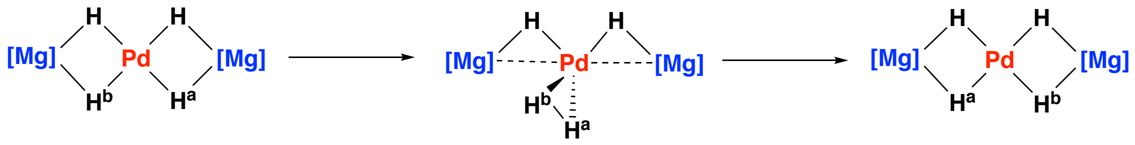

Figure S19. *Degenerate exchange process from isomer of* ***Int-1***. *Geometries optimised with wB97X / BS1.*

4.6 XYZ Coordinates

1.log

SCF (wB97x) = -3849.61939049
E(SCF)+ZPE(0 K)= -3847.650198
H(298 K)= -3847.539260
G(298 K)= -3847.795304
Lowest Frequency = 13.1974cm-1

Pd	-0.142437	0.035076	0.159628
Mg	0.152961	2.182311	-1.156418
Mg	1.870457	-1.344571	0.883388
Mg	-2.561649	-0.792369	0.239135
H	-0.510354	-1.476194	0.882227
H	-1.465262	0.957724	-0.416464
N	1.422950	2.604745	-2.725542
C	1.128498	3.543456	-3.616792
C	0.092113	4.486366	-3.471691
H	-0.016417	5.178175	-4.298967
C	-0.673185	4.799705	-2.328527
N	-0.757333	4.044036	-1.238078
C	1.974316	3.680764	-4.867608
H	3.003362	3.954108	-4.610681
H	1.567700	4.442620	-5.535565
H	2.026200	2.728698	-5.406363
C	-1.401286	6.130020	-2.388895
H	-2.483819	5.993943	-2.334216
H	-1.162904	6.662716	-3.311193
H	-1.121619	6.759809	-1.537726
C	2.693681	1.948445	-2.854055
C	3.810539	2.528279	-2.217398
C	5.056715	1.917855	-2.360481
H	5.927740	2.364788	-1.884479
C	5.204487	0.745795	-3.088616
H	6.183750	0.283565	-3.187174
C	4.091312	0.165834	-3.682143
H	4.203397	-0.759037	-4.245218
C	2.828411	0.752804	-3.586050
C	3.703056	3.794948	-1.377546
H	2.660132	4.129089	-1.398530
C	4.076133	3.515662	0.083983
H	3.441996	2.735556	0.524727
H	3.971429	4.424245	0.690264
H	5.116394	3.179484	0.173300
C	4.555700	4.933991	-1.949873
H	4.422524	5.847251	-1.357283
H	4.277663	5.161585	-2.985483
H	5.622990	4.681631	-1.937250
C	1.641683	0.086866	-4.264347
H	0.818685	0.808362	-4.264340
C	1.922011	-0.284967	-5.725197
H	2.285582	0.573636	-6.302306
H	1.004664	-0.651724	-6.200917
H	2.670450	-1.082189	-5.804326
C	1.187192	-1.147345	-3.474432
H	1.972833	-1.914823	-3.452904
H	0.290304	-1.593148	-3.925653
H	0.949365	-0.883803	-2.435454
C	-1.475796	4.588596	-0.120060
C	-2.884941	4.547462	-0.064090
C	-3.531625	5.224878	0.971806
H	-4.619461	5.222231	1.010371
C	-2.818796	5.887708	1.959726
H	-3.341199	6.417182	2.752721
C	-1.431395	5.834149	1.950518
H	-0.876487	6.315314	2.751380
C	-0.739105	5.181942	0.928921
C	-3.733304	3.746404	-1.045836
H	-3.066713	3.330584	-1.811300
C	-4.812920	4.579523	-1.750236
H	-5.540332	4.977027	-1.032559
H	-5.364521	3.953599	-2.462096
H	-4.398086	5.427684	-2.304260
C	-4.402830	2.574086	-0.312114
H	-3.658560	1.945523	0.189543
H	-4.973586	1.949072	-1.010215
H	-5.099860	2.937128	0.453977
C	0.785630	5.149566	0.949167
H	1.107540	4.236741	0.430169
C	1.359799	5.075942	2.367937
H	1.247719	6.025986	2.904394
H	2.430119	4.844897	2.329243
H	0.870001	4.289800	2.953242
C	1.387558	6.337458	0.187056
H	1.111511	6.322380	-0.873059
H	2.482971	6.318595	0.247344
H	1.041914	7.285045	0.619334
N	3.071688	-1.205258	2.559958
C	3.302492	-2.298477	3.268775
C	3.062448	-3.607437	2.797620
H	3.231286	-4.398224	3.519673
C	2.864185	-4.037411	1.469008
N	2.616798	-3.233184	0.438500
C	3.900062	-2.181761	4.654998
H	4.920881	-1.787851	4.593870
H	3.933420	-3.152366	5.153973
H	3.326520	-1.482411	5.271460
C	2.977254	-5.533598	1.247578
H	1.993878	-5.949974	1.006062
H	3.352891	-6.035598	2.141372
H	3.638872	-5.764380	0.407943
C	3.550452	0.058824	3.036195
C	4.842079	0.488214	2.669591
C	5.256926	1.766674	3.046289
H	6.250413	2.109625	2.762323

C	4.425256	2.609949	3.770761
H	4.761341	3.606130	4.048959
C	3.167298	2.163232	4.152775
H	2.522586	2.816553	4.737864
C	2.714720	0.888173	3.808615
C	5.794358	-0.396476	1.877210
H	5.304056	-1.362326	1.713028
C	6.088705	0.211494	0.503062
H	5.169991	0.363317	-0.075027
H	6.752618	-0.441236	-0.077194
H	6.583800	1.186595	0.599776
C	7.097487	-0.661654	2.641313
H	7.667649	0.262567	2.795070
H	7.734526	-1.355618	2.079468
H	6.906396	-1.101340	3.626893
C	1.346296	0.429144	4.283427
H	1.226016	-0.626668	4.014489
C	1.205260	0.535448	5.807561
H	1.998947	-0.010153	6.331792
H	0.239662	0.123504	6.121894
H	1.246163	1.579536	6.141136
C	0.222218	1.202709	3.592819
H	0.238879	2.263454	3.879199
H	-0.747359	0.786940	3.881665
H	0.302077	1.144208	2.498242
C	2.672512	-3.787847	-0.883948
C	1.537268	-4.388492	-1.459539
C	1.628348	-4.907695	-2.753709
H	0.751912	-5.368307	-3.205145
C	2.811438	-4.845870	-3.469985
H	2.869932	-5.261256	-4.473009
C	3.923925	-4.237368	-2.900070
H	4.842491	-4.184345	-3.476353
C	3.881055	-3.691971	-1.615892
C	0.200427	-4.471223	-0.739500
H	0.331298	-4.083241	0.277131
C	-0.314702	-5.913128	-0.633976
H	-0.532032	-6.334271	-1.623093
H	-1.245625	-5.945812	-0.054226
H	0.409763	-6.576949	-0.149887
C	-0.836550	-3.587582	-1.440285
H	-0.487999	-2.553039	-1.519502
H	-1.777465	-3.588224	-0.874082
H	-1.059254	-3.948253	-2.452571
C	5.115514	-3.002450	-1.035767
H	4.765189	-2.087531	-0.538129
C	6.127884	-2.591372	-2.109377
H	6.897828	-1.948768	-1.668352
H	6.640797	-3.464317	-2.531943
H	5.654922	-2.040174	-2.927048
C	5.847338	-3.850462	0.016525
H	6.768076	-3.341148	0.329604
H	5.249329	-4.020226	0.915105
H	6.131344	-4.823892	-0.403785
N	-3.852474	-0.978723	1.854087
C	-5.168302	-1.005412	1.719395
C	-5.841531	-0.870121	0.485628
H	-6.917071	-0.751551	0.561315
C	-5.343047	-1.007120	-0.816999
N	-4.070103	-1.264460	-1.127387
C	-6.068971	-1.183183	2.928129
H	-5.641445	-1.895931	3.638779
H	-7.053434	-1.539534	2.617264
H	-6.204808	-0.236421	3.461157
C	-6.372381	-0.836867	-1.921353
H	-6.245767	0.158296	-2.365016
H	-7.389029	-0.900881	-1.527044
H	-6.257322	-1.566500	-2.725305
C	-3.298839	-1.053659	3.172096
C	-2.620275	-2.228213	3.561641
C	-2.087995	-2.292270	4.850259
H	-1.569909	-3.190319	5.174592
C	-2.214147	-1.227651	5.734164
H	-1.805758	-1.302572	6.739379
C	-2.854054	-0.065916	5.325125
H	-2.924510	0.773244	6.013686
C	-3.396400	0.051384	4.043819
C	-2.505757	-3.420659	2.618049
H	-2.391938	-3.029144	1.598902
C	-1.276064	-4.292916	2.883454
H	-1.356968	-4.838034	3.831665
H	-0.355191	-3.697072	2.906614
H	-1.175660	-5.042901	2.089921
C	-3.781777	-4.272942	2.629798
H	-3.967381	-4.678905	3.632089
H	-3.684147	-5.116890	1.934329
H	-4.659185	-3.690579	2.328010
C	-4.025871	1.375054	3.619188
H	-4.621138	1.203677	2.716863
C	-4.961534	1.958008	4.686240
H	-5.701586	1.226800	5.031919
H	-5.498436	2.823524	4.281448
H	-4.405193	2.305289	5.564725
C	-2.951727	2.404902	3.248397
H	-2.319951	2.639659	4.114020
H	-3.415813	3.335953	2.906010
H	-2.297581	2.041924	2.446087
C	-3.814799	-1.778317	-2.443205
C	-3.388930	-0.939282	-3.489882
C	-3.147233	-1.501213	-4.747446
H	-2.822636	-0.858469	-5.563774
C	-3.331766	-2.854665	-4.979844
H	-3.148897	-3.272140	-5.966859
C	-3.755904	-3.675213	-3.940731
H	-3.895188	-4.736645	-4.129083
C	-3.995500	-3.166693	-2.664740
C	-3.210476	0.562702	-3.316582
H	-3.404623	0.805964	-2.266532
C	-4.195432	1.361102	-4.181186
H	-4.012833	1.187122	-5.248682

H	-4.080110	2.436934	-3.996875
H	-5.236498	1.088655	-3.977534
C	-1.775744	0.990492	-3.635556
H	-1.062863	0.417283	-3.033501
H	-1.635036	2.064773	-3.445117
H	-1.531224	0.818415	-4.691771
C	-4.450539	-4.109421	-1.549953
H	-4.089096	-3.691242	-0.600527
C	-3.880306	-5.526664	-1.692630
H	-4.387379	-6.085314	-2.488959
H	-4.037454	-6.084284	-0.761396
H	-2.809121	-5.523545	-1.911605
C	-5.980974	-4.217376	-1.450332
H	-6.449362	-3.283598	-1.131852
H	-6.252921	-4.986218	-0.716898
H	-6.408803	-4.508664	-2.418042
H	1.480867	0.548385	-0.055627

2.log

SCF (wB97x) = A.U.
E(SCF)+ZPE(0 K)= -1239.862511
H(298 K)= -1239.825389
G(298 K)= -1239.932540
Lowest Frequency = 9.2663cm-1

Mg	2.892051	-1.343715	0.588145
N	3.433289	-1.269303	2.545073
C	3.624011	-2.338286	3.308784
C	3.505753	-3.668036	2.860635
H	3.684395	-4.430060	3.610668
C	3.227754	-4.146686	1.565857
N	2.974337	-3.372017	0.517923
C	4.006541	-2.144621	4.760627
H	4.951349	-1.594451	4.836743
H	4.114742	-3.101459	5.274767
H	3.251118	-1.545904	5.281670
C	3.236497	-5.649408	1.383053
H	2.273626	-5.995505	0.991018
H	3.439647	-6.163623	2.324283
H	3.997642	-5.941081	0.650429
C	3.631764	0.040439	3.094021
C	4.896034	0.651193	2.978332
C	5.047857	1.962423	3.430274
H	6.015972	2.450654	3.340941
C	3.982305	2.659229	3.984463
H	4.117714	3.681597	4.328074
C	2.741651	2.045557	4.091932
H	1.908000	2.598506	4.519615
C	2.542657	0.735762	3.653470
C	6.071568	-0.069795	2.332030
H	5.813408	-1.131733	2.252095
C	6.308962	0.458338	0.910134
H	5.405232	0.383748	0.291409
H	7.111149	-0.101625	0.413911
H	6.596691	1.516549	0.932148
C	7.353576	0.024099	3.166411
H	7.724007	1.053905	3.228890
H	8.146419	-0.581646	2.712821
H	7.192774	-0.335882	4.189139
C	1.158734	0.107991	3.744923
H	1.262723	-0.965352	3.550398
C	0.533386	0.259966	5.135931
H	1.192186	-0.135604	5.917682
H	-0.417671	-0.282496	5.185407
H	0.324384	1.309180	5.374987
C	0.233333	0.688736	2.666403
H	0.082293	1.763779	2.823046
H	-0.748756	0.201337	2.692159
H	0.653754	0.563905	1.660392
C	2.754408	-3.966348	-0.768395
C	1.443736	-4.273910	-1.180786
C	1.248224	-4.760278	-2.474125
H	0.240514	-4.995335	-2.810166
C	2.315502	-4.941727	-3.343189
H	2.143649	-5.318274	-4.348331
C	3.603080	-4.634241	-2.923904
H	4.435016	-4.770899	-3.611477
C	3.846637	-4.145250	-1.640137
C	0.246667	-4.045222	-0.268357
H	0.624891	-3.866362	0.744407
C	-0.682090	-5.262461	-0.198858
H	-1.164332	-5.460966	-1.163076
H	-1.477408	-5.091162	0.535549
H	-0.136949	-6.167195	0.094208
C	-0.528989	-2.793814	-0.703305
H	0.115593	-1.906092	-0.732661
H	-1.360643	-2.592978	-0.017199
H	-0.944425	-2.925586	-1.709846
C	5.263659	-3.771893	-1.225412
H	5.270859	-3.631744	-0.138652
C	5.677215	-2.440187	-1.867745
H	6.671404	-2.131298	-1.522077
H	5.710071	-2.530181	-2.960399
H	4.967518	-1.636259	-1.632811
C	6.283295	-4.869280	-1.548652
H	7.268637	-4.600132	-1.151277
H	5.989907	-5.830013	-1.110114
H	6.395454	-5.015376	-2.629257
H	2.501820	-0.142652	-0.568131

H2.log

SCF (wB97x) = -1.17163569765
E(SCF)+ZPE(0 K)= -1.161514
H(298 K)= -1.158210
G(298 K)= -1.173006
Lowest Frequency = 4442.7012cm-1

H	-1.162890	9.863574	6.611966

H -1.402479 9.874806 5.907411

Int-1.log

SCF (wB97x) = -2609.04265041
E(SCF)+ZPE(0 K)= -2607.728384
H(298 K)= -2607.653441
G(298 K)= -2607.839101
Lowest Frequency = 14.9729cm-1

Pd	-0.153202	0.118591	0.447166
Mg	0.018931	2.306950	-0.829825
Mg	-2.556627	-0.632501	0.056721
H	-0.835895	-1.360902	0.028763
N	1.208000	2.413529	-2.486130
C	1.044627	3.330635	-3.423822
C	0.080484	4.364235	-3.367164
H	0.060135	5.014672	-4.234784
C	-0.727317	4.776315	-2.293145
N	-0.882571	4.108442	-1.148840
C	1.978502	3.378456	-4.616774
H	2.867747	3.967976	-4.360696
H	1.492105	3.862585	-5.467358
H	2.321507	2.384916	-4.913146
C	-1.438445	6.102735	-2.477547
H	-2.523144	5.978471	-2.399213
H	-1.205759	6.542887	-3.448858
H	-1.146427	6.808911	-1.692777
C	2.343338	1.537514	-2.533497
C	3.595620	2.009887	-2.091628
C	4.679587	1.130876	-2.092173
H	5.651567	1.479894	-1.750142
C	4.537577	-0.183303	-2.513819
H	5.393108	-0.854122	-2.511520
C	2.176751	0.196080	-2.936218
C	3.784976	3.425316	-1.559420
H	2.904289	4.018867	-1.828090
C	3.873200	3.410475	-0.027327
H	2.987539	2.945181	0.423436
H	3.963088	4.431789	0.364883
H	4.748778	2.840426	0.306838
C	5.007198	4.127105	-2.162828
H	5.044152	5.172463	-1.835093
H	4.979852	4.113304	-3.258570
H	5.944131	3.652955	-1.848272
C	0.826683	-0.333107	-3.401888
H	0.053566	0.230298	-2.866659
C	-1.580420	4.758800	-0.074954
C	-2.983784	4.678707	0.035025
C	-3.618812	5.365420	1.071356
H	-4.702882	5.316724	1.156324
C	-2.895634	6.102136	1.999398
H	-3.407688	6.637523	2.794994
C	-1.510613	6.127714	1.915643
H	-0.940738	6.681704	2.658945
C	-0.832127	5.457320	0.896103
C	-3.824498	3.816635	-0.895617
H	-3.166621	3.430504	-1.680982
C	-4.964888	4.585699	-1.572815
H	-5.701819	4.939509	-0.842074
H	-5.491946	3.933642	-2.280411
H	-4.600680	5.459071	-2.125085
C	-4.384093	2.618079	-0.118490
H	-3.571703	2.032690	0.332004
H	-4.976708	1.956153	-0.760547
H	-5.031662	2.949749	0.703125
C	0.690826	5.498843	0.879962
H	1.040840	4.883071	0.042672
C	1.267853	4.903976	2.171223
H	0.968838	5.489854	3.048615
H	2.363344	4.894901	2.134819
H	0.922784	3.873753	2.320468
C	1.218924	6.918599	0.642066
H	0.842493	7.332545	-0.300649
H	2.314688	6.916996	0.597642
H	0.917633	7.596197	1.450208
N	-3.814930	-0.784111	1.657282
C	-5.131696	-0.784300	1.530829
C	-5.813198	-0.801027	0.291207
H	-6.894725	-0.784867	0.364333
C	-5.306248	-0.995849	-1.005592
N	-4.013884	-0.992847	-1.330481
C	-6.007232	-0.792330	2.767664
H	-5.679770	-1.559615	3.475780
H	-7.053252	-0.969325	2.509629
H	-5.936872	0.170453	3.285880
C	-6.337014	-1.272630	-2.081263
H	-6.210836	-0.596944	-2.932189
H	-7.351549	-1.166664	-1.692641
H	-6.217654	-2.291015	-2.468017
C	-3.225372	-0.939121	2.956944
C	-2.729216	-2.212361	3.315558
C	-2.156281	-2.371728	4.577613
H	-1.778934	-3.345170	4.877762
C	-2.045065	-1.303425	5.458633
H	-1.595357	-1.448196	6.437774
C	-2.489505	-0.048228	5.072332
H	-2.370308	0.793194	5.751573
C	-3.077998	0.160888	3.823479
C	-2.814564	-3.394868	2.355442
H	-2.711851	-3.000574	1.335918
C	-1.682191	-4.407430	2.546399
H	-1.779829	-4.959496	3.488726
H	-0.702795	-3.916922	2.532845
H	-1.703175	-5.146380	1.737714
C	-4.176580	-4.097303	2.427869
H	-4.358792	-4.483294	3.438680
H	-4.207169	-4.944803	1.730879
H	-4.997664	-3.421679	2.165914
C	-3.469845	1.574286	3.417889

H	-4.059629	1.511230	2.497659
C	-4.330324	2.287483	4.467601
H	-5.222714	1.708342	4.733063
H	-4.655190	3.261825	4.083248
H	-3.769469	2.472757	5.391208
C	-2.213035	2.400590	3.110174
H	-1.597696	2.515435	4.012171
H	-2.481203	3.402179	2.751870
H	-1.590707	1.913813	2.349336
C	-3.633684	-1.449287	-2.638069
C	-3.485106	-0.549075	-3.713039
C	-3.083973	-1.048315	-4.954598
H	-2.974693	-0.362055	-5.792535
C	-2.824147	-2.398138	-5.144305
H	-2.513233	-2.765214	-6.119152
C	-2.960889	-3.273853	-4.075808
H	-2.749799	-4.331529	-4.220997
C	-3.362635	-2.823873	-2.817672
C	-3.750066	0.945090	-3.580030
H	-4.083167	1.135700	-2.554212
C	-4.855400	1.424851	-4.530959
H	-4.551837	1.319401	-5.579338
H	-5.072489	2.485721	-4.355727
H	-5.786409	0.863068	-4.398945
C	-2.472606	1.758867	-3.821426
H	-1.689498	1.501847	-3.097850
H	-2.666293	2.835082	-3.742654
H	-2.060946	1.570077	-4.820436
C	-3.484914	-3.832805	-1.681873
H	-3.870007	-3.310519	-0.798736
C	-2.114041	-4.418154	-1.317855
H	-1.672756	-4.950050	-2.169762
H	-2.210614	-5.133220	-0.491499
H	-1.409309	-3.634959	-1.012312
C	-4.480854	-4.951305	-2.013006
H	-5.468217	-4.548944	-2.267020
H	-4.599758	-5.620447	-1.152552
H	-4.139021	-5.557702	-2.860285
H	0.526235	1.619246	0.831109
C	0.600717	-1.810009	-3.067599
H	0.794449	-2.010512	-2.008026
H	-0.441537	-2.078550	-3.279107
H	1.235618	-2.469504	-3.672828
C	0.621005	-0.091587	-4.903190
H	1.421640	-0.568408	-5.483190
H	-0.335785	-0.518295	-5.226919
H	0.614358	0.976592	-5.147144
C	3.293381	-0.641411	-2.926847
H	3.186378	-1.676145	-3.240727

Int-2.log

SCF (wB97x) = -2610.22825674
E(SCF)+ZPE(0 K)= -2608.901044
H(298 K)= -2608.824696
G(298 K)= -2609.014285
Lowest Frequency = 9.6051cm-1

Pd	-0.278679	0.258258	0.605735
Mg	0.177047	2.357060	-0.824209
Mg	-2.667069	-0.531824	0.075602
H	-1.374444	0.827904	-0.575924
N	1.267491	2.460379	-2.546913
C	1.036717	3.356671	-3.493244
C	0.105071	4.411176	-3.381828
H	0.040700	5.059509	-4.248430
C	-0.632105	4.837177	-2.259702
N	-0.741039	4.164014	-1.117394
C	1.853334	3.321275	-4.768254
H	2.886939	3.623834	-4.562731
H	1.440090	3.996495	-5.520386
H	1.895844	2.308158	-5.179288
C	-1.298448	6.193037	-2.390614
H	-2.364099	6.142743	-2.152070
H	-1.178893	6.595253	-3.398269
H	-0.853205	6.898370	-1.679366
C	2.394222	1.579736	-2.668949
C	3.659246	2.030269	-2.235092
C	4.737796	1.148032	-2.285508
H	5.716619	1.481201	-1.946638
C	4.584866	-0.150492	-2.754458
H	5.437452	-0.824172	-2.788648
C	2.223744	0.263003	-3.135886
C	3.867849	3.432995	-1.677209
H	2.968782	4.023116	-1.886608
C	4.041396	3.387213	-0.153036
H	3.189946	2.897146	0.335036
H	4.138354	4.401843	0.254837
H	4.943486	2.825495	0.119548
C	5.049290	4.156502	-2.334160
H	5.109035	5.189979	-1.973496
H	4.950118	4.182583	-3.425616
H	6.003761	3.671397	-2.098514
C	0.875312	-0.268447	-3.598230
H	0.136652	0.528924	-3.465658
C	-1.431831	4.786793	-0.024720
C	-2.829958	4.655275	0.111174
C	-3.467810	5.318645	1.160842
H	-4.548825	5.238362	1.262473
C	-2.750503	6.066492	2.085051
H	-3.264730	6.578380	2.894640
C	-1.368233	6.134586	1.979170
H	-0.802510	6.696062	2.720122
C	-0.687906	5.501855	0.936916
C	-3.658543	3.770005	-0.810898
H	-2.977805	3.322059	-1.541541
C	-4.740074	4.538871	-1.578969
H	-5.463822	4.999646	-0.895779
H	-5.294105	3.857187	-2.236882
H	-4.316692	5.334172	-2.201814

C	-4.294876	2.629123	-0.006404
H	-3.523766	2.074853	0.540644
H	-4.839766	1.932440	-0.655978
H	-5.007176	3.010602	0.736185
C	0.832342	5.591942	0.888121
H	1.178342	5.051543	-0.001335
C	1.455664	4.917676	2.117971
H	1.150918	5.423299	3.042430
H	2.550091	4.951444	2.062550
H	1.152032	3.866448	2.192489
C	1.318107	7.040028	0.752107
H	0.898503	7.522720	-0.138160
H	2.411085	7.068926	0.669572
H	1.034114	7.641467	1.624110
N	-3.902228	-0.875598	1.665485
C	-5.218435	-0.910233	1.517715
C	-5.879645	-0.948843	0.268779
H	-6.962188	-0.966494	0.324035
C	-5.344693	-1.145039	-1.019370
N	-4.050993	-1.079750	-1.323583
C	-6.109025	-0.933388	2.743625
H	-5.810049	-1.730203	3.431689
H	-7.156387	-1.075195	2.469992
H	-6.017046	0.010554	3.292607
C	-6.342695	-1.507249	-2.099973
H	-6.187807	-0.913832	-3.005042
H	-7.368045	-1.368032	-1.752050
H	-6.215908	-2.558300	-2.385827
C	-3.333215	-1.051350	2.972282
C	-2.860660	-2.334495	3.328315
C	-2.305869	-2.512030	4.596079
H	-1.945916	-3.492711	4.893629
C	-2.187101	-1.452081	5.486208
H	-1.750575	-1.610876	6.469048
C	-2.605592	-0.187485	5.102917
H	-2.479847	0.647566	5.788660
C	-3.176747	0.038958	3.848852
C	-2.936392	-3.505008	2.352678
H	-2.809845	-3.094236	1.341554
C	-1.819716	-4.531970	2.561980
H	-1.944759	-5.086663	3.499242
H	-0.832383	-4.056532	2.574283
H	-1.832640	-5.269093	1.751647
C	-4.303799	-4.200495	2.382852
H	-4.516271	-4.590533	3.386181
H	-4.318099	-5.044646	1.681383
H	-5.113979	-3.520840	2.099975
C	-3.538864	1.461058	3.448433
H	-4.130980	1.411772	2.529019
C	-4.389327	2.186256	4.497809
H	-5.294368	1.622190	4.752996
H	-4.693565	3.168521	4.117012
H	-3.830980	2.355808	5.425833
C	-2.263338	2.262300	3.147610
H	-1.643358	2.351190	4.048819
H	-2.505998	3.273977	2.799488
H	-1.649778	1.770455	2.379661
C	-3.622626	-1.505098	-2.626382
C	-3.468430	-0.580677	-3.680131
C	-3.013882	-1.046590	-4.916441
H	-2.899488	-0.343058	-5.739028
C	-2.708775	-2.384713	-5.120994
H	-2.358347	-2.725051	-6.092271
C	-2.848724	-3.282859	-4.071744
H	-2.596855	-4.329926	-4.227237
C	-3.301254	-2.866241	-2.819615
C	-3.790624	0.901492	-3.536290
H	-4.119918	1.075024	-2.506009
C	-4.929195	1.330857	-4.472293
H	-4.641185	1.219323	-5.524499
H	-5.179260	2.385979	-4.307678
H	-5.838022	0.740403	-4.312982
C	-2.552331	1.771835	-3.787746
H	-1.763383	1.561862	-3.056700
H	-2.801635	2.837574	-3.718954
H	-2.133159	1.596036	-4.786245
C	-3.394519	-3.886840	-1.692519
H	-3.883129	-3.409205	-0.835706
C	-1.990593	-4.320904	-1.249650
H	-1.444398	-4.787141	-2.078811
H	-2.049651	-5.049995	-0.432899
H	-1.390572	-3.469044	-0.902469
C	-4.241943	-5.106112	-2.074529
H	-5.243579	-4.810257	-2.406842
H	-4.354597	-5.773961	-1.212284
H	-3.779686	-5.685750	-2.882216
H	0.968300	1.518306	0.675690
C	0.422430	-1.465711	-2.753047
H	0.399986	-1.208081	-1.685768
H	-0.583288	-1.785037	-3.055064
H	1.098427	-2.321266	-2.879447
C	0.889994	-0.637095	-5.087162
H	1.604939	-1.444375	-5.288744
H	-0.102851	-0.981863	-5.399837
H	1.169254	0.218086	-5.713937
C	3.335386	-0.581791	-3.174661
H	3.215451	-1.600331	-3.539050
H	0.355783	-0.779455	1.788535
H	-0.408354	-1.175366	1.522713

Int-3.log

SCF (wB97x) = -2610.23465417
E(SCF)+ZPE(0 K)= -2608.906977
H(298 K)= -2608.830806
G(298 K)= -2609.020983
Lowest Frequency = 6.4688cm-1

H	-2.467475	9.522089	6.144212
N	0.927305	10.834013	5.685926

C	2.179517	10.606442	5.290175
C	2.671687	9.365007	4.849489
H	3.722071	9.355298	4.581128
C	3.191999	11.733902	5.345433
H	2.772127	12.656232	4.932265
H	4.094381	11.472438	4.789143
H	3.478222	11.950444	6.381054
C	0.636152	12.074446	6.343249
C	-0.054406	13.093449	5.662309
C	-0.339743	14.280181	6.345499
H	-0.874726	15.074241	5.828409
C	0.056646	14.467059	7.659828
H	-0.160284	15.402847	8.170048
C	0.730332	13.448255	8.326243
H	1.027896	13.597908	9.361174
C	1.019564	12.240739	7.694746
C	-0.471982	12.964866	4.204218
H	-0.207552	11.959185	3.859080
C	-1.985477	13.133139	4.031669
H	-2.542131	12.438775	4.673111
H	-2.276727	12.932907	2.993659
H	-2.309984	14.149058	4.287299
C	0.291187	13.958468	3.318539
H	0.067434	14.994104	3.602218
H	0.007584	13.829362	2.267632
H	1.375601	13.817321	3.396643
C	1.696449	11.113260	8.467510
H	2.234694	10.479725	7.755019
C	2.721541	11.605330	9.493966
H	3.446962	12.292095	9.042602
H	3.271936	10.754190	9.910373
H	2.245074	12.123866	10.334181
C	0.646096	10.224887	9.146565
H	0.074861	10.794982	9.889671
H	1.124949	9.379250	9.656451
H	-0.067686	9.818720	8.418727
N	0.743159	7.913995	5.032688
C	2.039131	8.100934	4.840129
C	2.948099	6.906886	4.638868
H	2.522260	6.185595	3.937353
H	3.933112	7.216758	4.284208
H	3.076926	6.382412	5.593613
C	0.218474	6.579109	5.071489
C	-0.268301	5.974931	3.893548
C	-0.795991	4.685150	3.980254
H	-1.163456	4.200999	3.077565
C	-0.872249	4.013177	5.192639
H	-1.291765	3.010703	5.237092
C	-0.416290	4.632035	6.348441
H	-0.493901	4.112093	7.301429
C	0.139982	5.911436	6.309884
C	-0.232752	6.668929	2.537185
H	0.135720	7.689945	2.687797
C	-1.633284	6.770078	1.919575
H	-2.327250	7.293987	2.584669

H	-1.590738	7.328477	0.976813
H	-2.045890	5.776984	1.701428
C	0.723052	5.963760	1.564749
H	0.398630	4.933812	1.371801
H	0.746039	6.491792	0.604424
H	1.747987	5.923777	1.949800
C	0.619861	6.554717	7.603898
H	1.098930	7.508897	7.354723
C	1.660968	5.692802	8.328336
H	2.515600	5.462930	7.681739
H	2.037232	6.215414	9.215816
H	1.231708	4.740745	8.662371
C	-0.566869	6.852294	8.526567
H	-1.086803	5.931480	8.812379
H	-0.231385	7.353465	9.442919
H	-1.308611	7.496885	8.036073
H	-4.663659	9.321534	4.698576
N	-5.678798	11.128197	7.253304
C	-6.821147	10.938654	7.894218
C	-7.317435	9.672684	8.280163
H	-8.311584	9.681148	8.712573
C	-7.660608	12.131374	8.300571
H	-7.176308	12.649663	9.137208
H	-8.656803	11.821057	8.622059
H	-7.752856	12.858036	7.489881
C	-5.236785	12.464346	6.973829
C	-4.412059	13.120815	7.908994
C	-3.945406	14.401563	7.609558
H	-3.297473	14.913090	8.318841
C	-4.292819	15.032160	6.422801
H	-3.931029	16.035378	6.209290
C	-5.099356	14.370940	5.506500
H	-5.361620	14.864167	4.572568
C	-5.573693	13.080664	5.750672
C	-3.997918	12.464247	9.218983
H	-4.527207	11.507917	9.304806
C	-2.494043	12.169982	9.219367
H	-2.205676	11.536417	8.369847
H	-2.197977	11.658274	10.143444
H	-1.909913	13.093170	9.138908
C	-4.379761	13.312368	10.438239
H	-3.838508	14.265884	10.449186
H	-4.132266	12.780796	11.364798
H	-5.451988	13.539088	10.452891
C	-6.436531	12.398756	4.695650
H	-6.641428	11.376643	5.033672
C	-7.783483	13.113060	4.516978
H	-8.357146	13.151380	5.449628
H	-8.393363	12.592889	3.769299
H	-7.639213	14.144083	4.171401
C	-5.708096	12.302257	3.349009
H	-5.508351	13.297077	2.931225
H	-6.323045	11.753573	2.625655
H	-4.755938	11.770685	3.445762
N	-5.429796	8.202655	7.859494

C	-6.660690	8.429626	8.317467
C	-7.428204	7.299174	8.974960
H	-7.017173	7.073472	9.965618
H	-8.481087	7.562664	9.093750
H	-7.354004	6.381331	8.383563
C	-4.799378	6.958218	8.190372
C	-4.271826	6.781255	9.490917
C	-3.658908	5.569766	9.802802
H	-3.258130	5.411944	10.801066
C	-3.539452	4.557464	8.855789
H	-3.057180	3.618528	9.118250
C	-4.033569	4.754944	7.576557
H	-3.930678	3.966225	6.833928
C	-4.675556	5.945857	7.221497
C	-4.327684	7.901528	10.524467
H	-5.185953	8.541403	10.295151
C	-3.076499	8.783723	10.424679
H	-2.955156	9.196045	9.414978
H	-3.138403	9.624887	11.126820
H	-2.173140	8.206976	10.658827
C	-4.512504	7.399941	11.960044
H	-3.624124	6.874169	12.329094
H	-4.688525	8.247110	12.632423
H	-5.365936	6.716922	12.042165
C	-5.241633	6.086454	5.815567
H	-5.659863	7.093996	5.713701
C	-6.390257	5.097367	5.577970
H	-7.197570	5.234659	6.306912
H	-6.811840	5.235247	4.575592
H	-6.041833	4.060276	5.656225
C	-4.153939	5.923677	4.748237
H	-3.744513	4.906300	4.741826
H	-4.561092	6.133894	3.752042
H	-3.318195	6.613635	4.918404
Pd	-3.040687	9.527484	4.475798
Mg	-4.565879	9.550455	6.602053
Mg	-0.530812	9.494526	5.215593
H	-3.546504	9.532308	3.002666
H	-1.623148	9.735575	3.655528

NiMgH4_freq.log

SCF (wB97x) = SCF
E(SCF)+ZPE(0 K)= -2651.918298
H(298 K)= -2651.841795
G(298 K)= -2652.035334
Lowest Frequency = 8.2485cm-1

H	-0.839116	9.790931	7.522164
H	-1.699091	9.730701	5.351546
N	1.478155	10.920647	5.291335
C	2.735377	10.613819	5.021614
C	3.251412	9.299789	5.044347
H	4.314816	9.216334	4.849816
C	3.703141	11.718733	4.656395
H	3.328577	12.285457	3.796981
H	4.690061	11.320328	4.413623
H	3.803970	12.429551	5.483835
C	0.997138	12.264279	5.172732
C	0.342248	12.652645	3.985569
C	-0.203282	13.935162	3.910703
H	-0.705794	14.248022	2.997385
C	-0.124233	14.814918	4.981546
H	-0.557161	15.809415	4.906473
C	0.503253	14.412251	6.152716
H	0.548579	15.097226	6.997044
C	1.068242	13.141817	6.274111
C	0.200488	11.716869	2.791493
H	0.646713	10.751793	3.060946
C	-1.274298	11.467458	2.448273
H	-1.821673	11.068304	3.310535
H	-1.359816	10.748296	1.624478
H	-1.771962	12.393551	2.134773
C	0.961363	12.246705	1.569592
H	0.555226	13.209792	1.237226
H	0.882378	11.543168	0.732364
H	2.025001	12.392243	1.791027
C	1.703819	12.736744	7.597370
H	2.151001	11.744307	7.469581
C	2.821436	13.699265	8.019362
H	3.588406	13.800664	7.242800
H	3.308475	13.339769	8.933133
H	2.427835	14.701273	8.227584
C	0.643559	12.630841	8.702881
H	0.175194	13.605224	8.888926
H	1.099881	12.293107	9.641367
H	-0.149420	11.923635	8.434480
N	1.265484	7.962308	5.425171
C	2.566711	8.075130	5.182964
C	3.394103	6.820175	4.995259
H	3.147424	6.338854	4.041466
H	4.461743	7.048801	4.998547
H	3.180965	6.090809	5.783240
C	0.631708	6.679109	5.360449
C	0.288442	6.143991	4.098882
C	-0.367227	4.914969	4.050229
H	-0.634057	4.487740	3.086295
C	-0.697128	4.228649	5.213549
H	-1.204136	3.267942	5.156149
C	-0.387175	4.782139	6.446396
H	-0.667197	4.254794	7.356049
C	0.272507	6.010174	6.545976
C	0.552979	6.916000	2.811869
H	1.337823	7.654366	3.006996
C	-0.706648	7.691070	2.398856
H	-1.049698	8.362319	3.196587
H	-0.516122	8.295071	1.502512
H	-1.530493	7.000628	2.177909
C	1.041411	6.028910	1.662156
H	0.261580	5.340846	1.315549

H	1.330248	6.648576	0.805664
H	1.910328	5.429131	1.957225
C	0.582106	6.573037	7.926165
H	1.014677	7.571904	7.795211
C	1.629484	5.718832	8.651987
H	2.560096	5.649514	8.076528
H	1.867095	6.151136	9.631018
H	1.260712	4.698812	8.815520
C	-0.687247	6.721179	8.775404
H	-1.138493	5.747697	9.002317
H	-0.452810	7.213859	9.727092
H	-1.443218	7.327401	8.261780
H	-3.542405	9.819159	6.029336
H	-2.745016	9.890424	8.242906
N	-6.026442	11.016601	8.019672
C	-7.305115	10.669235	8.013498
C	-7.769798	9.342957	7.931008
H	-8.848443	9.231481	7.925039
C	-8.363246	11.744424	8.141896
H	-8.389095	12.130709	9.167977
H	-9.353038	11.352390	7.898096
H	-8.141288	12.592959	7.487285
C	-5.645261	12.369673	8.292918
C	-5.605980	12.813670	9.632982
C	-5.172740	14.112261	9.895055
H	-5.133837	14.467243	10.922378
C	-4.779171	14.960731	8.867062
H	-4.446571	15.971500	9.089817
C	-4.801863	14.505332	7.557838
H	-4.481816	15.167227	6.755775
C	-5.222921	13.210084	7.244973
C	-5.953574	11.884382	10.789544
H	-6.532641	11.043031	10.394671
C	-4.670803	11.303568	11.400712
H	-4.065554	10.781665	10.648631
H	-4.908060	10.591758	12.201323
H	-4.048061	12.100415	11.825577
C	-6.809044	12.558306	11.867462
H	-6.256139	13.341844	12.398436
H	-7.124667	11.820706	12.614098
H	-7.708462	13.015937	11.439127
C	-5.218810	12.765469	5.788543
H	-5.481216	11.701715	5.756911
C	-6.281996	13.519274	4.978660
H	-7.284472	13.372898	5.397656
H	-6.294164	13.169038	3.939886
H	-6.078560	14.597113	4.968195
C	-3.833477	12.920476	5.146640
H	-3.525272	13.972068	5.094221
H	-3.843342	12.524742	4.123752
H	-3.066280	12.372629	5.707114
N	-5.719291	8.052846	7.917455
C	-7.041786	8.133255	7.963353
C	-7.855849	6.863143	8.091739
H	-7.693506	6.414657	9.079144
H	-8.922792	7.062226	7.974144
H	-7.545730	6.118496	7.352495
C	-5.069565	6.779957	8.032163
C	-4.737213	6.286607	9.310607
C	-4.077766	5.060744	9.405050
H	-3.819653	4.667724	10.386547
C	-3.733788	4.339938	8.268950
H	-3.224451	3.383136	8.360905
C	-4.028631	4.856803	7.015251
H	-3.730184	4.305784	6.125684
C	-4.689809	6.078146	6.870116
C	-5.018290	7.082138	10.578391
H	-5.651198	7.936719	10.312362
C	-3.705237	7.636062	11.150708
H	-3.175677	8.254486	10.414703
H	-3.894096	8.248918	12.040564
H	-3.030841	6.819445	11.437640
C	-5.770122	6.264753	11.634826
H	-5.167530	5.425398	12.001488
H	-6.016777	6.893748	12.498006
H	-6.704732	5.853987	11.235606
C	-4.964840	6.607566	5.469029
H	-5.427325	7.596506	5.565620
C	-5.949614	5.709486	4.708922
H	-6.904791	5.613823	5.238038
H	-6.154799	6.122611	3.714431
H	-5.540096	4.700881	4.574304
C	-3.664554	6.777953	4.671953
H	-3.172857	5.814589	4.488826
H	-3.873405	7.240841	3.699218
H	-2.953729	7.421634	5.203021
Mg	-4.563861	9.688865	7.658142
Mg	0.111585	9.562948	5.864973
Ni	-2.200135	9.813883	6.784363

NiZnH4_freq.log

SCF (wB97x) = SCF
E(SCF)+ZPE(0 K)= -3104.462603
H(298 K)= -3104.386239
G(298 K)= -3104.575921
Lowest Frequency = 14.9819cm-1

Zn	-0.092825	9.521679	5.875417
H	-0.945108	9.521689	7.452664
H	-1.755956	9.521694	5.262722
N	1.126911	11.007204	5.496385
C	2.423221	10.796219	5.322788
C	3.026541	9.521652	5.304695
H	4.103451	9.521643	5.179232
C	3.334974	11.981967	5.092395
H	3.163102	12.401535	4.094080
H	4.384658	11.690473	5.166324
H	3.131838	12.780265	5.812678
C	0.559298	12.317460	5.379680

C	0.234892	12.818363	4.102045
C	-0.378053	14.068261	4.010922
H	-0.635318	14.466381	3.031471
C	-0.680371	14.806502	5.147945
H	-1.156969	15.780020	5.057572
C	-0.381263	14.288649	6.400079
H	-0.638629	14.858736	7.290430
C	0.233520	13.042645	6.542651
C	0.467903	12.006343	2.834685
H	1.105312	11.151511	3.085336
C	-0.864930	11.446844	2.317191
H	-1.363028	10.837321	3.080778
H	-0.706955	10.823218	1.428471
H	-1.548827	12.260732	2.044790
C	1.181575	12.807616	1.739975
H	0.560879	13.632502	1.370975
H	1.408876	12.161502	0.884263
H	2.123149	13.236860	2.102025
C	0.529263	12.516368	7.940031
H	0.881844	11.483858	7.843204
C	1.646933	13.324988	8.611889
H	2.572871	13.299153	8.025419
H	1.866798	12.923563	9.608150
H	1.355193	14.375938	8.729505
C	-0.729084	12.497151	8.817434
H	-1.091465	13.510056	9.031728
H	-0.515344	12.008753	9.776413
H	-1.544941	11.948184	8.333064
N	1.126885	8.036134	5.496386
C	2.423199	8.247096	5.322789
C	3.334931	7.061332	5.092396
H	3.163050	6.641764	4.094084
H	4.384621	7.352809	5.166322
H	3.131784	6.263040	5.812683
C	0.559249	6.725888	5.379681
C	0.234834	6.224991	4.102046
C	-0.378133	4.975104	4.010923
H	-0.635406	4.576990	3.031471
C	-0.680464	4.236868	5.147946
H	-1.157079	3.263359	5.057572
C	-0.381345	4.754715	6.400079
H	-0.638721	4.184632	7.290431
C	0.233459	6.000708	6.542652
C	0.467859	7.037008	2.834687
H	1.105284	7.891828	3.085339
C	-0.864964	7.596533	2.317195
H	-1.363050	8.206065	3.080783
H	-0.706979	8.220157	1.428476
H	-1.548876	6.782658	2.044794
C	1.181514	6.235723	1.739976
H	0.560803	5.410850	1.370974
H	1.408827	6.881833	0.884264
H	2.123080	5.806460	2.102023
C	0.529213	6.526978	7.940033
H	0.881816	7.559480	7.843207

C	1.646867	5.718334	8.611889
H	2.572805	5.744151	8.025418
H	1.866743	6.119755	9.608150
H	1.355107	4.667391	8.729505
C	-0.729133	6.546220	8.817437
H	-1.091535	5.533322	9.031729
H	-0.515381	7.034611	9.776417
H	-1.544978	7.095205	8.333068
Zn	-4.457051	9.521720	7.536534
H	-3.604767	9.521712	5.959287
H	-2.793921	9.521706	8.149229
N	-5.676761	11.007265	7.915566
C	-6.973075	10.796303	8.089165
C	-7.576416	9.521746	8.107258
H	-8.653326	9.521755	8.232723
C	-7.884807	11.982066	8.319559
H	-7.712924	12.401636	9.317871
H	-8.934497	11.690589	8.245635
H	-7.681661	12.780359	7.599271
C	-5.109126	12.317511	8.032272
C	-4.784711	12.818408	9.309907
C	-4.171746	14.068296	9.401031
H	-3.914474	14.466411	10.380482
C	-3.869418	14.806534	8.264008
H	-3.392805	15.780044	8.354382
C	-4.168536	14.288687	7.011874
H	-3.911163	14.858772	6.121523
C	-4.783338	13.042693	6.869301
C	-5.017734	12.006391	10.577267
H	-5.655153	11.151567	10.326614
C	-3.684909	11.446874	11.094762
H	-3.186818	10.837344	10.331176
H	-3.842893	10.823251	11.983482
H	-3.001001	12.260754	11.367163
C	-5.731396	12.807674	11.671975
H	-5.110690	13.632551	12.040976
H	-5.958707	12.161563	12.527688
H	-6.672964	13.236930	11.309925
C	-5.079092	12.516423	5.471920
H	-5.431693	11.483921	5.568746
C	-6.196747	13.325066	4.800064
H	-7.122685	13.299248	5.386535
H	-6.416622	12.923645	3.803804
H	-5.904988	14.376010	4.682448
C	-3.820746	12.497183	4.594516
H	-3.458345	13.510083	4.380226
H	-4.034496	12.008794	3.635536
H	-3.004900	11.948199	5.078885
N	-5.676786	8.036195	7.915566
C	-6.973097	8.247180	8.089165
C	-7.884848	7.061432	8.319559
H	-7.712976	6.641863	9.317873
H	-8.934533	7.352926	8.245632
H	-7.681713	6.263134	7.599274
C	-5.109172	6.725940	8.032273

C	-4.784765	6.225039	9.309909
C	-4.171821	4.975141	9.401034
H	-3.914554	4.577023	10.380485
C	-3.869505	4.236897	8.264011
H	-3.392908	3.263380	8.354386
C	-4.168615	4.754748	7.011877
H	-3.911252	4.184659	6.121527
C	-4.783397	6.000752	6.869304
C	-5.017774	7.037062	10.577268
H	-5.655179	7.891896	10.326616
C	-3.684939	7.596557	11.094762
H	-3.186839	8.206078	10.331175
H	-3.842912	8.220183	11.983482
H	-3.001044	6.782666	11.367163
C	-5.731448	6.235791	11.671978
H	-5.110755	5.410904	12.040979
H	-5.958747	6.881907	12.527690
H	-6.673023	5.806550	11.309930
C	-5.079143	6.527026	5.471922
H	-5.431724	7.559536	5.568748
C	-6.196814	5.718404	4.800069
H	-7.122751	5.744241	5.386541
H	-6.416683	6.119828	3.803808
H	-5.905076	4.667454	4.682455
C	-3.820797	6.546241	4.594517
H	-3.458418	5.533334	4.380226
H	-4.034540	7.034635	3.635537
H	-3.004940	7.095208	5.078884
Ni	-2.274938	9.521700	6.705975

PdMgH4_freq.log

SCF (wB97x) = SCF
E(SCF)+ZPE(0 K)= -2608.916332
H(298 K)= -2608.839707
G(298 K)= -2609.033884
Lowest Frequency = 9.2577cm-1

H	-0.884377	9.850518	7.430983
H	-1.680992	9.887862	5.088394
N	1.641707	10.961322	5.417623
C	2.907681	10.575708	5.320861
C	3.335714	9.232630	5.340418
H	4.407603	9.088597	5.264086
C	3.987547	11.620810	5.133093
H	3.923856	12.053269	4.127312
H	4.981750	11.187170	5.257949
H	3.864239	12.446132	5.840892
C	1.295873	12.342061	5.250540
C	1.130484	12.857076	3.946848
C	0.730015	14.184144	3.798516
H	0.594694	14.594441	2.800233
C	0.493833	14.992257	4.904047
H	0.187138	16.026512	4.769489
C	0.642212	14.468325	6.179695
H	0.446324	15.100486	7.043342
C	1.032423	13.141776	6.379501
C	1.319071	11.981494	2.714018
H	1.872220	11.084305	3.011963
C	-0.042977	11.519359	2.177165
H	-0.621678	10.990016	2.944576
H	0.083992	10.845968	1.320259
H	-0.642814	12.377708	1.850362
C	2.129414	12.670202	1.610473
H	1.590722	13.522139	1.179610
H	2.331022	11.966515	0.794739
H	3.090411	13.039288	1.987404
C	1.165546	12.614296	7.801986
H	1.360950	11.537117	7.745941
C	2.356536	13.257238	8.524779
H	3.297742	13.073427	7.993925
H	2.455565	12.852059	9.538669
H	2.226183	14.343249	8.607779
C	-0.126281	12.810579	8.606655
H	-0.339634	13.873895	8.771003
H	-0.036156	12.333710	9.590380
H	-0.987345	12.366192	8.094866
N	1.248677	8.007987	5.465264
C	2.569337	8.048214	5.345379
C	3.336346	6.755400	5.160865
H	3.125893	6.333591	4.170802
H	4.412569	6.919291	5.243419
H	3.030077	6.004467	5.895168
C	0.561920	6.752986	5.368789
C	0.185500	6.265863	4.099721
C	-0.502689	5.054806	4.024933
H	-0.795400	4.666062	3.051541
C	-0.828892	4.341204	5.171004
H	-1.358040	3.393851	5.093773
C	-0.489139	4.851988	6.415660
H	-0.771050	4.305815	7.313432
C	0.198350	6.060933	6.541526
C	0.456858	7.052077	2.823600
H	1.137176	7.875878	3.067974
C	-0.847140	7.669865	2.297654
H	-1.324506	8.306213	3.053376
H	-0.657579	8.280220	1.405902
H	-1.566871	6.886868	2.027015
C	1.131408	6.205497	1.738222
H	0.474375	5.402355	1.384324
H	1.383016	6.828484	0.872195
H	2.055367	5.742600	2.103802
C	0.528101	6.584002	7.933363
H	0.978021	7.577423	7.822675
C	1.556144	5.691094	8.640499
H	2.489245	5.616382	8.070289
H	1.797595	6.094415	9.630924
H	1.165858	4.675194	8.777992
C	-0.734979	6.737018	8.791285
H	-1.201914	5.766900	9.000514

H	-0.486182	7.204756	9.752275
H	-1.482255	7.366118	8.293980
H	-3.767763	9.846008	5.823427
H	-2.927632	9.820857	8.149467
N	-6.197340	10.928264	8.027509
C	-7.443645	10.562243	8.292195
C	-7.894790	9.225879	8.292221
H	-8.953693	9.094373	8.484605
C	-8.471420	11.622998	8.624501
H	-8.124476	12.248890	9.453423
H	-9.429980	11.176391	8.895357
H	-8.624537	12.289285	7.768356
C	-5.800838	12.300651	8.142666
C	-5.158698	12.726527	9.323798
C	-4.719367	14.048936	9.405806
H	-4.232440	14.393341	10.316084
C	-4.886645	14.930134	8.346516
H	-4.536610	15.956182	8.427442
C	-5.494599	14.489461	7.178458
H	-5.606760	15.177535	6.343052
C	-5.958036	13.179479	7.051612
C	-4.932917	11.794521	10.508211
H	-5.255745	10.787914	10.215279
C	-3.449634	11.711881	10.892390
H	-2.833383	11.403099	10.039821
H	-3.306124	10.982398	11.698995
H	-3.072713	12.678472	11.248194
C	-5.784737	12.210202	11.714444
H	-5.511464	13.213700	12.063013
H	-5.637811	11.513259	12.548128
H	-6.852469	12.221847	11.466777
C	-6.572692	12.730965	5.732431
H	-6.991237	11.728416	5.875749
C	-7.713921	13.648762	5.276658
H	-8.485419	13.754692	6.048176
H	-8.187985	13.245346	4.374527
H	-7.348923	14.653703	5.033968
C	-5.496590	12.632157	4.641709
H	-5.050052	13.614923	4.446032
H	-5.930749	12.265734	3.703427
H	-4.688231	11.951966	4.933588
N	-5.846005	7.979067	7.942591
C	-7.150594	8.033332	8.183887
C	-7.916302	6.746503	8.413345
H	-7.658472	6.321148	9.390630
H	-8.993822	6.921396	8.389542
H	-7.657651	5.994391	7.661778
C	-5.159583	6.724079	8.032145
C	-4.777072	6.237398	9.301361
C	-4.084860	5.029362	9.372462
H	-3.786899	4.639326	10.343248
C	-3.760698	4.317132	8.223802
H	-3.227960	3.371640	8.299002
C	-4.108867	4.825372	6.981338
H	-3.832264	4.278472	6.082427
C	-4.801731	6.032437	6.858919
C	-5.047069	7.030291	10.574672
H	-5.822407	7.773532	10.360010
C	-3.785134	7.797526	10.996036
H	-3.428831	8.461305	10.198063
H	-3.980722	8.408033	11.887038
H	-2.968960	7.102219	11.230504
C	-5.559317	6.159955	11.727386
H	-4.793017	5.462169	12.084594
H	-5.845948	6.790149	12.576961
H	-6.434710	5.570920	11.429695
C	-5.152721	6.547664	5.469805
H	-5.583214	7.549929	5.579992
C	-6.218403	5.667974	4.803244
H	-7.135162	5.621786	5.402559
H	-6.479939	6.062780	3.814465
H	-5.853330	4.642151	4.670160
C	-3.910503	6.669063	4.577589
H	-3.469905	5.688421	4.360859
H	-4.174702	7.137049	3.621117
H	-3.136305	7.284158	5.051340
Pd	-2.316856	9.853936	6.619032
Mg	-4.750903	9.621466	7.494778
Mg	0.131795	9.666101	5.778615

Pd-Mg.log

SCF (wB97x) = -2610.24441992
E(SCF)+ZPE(0 K)= -2608.916332
H(298 K)= -2608.839707
G(298 K)= -2609.033884
Lowest Frequency = 9.2581cm-1

H	-0.884373	9.850516	7.430983
H	-1.680993	9.887847	5.088398
N	1.641706	10.961322	5.417618
C	2.907680	10.575709	5.320855
C	3.335715	9.232631	5.340411
H	4.407604	9.088599	5.264079
C	3.987545	11.620812	5.133085
H	3.923852	12.053272	4.127304
H	4.981748	11.187172	5.257938
H	3.864237	12.446134	5.840884
C	1.295871	12.342061	5.250536
C	1.130476	12.857076	3.946845
C	0.730006	14.184143	3.798513
H	0.594681	14.594441	2.800232
C	0.493829	14.992257	4.904046
H	0.187133	16.026511	4.769488
C	0.642214	14.468325	6.179693
H	0.446329	15.100487	7.043340
C	1.032426	13.141777	6.379498
C	1.319056	11.981494	2.714014
H	1.872206	11.084303	3.011956
C	-0.042995	11.519362	2.177167

H	-0.621694	10.990019	2.944579
H	0.083968	10.845971	1.320259
H	-0.642832	12.377712	1.850368
C	2.129395	12.670200	1.610465
H	1.590704	13.522138	1.179605
H	2.330998	11.966512	0.794729
H	3.090396	13.039282	1.987392
C	1.165557	12.614298	7.801983
H	1.360959	11.537118	7.745938
C	2.356553	13.257238	8.524767
H	3.297756	13.073425	7.993907
H	2.455588	12.852062	9.538657
H	2.226203	14.343249	8.607766
C	-0.126263	12.810585	8.606661
H	-0.339611	13.873901	8.771010
H	-0.036131	12.333717	9.590387
H	-0.987332	12.366200	8.094880
N	1.248680	8.007987	5.465263
C	2.569339	8.048214	5.345375
C	3.336349	6.755401	5.160861
H	3.125902	6.333596	4.170795
H	4.412571	6.919292	5.243424
H	3.030075	6.004465	5.895159
C	0.561922	6.752987	5.368788
C	0.185506	6.265861	4.099721
C	-0.502684	5.054804	4.024933
H	-0.795392	4.666057	3.051541
C	-0.828892	4.341206	5.171006
H	-1.358041	3.393853	5.093775
C	-0.489143	4.851993	6.415661
H	-0.771059	4.305823	7.313434
C	0.198347	6.060938	6.541525
C	0.456868	7.052071	2.823597
H	1.137187	7.875870	3.067971
C	-0.847128	7.669860	2.297648
H	-1.324494	8.306212	3.053367
H	-0.657565	8.280211	1.405894
H	-1.566860	6.886863	2.027011
C	1.131418	6.205487	1.738222
H	0.474384	5.402346	1.384324
H	1.383028	6.828473	0.872195
H	2.055375	5.742589	2.103804
C	0.528096	6.584010	7.933362
H	0.978014	7.577431	7.822673
C	1.556141	5.691105	8.640500
H	2.489244	5.616396	8.070290
H	1.797592	6.094428	9.630924
H	1.165859	4.675205	8.777993
C	-0.734984	6.737025	8.791285
H	-1.201916	5.766906	9.000517
H	-0.486187	7.204765	9.752272
H	-1.482261	7.366122	8.293978
H	-3.767762	9.846002	5.823433
H	-2.927630	9.820860	8.149472
N	-6.197340	10.928263	8.027513
C	-7.443646	10.562242	8.292195
C	-7.894791	9.225878	8.292222
H	-8.953694	9.094372	8.484604
C	-8.471422	11.622997	8.624497
H	-8.124482	12.248888	9.453423
H	-9.429984	11.176392	8.895345
H	-8.624531	12.289287	7.768353
C	-5.800839	12.300650	8.142671
C	-5.158704	12.726527	9.323805
C	-4.719372	14.048935	9.405814
H	-4.232447	14.393341	10.316092
C	-4.886642	14.930132	8.346520
H	-4.536605	15.956178	8.427447
C	-5.494591	14.489459	7.178461
H	-5.606745	15.177531	6.343052
C	-5.958030	13.179477	7.051615
C	-4.932930	11.794521	10.508219
H	-5.255758	10.787915	10.215286
C	-3.449649	11.711878	10.892404
H	-2.833394	11.403098	10.039837
H	-3.306142	10.982393	11.699008
H	-3.072728	12.678469	11.248212
C	-5.784754	12.210203	11.714449
H	-5.511482	13.213701	12.063018
H	-5.637830	11.513262	12.548134
H	-6.852485	12.221847	11.466779
C	-6.572682	12.730963	5.732430
H	-6.991231	11.728415	5.875749
C	-7.713905	13.648763	5.276649
H	-8.485408	13.754695	6.048161
H	-8.187962	13.245348	4.374515
H	-7.348901	14.653702	5.033962
C	-5.496573	12.632149	4.641716
H	-5.050030	13.614914	4.446041
H	-5.930728	12.265728	3.703431
H	-4.688219	11.951955	4.933599
N	-5.846005	7.979068	7.942597
C	-7.150594	8.033332	8.183889
C	-7.916303	6.746503	8.413346
H	-7.658483	6.321153	9.390636
H	-8.993822	6.921394	8.389530
H	-7.657643	5.994387	7.661785
C	-5.159583	6.724080	8.032148
C	-4.777076	6.237394	9.301363
C	-4.084864	5.029358	9.372462
H	-3.786908	4.639318	10.343247
C	-3.760697	4.317133	8.223800
H	-3.227959	3.371641	8.298998
C	-4.108861	4.825379	6.981337
H	-3.832254	4.278482	6.082424
C	-4.801726	6.032443	6.858920
C	-5.047079	7.030282	10.574676
H	-5.822418	7.773522	10.360014
C	-3.785147	7.797518	10.996046
H	-3.428844	8.461302	10.198077

H	-3.980737	8.408020	11.887050
H	-2.968971	7.102212	11.230512
C	-5.559328	6.159940	11.727385
H	-4.793028	5.462155	12.084593
H	-5.845965	6.790131	12.576962
H	-6.434720	5.570903	11.429690
C	-5.152712	6.547674	5.469806
H	-5.583200	7.549941	5.579994
C	-6.218401	5.667991	4.803245
H	-7.135161	5.621811	5.402561
H	-6.479935	6.062799	3.814467
H	-5.853336	4.642166	4.670161
C	-3.910496	6.669066	4.577589
H	-3.469905	5.688422	4.360855
H	-4.174693	7.137055	3.621118
H	-3.136293	7.284154	5.051339
Pd	-2.316855	9.853931	6.619036
Mg	-4.750900	9.621466	7.494784
Mg	0.131796	9.666101	5.778614

PdZnH4_freq.log

SCF (wB97x) = SCF
E(SCF)+ZPE(0 K)= -3061.444828
H(298 K)= -3061.367970
G(298 K)= -3061.560482
Lowest Frequency = 12.8293cm-1

Zn	0.024982	9.521701	5.831164
H	-0.810858	9.521700	7.470059
H	-1.665012	9.521700	5.163462
N	1.231888	11.011128	5.457427
C	2.526647	10.797924	5.273798
C	3.128062	9.521699	5.253269
H	4.204114	9.521698	5.120135
C	3.436977	11.981988	5.031146
H	3.270993	12.383819	4.024542
H	4.486899	11.694440	5.116661
H	3.226610	12.791604	5.736407
C	0.652927	12.316389	5.334320
C	0.321784	12.806354	4.054086
C	-0.308664	14.047084	3.955886
H	-0.571670	14.435546	2.974095
C	-0.621096	14.787525	5.088354
H	-1.110213	15.754192	4.992600
C	-0.316799	14.279574	6.343276
H	-0.582391	14.851160	7.230033
C	0.312086	13.041560	6.493616
C	0.560295	11.992118	2.789207
H	1.190769	11.133347	3.044265
C	-0.772224	11.441587	2.261095
H	-1.281793	10.837430	3.021327
H	-0.611102	10.815328	1.374827
H	-1.447590	12.259867	1.981066
C	1.286587	12.790116	1.700210
H	0.674203	13.621118	1.331050
H	1.513098	12.144659	0.843783
H	2.229600	13.211114	2.068054
C	0.605546	12.524991	7.895321
H	0.929991	11.482378	7.808610
C	1.750998	13.312135	8.545631
H	2.671605	13.249072	7.953619
H	1.966445	12.922100	9.547406
H	1.489791	14.372938	8.645807
C	-0.643465	12.548735	8.786773
H	-0.968793	13.573212	9.005920
H	-0.433651	12.054756	9.743645
H	-1.482453	12.025833	8.313354
N	1.231886	8.032272	5.457429
C	2.526646	8.245475	5.273799
C	3.436974	7.061410	5.031147
H	3.270991	6.659580	4.024542
H	4.486896	7.348956	5.116665
H	3.226605	6.251792	5.736406
C	0.652923	6.727012	5.334323
C	0.321774	6.237047	4.054091
C	-0.308677	4.996319	3.955893
H	-0.571687	4.607858	2.974103
C	-0.621106	4.255879	5.088362
H	-1.110226	3.289214	4.992610
C	-0.316805	4.763830	6.343283
H	-0.582395	4.192245	7.230040
C	0.312084	6.001842	6.493620
C	0.560281	7.051283	2.789210
H	1.190760	7.910052	3.044265
C	-0.772238	7.601819	2.261105
H	-1.281801	8.205978	3.021340
H	-0.611118	8.228078	1.374836
H	-1.447609	6.783542	1.981079
C	1.286565	6.253282	1.700209
H	0.674176	5.422284	1.331052
H	1.513074	6.898739	0.843781
H	2.229578	5.832281	2.068048
C	0.605548	6.518413	7.895324
H	0.930000	7.561023	7.808610
C	1.750996	5.731262	8.545635
H	2.671603	5.794318	7.953621
H	1.966447	6.121299	9.547407
H	1.489783	4.670462	8.645813
C	-0.643462	6.494678	8.786777
H	-0.968797	5.470203	9.005926
H	-0.433644	6.988658	9.743649
H	-1.482447	7.017585	8.313359
Zn	-4.574861	9.521699	7.580784
H	-3.739021	9.521699	5.941891
H	-2.884866	9.521698	8.248489
N	-5.781766	11.011127	7.954523
C	-7.076525	10.797923	8.138153
C	-7.677940	9.521699	8.158683
H	-8.753992	9.521699	8.291818

C	-7.986854	11.981988	8.380807
H	-7.820868	12.383817	9.387412
H	-9.036775	11.694441	8.295293
H	-7.776486	12.791605	7.675548
C	-5.202803	12.316388	8.077631
C	-4.871664	12.806353	9.357866
C	-4.241215	14.047082	9.456068
H	-3.978214	14.435544	10.437860
C	-3.928778	14.787521	8.323600
H	-3.439660	15.754187	8.419356
C	-4.233069	14.279570	7.068678
H	-3.967472	14.851154	6.181922
C	-4.861956	13.041557	6.918336
C	-5.110183	11.992118	10.622745
H	-5.740657	11.133347	10.367684
C	-3.777667	11.441585	11.150863
H	-3.268094	10.837428	10.390633
H	-3.938795	10.815324	12.037129
H	-3.102301	12.259863	11.430898
C	-5.836479	12.790117	11.711737
H	-5.224094	13.621117	12.080901
H	-6.062997	12.144661	12.568163
H	-6.779489	13.211118	11.343888
C	-5.155408	12.524985	5.516630
H	-5.479860	11.482375	5.603342
C	-6.300849	13.312135	4.866307
H	-7.221461	13.249081	5.458312
H	-6.516289	12.922098	3.864533
H	-6.039633	14.372936	4.766130
C	-3.906388	12.548718	4.625189
H	-3.581051	13.573192	4.406043
H	-4.116197	12.054738	3.668316
H	-3.067408	12.025811	5.098617
N	-5.781765	8.032271	7.954524
C	-7.076524	8.245475	8.138153
C	-7.986853	7.061410	8.380808
H	-7.820866	6.659579	9.387412
H	-9.036774	7.348956	8.295294
H	-7.776486	6.251793	7.675548
C	-5.202802	6.727011	8.077630
C	-4.871659	6.237047	9.357865
C	-4.241209	4.996319	9.456066
H	-3.978204	4.607857	10.437858
C	-3.928775	4.255878	8.323598
H	-3.439656	3.289212	8.419353
C	-4.233070	4.763829	7.068676
H	-3.967475	4.192243	6.181920
C	-4.861957	6.001841	6.918335
C	-5.110174	7.051283	10.622744
H	-5.740648	7.910053	10.367684
C	-3.777657	7.601815	11.150859
H	-3.268085	8.205971	10.390627
H	-3.938782	8.228076	12.037125
H	-3.102291	6.783536	11.430892
C	-5.836467	6.253284	11.711738
H	-5.224083	5.422284	12.080900
H	-6.062983	6.898741	12.568165
H	-6.779479	5.832284	11.343892
C	-5.155413	6.518411	5.516629
H	-5.479863	7.561022	5.603340
C	-6.300859	5.731263	4.866313
H	-7.221469	5.794321	5.458321
H	-6.516304	6.121299	3.864539
H	-6.039647	4.670461	4.766136
C	-3.906398	6.494673	4.625183
H	-3.581064	5.470197	4.406037
H	-4.116210	6.988651	3.668310
H	-3.067415	7.017578	5.098606
Pd	-2.274939	9.521701	6.705975

PtMgH4_freq.log

SCF (wB97x) = SCF
E(SCF)+ZPE(0 K)= -2600.427474
H(298 K)= -2600.350837
G(298 K)= -2600.544987
Lowest Frequency = 9.5795cm-1

H	-0.784935	9.905955	7.595813
H	-1.640294	9.875296	5.238676
N	1.640341	10.941391	5.363927
C	2.886540	10.575413	5.099258
C	3.338846	9.239089	5.104354
H	4.397917	9.108171	4.912744
C	3.913700	11.635151	4.762030
H	3.563518	12.261867	3.935173
H	4.870421	11.187414	4.486627
H	4.071637	12.300775	5.617781
C	1.243424	12.313475	5.242336
C	0.598931	12.732545	4.060254
C	0.165001	14.056147	3.969181
H	-0.323302	14.395552	3.057802
C	0.338672	14.944583	5.021338
H	-0.007475	15.971380	4.933576
C	0.946261	14.509780	6.191686
H	1.061543	15.203004	7.022391
C	1.404566	13.198877	6.327264
C	0.363277	11.791466	2.885071
H	0.674095	10.784090	3.188451
C	-1.120587	11.722509	2.500483
H	-1.741864	11.437707	3.357754
H	-1.273282	10.981499	1.706186
H	-1.483838	12.687803	2.127507
C	1.221331	12.183780	1.675502
H	0.962252	13.187860	1.317875
H	1.064929	11.481362	0.848120
H	2.289009	12.182508	1.923690
C	2.014726	12.756333	7.650408
H	2.425837	11.749485	7.515491
C	3.162222	13.668802	8.101043

H	3.937025	13.761407	7.331140	H	-5.177601	14.562772	10.672008
H	3.630217	13.270014	9.008399	C	-5.073050	14.979776	8.572180
H	2.804565	14.678860	8.333166	H	-4.773425	16.014737	8.716867
C	0.935733	12.675124	8.739899	C	-5.214116	14.466342	7.291510
H	0.504116	13.664998	8.933233	H	-5.019550	15.107313	6.434141
H	1.363034	12.304026	9.679532	C	-5.594648	13.139002	7.078581
H	0.116770	12.008012	8.447371	C	-5.883237	11.943405	10.732338
N	1.291185	7.989991	5.457228	H	-6.438481	11.049961	10.427100
C	2.596541	8.046262	5.217236	C	-4.519628	11.474081	11.258738
C	3.364404	6.759261	4.996828	H	-3.944724	10.952328	10.483310
H	3.095179	6.317293	4.030118	H	-4.643531	10.791881	12.109104
H	4.441439	6.938165	5.004542	H	-3.917192	12.328293	11.591381
H	3.117909	6.018342	5.763640	C	-6.688528	12.620272	11.846779
C	0.609355	6.731839	5.371103	H	-6.146561	13.465353	12.286875
C	0.225543	6.243223	4.103108	H	-6.889449	11.906869	12.654191
C	-0.461924	5.032392	4.034221	H	-7.649690	12.996290	11.477204
H	-0.760803	4.640576	3.064475	C	-5.721047	12.623843	5.651063
C	-0.780297	4.319584	5.184229	H	-5.903468	11.543833	5.696750
H	-1.310204	3.372352	5.110959	C	-6.920674	13.258503	4.935170
C	-0.429582	4.829065	6.425427	H	-7.859382	13.056742	5.463997
H	-0.701521	4.281618	7.325477	H	-7.014439	12.862765	3.917040
C	0.259877	6.038327	6.545336	H	-6.804471	14.346934	4.863661
C	0.490013	7.037649	2.829630	C	-4.432016	12.843638	4.848047
H	1.270559	7.776598	3.040367	H	-4.233200	13.911016	4.691938
C	-0.771404	7.811715	2.419440	H	-4.516907	12.373968	3.860287
H	-1.121003	8.473066	3.222474	H	-3.565365	12.405896	5.356457
H	-0.579190	8.425594	1.530030	N	-5.783484	7.993459	7.950448
H	-1.591719	7.120592	2.187338	C	-7.104780	8.027139	8.071743
C	0.989841	6.168352	1.670820	C	-7.864932	6.728932	8.245771
H	0.217703	5.475569	1.316492	H	-7.638799	6.290449	9.225009
H	1.273886	6.799813	0.821325	H	-8.942684	6.890259	8.180608
H	1.864340	5.574261	1.960939	H	-7.566226	5.992112	7.494083
C	0.613086	6.554536	7.933373	C	-5.093338	6.739355	8.039952
H	1.065737	7.546749	7.819746	C	-4.710062	6.250522	9.306110
C	1.655240	5.657052	8.613210	C	-4.021166	5.039516	9.375457
H	2.573209	5.583307	8.018414	H	-3.723141	4.649256	10.346615
H	1.921522	6.055828	9.599098	C	-3.700592	4.328165	8.226387
H	1.266417	4.641328	8.755639	H	-3.170326	3.381085	8.299151
C	-0.634065	6.707202	8.814043	C	-4.047166	4.840934	6.984400
H	-1.103487	5.737886	9.021469	H	-3.768943	4.297018	6.084079
H	-0.366791	7.163718	9.775380	C	-4.736395	6.049394	6.864295
H	-1.385336	7.345463	8.333636	C	-4.973908	7.036008	10.584198
H	-3.674145	9.898995	5.964650	H	-5.659702	7.856927	10.345383
H	-2.847321	9.946509	8.331606	C	-3.667459	7.658949	11.097783
N	-6.193313	10.946237	8.019490	H	-3.198252	8.295258	10.336718
C	-7.456888	10.552697	8.115011	H	-3.850647	8.270151	11.990228
C	-7.877216	9.207159	8.085458	H	-2.942723	6.878706	11.362748
H	-8.948145	9.056751	8.162384	C	-5.635860	6.187865	11.675959
C	-8.542709	11.589635	8.313096	H	-4.973292	5.386972	12.024538
H	-8.482453	12.011249	9.323692	H	-5.881832	6.810532	12.543835
H	-9.534246	11.151599	8.182569	H	-6.561594	5.722115	11.318578
H	-8.423387	12.423391	7.614635	C	-5.070948	6.575904	5.475075
C	-5.855671	12.327688	8.199600	H	-5.542741	7.558587	5.591148
C	-5.697848	12.831979	9.508320	C	-6.075588	5.667269	4.754642
C	-5.307025	14.160381	9.669776	H	-7.008531	5.564136	5.320793

H	-6.323076	6.076181	3.768028
H	-5.661719	4.662337	4.606403
C	-3.805142	6.762588	4.627620
H	-3.313142	5.804274	4.420990
H	-4.057527	7.225952	3.665301
H	-3.078355	7.408628	5.133925
Mg	-4.681740	9.659712	7.641836
Mg	0.199520	9.632354	5.901132
Pt	-2.235192	9.899547	6.785188

PtZnH4_freq.log

SCF (wB97x) = SCF
E(SCF)+ZPE(0 K)= -3052.953102
H(298 K)= -3052.875882
G(298 K)= -3053.071072
Lowest Frequency = 9.0806cm-1

Pt	-2.274938	9.521702	6.705977
Zn	0.039736	9.521700	5.834758
H	-0.818464	9.521704	7.504396
H	-1.684600	9.521702	5.148847
N	1.239240	11.012579	5.459894
C	2.533686	10.798263	5.273535
C	3.134496	9.521706	5.252127
H	4.210301	9.521708	5.117164
C	3.443977	11.981846	5.029029
H	3.275391	12.384329	4.023143
H	4.493935	11.693621	5.111577
H	3.235787	12.791110	5.735327
C	0.660653	12.318325	5.335908
C	0.328188	12.806250	4.055381
C	-0.300583	14.047716	3.955889
H	-0.564992	14.434651	2.973882
C	-0.609773	14.790654	5.087565
H	-1.097755	15.757791	4.990949
C	-0.304237	14.284433	6.342881
H	-0.567829	14.857731	7.229110
C	0.322563	13.045564	6.494453
C	0.562037	11.988829	2.791697
H	1.191837	11.129522	3.046760
C	-0.773186	11.439801	2.268782
H	-1.283027	10.840247	3.032511
H	-0.616095	10.809801	1.384460
H	-1.447075	12.258859	1.987619
C	1.286628	12.782846	1.698690
H	0.674910	13.614363	1.329636
H	1.509132	12.135047	0.842976
H	2.231668	13.202590	2.062738
C	0.615392	12.530421	7.896711
H	0.939310	11.487473	7.811100
C	1.761529	13.317157	8.546294
H	2.682196	13.253106	7.954397
H	1.976601	12.927841	9.548431
H	1.501023	14.378197	8.645691
C	-0.634499	12.555814	8.786966
H	-0.959259	13.580906	9.004286
H	-0.425375	12.063594	9.745024
H	-1.472161	12.032048	8.312047
N	1.239246	8.030826	5.459901
C	2.533691	8.245148	5.273540
C	3.443986	7.061567	5.029038
H	3.275396	6.659073	4.023157
H	4.493944	7.349797	5.111577
H	3.235804	6.252308	5.735344
C	0.660667	6.725076	5.335922
C	0.328209	6.237142	4.055395
C	-0.300549	4.995669	3.955908
H	-0.564952	4.608727	2.973901
C	-0.609733	4.252733	5.087587
H	-1.097705	3.285591	4.990974
C	-0.304204	4.758963	6.342901
H	-0.567792	4.185666	7.229132
C	0.322583	5.997838	6.494469
C	0.562056	7.054560	2.791709
H	1.191849	7.913871	3.046771
C	-0.773169	7.603577	2.268789
H	-1.283017	8.203129	3.032515
H	-0.616081	8.233575	1.384465
H	-1.447052	6.784513	1.987627
C	1.286655	6.260543	1.698707
H	0.674944	5.429021	1.329654
H	1.509157	6.908341	0.842991
H	2.231697	5.840807	2.062759
C	0.615403	6.512989	7.896726
H	0.939316	7.555939	7.811111
C	1.761541	5.726262	8.546319
H	2.682211	5.790316	7.954426
H	1.976605	6.115583	9.548455
H	1.501040	4.665221	8.645718
C	-0.634493	6.487592	8.786975
H	-0.959249	5.462498	9.004294
H	-0.425376	6.979814	9.745033
H	-1.472155	7.011353	8.312049
Zn	-4.589612	9.521702	7.577195
H	-3.731411	9.521707	5.907558
H	-2.865275	9.521698	8.263107
N	-5.789119	11.012577	7.952060
C	-7.083564	10.798257	8.138420
C	-7.684372	9.521700	8.159829
H	-8.760176	9.521699	8.294793
C	-7.993857	11.981839	8.382926
H	-7.825269	12.384327	9.388810
H	-9.043815	11.693611	8.300384
H	-7.785672	12.791101	7.676625
C	-5.210537	12.318325	8.076044
C	-4.878076	12.806254	9.356572
C	-4.249313	14.047724	9.456063
H	-3.984907	14.434662	10.438070
C	-3.940127	14.790663	8.324386

H	-3.452152	15.757803	8.421002
C	-4.245660	14.284439	7.069070
H	-3.982071	14.857738	6.182841
C	-4.872450	13.045566	6.917499
C	-5.111921	11.988833	10.620257
H	-5.741721	11.129526	10.365194
C	-3.776697	11.439807	11.143169
H	-3.266853	10.840259	10.379437
H	-3.933786	10.809801	12.027488
H	-3.102811	12.258865	11.424336
C	-5.836511	12.782850	11.713265
H	-5.224793	13.614365	12.082320
H	-6.059017	12.135049	12.568977
H	-6.781550	13.202596	11.349217
C	-5.165276	12.530421	5.515241
H	-5.489189	11.487471	5.600852
C	-6.311417	13.317151	4.865657
H	-7.232085	13.253094	5.457553
H	-6.526485	12.927834	3.863519
H	-6.050916	14.378192	4.766261
C	-3.915384	12.555821	4.624987
H	-3.590632	13.580915	4.407666
H	-4.124504	12.063597	3.666930
H	-3.077718	12.032063	5.099909
N	-5.789118	8.030824	7.952054
C	-7.083563	8.245142	8.138415
C	-7.993856	7.061560	8.382917
H	-7.825267	6.659068	9.388799
H	-9.043814	7.349787	8.300376
H	-7.785671	6.252300	7.676612
C	-5.210536	6.725076	8.076033
C	-4.878073	6.237143	9.356559
C	-4.249310	4.995673	9.456045
H	-3.984903	4.608732	10.438051
C	-3.940124	4.252738	8.324366
H	-3.452148	3.285598	8.420977
C	-4.245658	4.758967	7.069052
H	-3.982069	4.185670	6.182820
C	-4.872450	5.997839	6.917485
C	-5.111919	7.054560	10.620246
H	-5.741718	7.913868	10.365187
C	-3.776695	7.603582	11.143162
H	-3.266849	8.203131	10.379433
H	-3.933784	8.233585	12.027483
H	-3.102811	6.784522	11.424328
C	-5.836511	6.260540	11.713250
H	-5.224794	5.429022	12.082302
H	-6.059017	6.908336	12.568965
H	-6.781551	5.840796	11.349200
C	-5.165277	6.512989	5.515229
H	-5.489189	7.555939	5.600844
C	-6.311419	5.726262	4.865643
H	-7.232086	5.790319	5.457540
H	-6.526487	6.115582	3.863507
H	-6.050920	4.665221	4.766245
C	-3.915386	6.487591	4.624974
H	-3.590634	5.462497	4.407650
H	-4.124506	6.979817	3.666918
H	-3.077719	7.011348	5.099897

TS-1.log

SCF (wB97x) = -2610.22499084
E(SCF)+ZPE(0 K)= -2608.900389
H(298 K)= -2608.824098
G(298 K)= -2609.015342
Lowest Frequency = -755.4758cm-1

H	-2.423343	9.469339	6.245228
N	0.961057	10.826180	5.699386
C	2.206715	10.604987	5.281746
C	2.698951	9.362616	4.843581
H	3.745762	9.355114	4.560953
C	3.210954	11.740448	5.309692
H	2.777895	12.655041	4.892937
H	4.107839	11.480454	4.743879
H	3.509031	11.970375	6.339149
C	0.665689	12.071513	6.346140
C	-0.048775	13.074052	5.665070
C	-0.342083	14.262754	6.341163
H	-0.895823	15.044248	5.824692
C	0.066456	14.466473	7.649359
H	-0.158579	15.403232	8.154197
C	0.761000	13.462792	8.316935
H	1.066211	13.624573	9.347821
C	1.060745	12.254406	7.691887
C	-0.485316	12.925989	4.214208
H	-0.217170	11.917356	3.879193
C	-2.002372	13.083803	4.058535
H	-2.547867	12.387462	4.707909
H	-2.304119	12.881210	3.023596
H	-2.331099	14.098145	4.314329
C	0.260648	13.913793	3.307540
H	0.034052	14.950938	3.583133
H	-0.035368	13.772122	2.261569
H	1.346777	13.780089	3.373416
C	1.757665	11.141690	8.468120
H	2.285067	10.499974	7.754778
C	2.800022	11.654317	9.466850
H	3.514056	12.336044	8.990397
H	3.361522	10.812325	9.887044
H	2.337460	12.185262	10.307031
C	0.723727	10.260357	9.181288
H	0.164706	10.840645	9.925720
H	1.214774	9.424283	9.695303
H	-0.004527	9.840807	8.475499
N	0.778358	7.908041	5.065775
C	2.070546	8.096864	4.849993
C	2.982720	6.905306	4.646861
H	2.524521	6.139321	4.017429

H	3.936267	7.210023	4.210409
H	3.187410	6.438710	5.618648
C	0.250533	6.574686	5.111993
C	-0.274456	5.983447	3.943926
C	-0.808851	4.696879	4.034748
H	-1.204597	4.221793	3.139347
C	-0.856891	4.016660	5.244140
H	-1.282376	3.016987	5.292965
C	-0.365608	4.624002	6.391446
H	-0.421822	4.097674	7.342335
C	0.198028	5.900009	6.347770
C	-0.269408	6.691528	2.594440
H	0.046751	7.727896	2.759937
C	-1.668319	6.728241	1.965994
H	-2.403366	7.167704	2.648129
H	-1.655001	7.331649	1.050312
H	-2.012670	5.722653	1.694505
C	0.728333	6.048892	1.620911
H	0.470881	4.999095	1.433629
H	0.715122	6.574066	0.658684
H	1.753980	6.078800	2.004310
C	0.706394	6.534584	7.635370
H	1.189341	7.485615	7.380876
C	1.753371	5.662210	8.338256
H	2.593655	5.426964	7.674946
H	2.150079	6.178975	9.220152
H	1.322381	4.712856	8.677650
C	-0.462379	6.840212	8.579171
H	-0.979997	5.921753	8.877676
H	-0.108568	7.343904	9.487403
H	-1.211987	7.484010	8.100132
H	-4.658410	9.385229	4.649701
N	-5.748183	11.125205	7.240878
C	-6.886900	10.939492	7.888501
C	-7.383007	9.674486	8.278314
H	-8.371803	9.684046	8.722942
C	-7.724680	12.136513	8.285774
H	-7.193139	12.727531	9.040753
H	-8.684969	11.826975	8.702674
H	-7.900818	12.799490	7.434317
C	-5.288802	12.456482	6.971429
C	-4.421679	13.077731	7.893058
C	-3.935251	14.353540	7.603725
H	-3.258160	14.837872	8.304938
C	-4.296443	15.011805	6.436356
H	-3.917134	16.010132	6.230100
C	-5.138642	14.382992	5.529415
H	-5.409488	14.895559	4.608293
C	-5.639191	13.101384	5.766715
C	-3.984417	12.390914	9.179965
H	-4.494059	11.422051	9.240075
C	-2.474692	12.127337	9.165503
H	-2.179839	11.525618	8.295561
H	-2.166226	11.592676	10.072246
H	-1.907140	13.062991	9.114182
C	-4.378131	13.196421	10.424230
H	-3.868685	14.167221	10.449646
H	-4.101263	12.651954	11.334992
H	-5.457105	13.386141	10.457967
C	-6.532533	12.453793	4.715890
H	-6.803930	11.452947	5.070364
C	-7.829921	13.245611	4.501768
H	-8.401418	13.360630	5.429550
H	-8.470500	12.735240	3.773275
H	-7.620972	14.250164	4.114162
C	-5.790592	12.287113	3.382938
H	-5.508134	13.260828	2.962791
H	-6.432375	11.779918	2.652408
H	-4.883408	11.686472	3.505210
N	-5.505339	8.203621	7.825975
C	-6.724244	8.431336	8.310840
C	-7.472248	7.305615	8.997727
H	-7.045680	7.104487	9.987292
H	-8.526146	7.560946	9.126018
H	-7.395922	6.377308	8.423012
C	-4.853275	6.970649	8.153487
C	-4.291569	6.809838	9.441085
C	-3.641857	5.614577	9.742711
H	-3.210715	5.473737	10.731276
C	-3.521924	4.600703	8.798457
H	-3.010902	3.674625	9.052289
C	-4.052797	4.781035	7.531047
H	-3.949231	3.991572	6.789227
C	-4.728218	5.955783	7.186407
C	-4.337780	7.929933	10.474538
H	-5.157424	8.607535	10.215283
C	-3.044544	8.753683	10.421951
H	-2.871058	9.161892	9.418170
H	-3.090954	9.594316	11.126134
H	-2.177008	8.134908	10.683969
C	-4.598104	7.430090	11.899447
H	-3.752858	6.853673	12.293280
H	-4.751835	8.280474	12.573412
H	-5.488830	6.792788	11.946899
C	-5.321843	6.080467	5.790541
H	-5.768108	7.076636	5.695438
C	-6.446783	5.060476	5.571991
H	-7.243218	5.175150	6.316681
H	-6.890980	5.187949	4.577969
H	-6.069189	4.033017	5.641998
C	-4.245977	5.948874	4.706905
H	-3.796855	4.948078	4.702652
H	-4.677509	6.133908	3.715766
H	-3.436814	6.674001	4.859796
Pd	-2.938648	9.525302	4.587585
Mg	-4.668156	9.543350	6.535925
Mg	-0.508046	9.463254	5.351503
H	-2.842026	9.642348	2.984208
H	-1.795235	9.683896	3.435839

TS-2.log

SCF (wB97x) = -2610.22487096
E(SCF)+ZPE(0 K)= -2608.896873
H(298 K)= -2608.821325
G(298 K)= -2609.009705
Lowest Frequency = -90.3402cm-1

H	-1.739474	8.943482	6.568660
N	1.255356	10.927019	6.032222
C	2.523724	10.679381	5.700547
C	2.982754	9.503078	5.086623
H	4.048163	9.468725	4.886383
C	3.579763	11.716194	6.027237
H	3.282331	12.704676	5.662577
H	4.542481	11.450320	5.586553
H	3.708435	11.805433	7.112358
C	0.941092	12.112101	6.772757
C	0.414055	13.236992	6.109571
C	0.064614	14.358126	6.868832
H	-0.335365	15.236633	6.365640
C	0.223905	14.373059	8.246880
H	-0.049089	15.255619	8.820752
C	0.731854	13.250358	8.891946
H	0.843665	13.264366	9.973582
C	1.094443	12.109715	8.177609
C	0.228576	13.275304	4.598651
H	0.520246	12.297208	4.199042
C	-1.235447	13.521690	4.214383
H	-1.905415	12.777639	4.659887
H	-1.357132	13.473656	3.125577
H	-1.571872	14.512591	4.544086
C	1.142643	14.323501	3.950669
H	0.896726	15.333507	4.300823
H	1.027265	14.310165	2.860676
H	2.197943	14.138125	4.181859
C	1.599577	10.872921	8.911776
H	2.148453	10.252525	8.195517
C	2.561280	11.202004	10.058101
H	3.385730	11.840994	9.721126
H	2.989898	10.279227	10.465095
H	2.054861	11.715676	10.883683
C	0.419268	10.035441	9.422464
H	-0.164121	10.597985	10.162858
H	0.771637	9.110921	9.897569
H	-0.263599	9.757230	8.609565
N	0.963264	8.202685	4.874451
C	2.268421	8.329937	4.740498
C	3.088966	7.197916	4.158813
H	3.053869	7.246515	3.063630
H	4.134697	7.273450	4.465838
H	2.695535	6.221504	4.453249
C	0.282189	6.980754	4.566151
C	-0.111668	6.693739	3.244778
C	-0.870954	5.544736	3.011315
H	-1.185214	5.312185	1.995768
C	-1.243340	4.702913	4.049244
H	-1.838338	3.815485	3.847194
C	-0.861552	5.005910	5.350716
H	-1.170389	4.351351	6.162128
C	-0.104915	6.142493	5.635024
C	0.225617	7.607533	2.074363
H	0.873161	8.410744	2.444044
C	-1.041339	8.262018	1.505901
H	-1.591659	8.810146	2.278676
H	-0.783894	8.961498	0.700931
H	-1.718151	7.505475	1.089716
C	0.985961	6.864366	0.968082
H	0.357625	6.096585	0.501081
H	1.292835	7.562162	0.180245
H	1.882852	6.365955	1.353439
C	0.307135	6.445867	7.069812
H	0.510788	7.521678	7.138354
C	1.611931	5.721891	7.428719
H	2.434035	6.021194	6.768703
H	1.908155	5.948715	8.460200
H	1.487650	4.635215	7.341941
C	-0.787822	6.125756	8.091239
H	-0.950087	5.046527	8.199212
H	-0.499610	6.508531	9.078447
H	-1.743411	6.584688	7.812570
H	-3.858178	8.055701	5.746040
N	-5.823193	10.916803	7.146647
C	-6.860486	10.966220	7.973807
C	-7.319473	9.879354	8.747521
H	-8.213379	10.074116	9.329529
C	-7.601666	12.274284	8.165720
H	-7.009445	12.940992	8.805026
H	-8.566813	12.109804	8.649564
H	-7.757182	12.798234	7.219495
C	-5.423914	12.100170	6.440104
C	-4.581259	13.036393	7.073663
C	-4.195099	14.170892	6.360833
H	-3.543916	14.904445	6.829974
C	-4.620339	14.375148	5.054047
H	-4.315056	15.268634	4.514571
C	-5.414946	13.424241	4.429841
H	-5.718905	13.580504	3.398270
C	-5.819919	12.266835	5.098811
C	-4.043672	12.786615	8.477296
H	-4.765637	12.160322	9.014353
C	-2.722101	12.009057	8.403122
H	-2.832305	11.057967	7.865556
H	-2.339843	11.785815	9.407204
H	-1.960538	12.591153	7.870016
C	-3.851828	14.066734	9.296356
H	-3.019301	14.669755	8.914437
H	-3.614626	13.813226	10.335761
H	-4.754216	14.689045	9.292977
C	-6.677850	11.228969	4.384587

H	-6.489272	10.259355	4.862801
C	-8.175178	11.527457	4.540254
H	-8.491420	11.493492	5.588194
H	-8.771737	10.791703	3.987971
H	-8.413620	12.522593	4.144289
C	-6.316749	11.072929	2.904308
H	-6.615326	11.949256	2.316215
H	-6.842185	10.207320	2.484945
H	-5.241077	10.913627	2.774402
N	-5.635683	8.184859	8.344962
C	-6.741138	8.610759	8.946967
C	-7.431006	7.716266	9.956374
H	-6.862146	7.690910	10.893373
H	-8.436455	8.079937	10.177877
H	-7.493350	6.685792	9.594485
C	-5.048652	6.934513	8.729070
C	-4.263497	6.867659	9.900370
C	-3.658350	5.656107	10.232054
H	-3.047808	5.590682	11.129919
C	-3.812833	4.531472	9.431151
H	-3.338385	3.593339	9.708717
C	-4.566614	4.616401	8.269875
H	-4.678045	3.736614	7.639895
C	-5.187109	5.809794	7.891795
C	-4.010579	8.096216	10.765818
H	-4.753459	8.858564	10.509002
C	-2.629843	8.686912	10.451635
H	-2.524780	8.919958	9.384678
H	-2.461125	9.609932	11.020623
H	-1.833589	7.977140	10.709879
C	-4.147322	7.812593	12.265750
H	-3.359991	7.140997	12.627152
H	-4.065946	8.745403	12.835309
H	-5.112954	7.351293	12.503527
C	-6.010832	5.844917	6.611455
H	-6.235352	6.893453	6.382322
C	-7.355122	5.130168	6.803401
H	-7.943797	5.588690	7.606316
H	-7.949034	5.171542	5.882800
H	-7.202043	4.074678	7.060021
C	-5.252252	5.262662	5.412452
H	-5.096176	4.182407	5.521139
H	-5.826955	5.419845	4.492149
H	-4.273164	5.738327	5.286475
Pd	-2.776335	9.164089	5.215173
Mg	-4.728858	9.241959	6.879720
Mg	-0.265950	9.687815	5.528233
H	-3.800832	9.435569	4.043714
H	-1.799744	10.230457	4.392134

5. References

[1] Feldman, J.; McLain, S. J.; Parthasarathy, A.; Marshall, W. J.; Calabrese, J. C.; Arthur, S. D. Electrophilic Metal Precursors and a β-Diimine Ligand for Nickel(II)- and Palladium(II)-Catalyzed Ethylene Polymerization. *Organometallics* **1997**, *16*, 1514–1516.

[2] Garçon, M.; Bakewell, C.; Sackman, G. A.; White, A. J. P.; Cooper, R. I.; Edwards, A. J.; Crimmin, M. R. A hexagonal planar transition-metal complex. *Nature* **2019**, *574*, 390–393.

[3] Garçon, M.; Phanopoulos, A.; Sackman, G. A.; Richardson, C.; White, A. J. P.; Cooper, R. I.; Edwards, A. J.; Crimmin, M. R. The Continuum Between Hexagonal Planar and Trigonal Planar Geometries. *Angew. Chem. Int. Ed..* **2022**, *61*, e202211948.

[4] Garçon, M.; Mun, N. W.; White, A. J. P.; Crimmin, M. R. Palladium-Catalysed C–H Bond Zincation of Arenes: Scope, Mechanism, and the Role of Heterometallic Intermediates. *Angew. Chem. Int. Ed.* **2021**, *60*, 6145–6153.

[5] Clark, H. C.; Manzer, L. E. Reactions of (π-1,5-Cyclooctadiene) Organoplatinum(II) Compounds and the Synthesis of Perfluoroalkylplatinum Complexes. *J. Organomet. Chem.* **1973**, *59*, 411–428.

[6] a) SHELXTL v5.1, Bruker AXS, Madison, WI, 1998. b) SHELX-2013, G.M. Sheldrick, *Acta Cryst.*, **2015**, *C71*, 3–8.

[7] Cammarota, R. C.; Xie, J. Burgess, S. A.; Vollmer, M. V.; Vogiatzis, K. D.; Ye, J.; Linehan, J. C.; Appel, A. M.; Hoffmann, C.; Wang, X.; Young, Jr., V. G.; Lu, C. C. Thermodynamic and Kinetic Studies of H_2 and N_2 Binding to Bimetallic Nickel-Group 13 Complexes and Neutron Structure of a Ni(η^2-H_2) Adduct. *Chem. Sci.* **2019**, *10*, 7029–7042

[8] Brunner, E. Solubility of Hydrogen in 10 Organic Solvents at 298.15, 323.15, and 373.15 K. *J. Chem. Eng. Data* **1985**, *30*, 269–273.

[9] Spek, A. L. PLATON, A Multipurpose Crystallographic Tool, Utrecht University, Utrecht, The Netherlands (2003, 2009). See also Spek, A. L. PLATON SQUEEZE: A Tool for the Calculation of the Disordered Solvent Contribution to the Calculated Structure Factors. *Acta Cryst. C Struct. Chem.* **2015**, *C71*, 9–18.

[10] Frisch, M. J.; Trucks, G. W.; Schlegel, H. B.; Scuseria, G. E.; Robb, M. A.; Cheeseman, J. R.; Scalmani, G.; Barone, V.; Mennucci, G.; Petersson, G. A.; Nakatsuji, H.; Caricato, M.; Li, X.; Hratchian, H. P.; Izmaylov, A. F.; Bloino, J.; Zheng, G.; Sonnenberg, J. L.; Hada, M.; Ehara, M.; Toyota, K.; Fukuda, R.; Hasegawa, J.; Ishida, M.; Nakajima, T.; Honda, Y.; Kitao, O.; Nakai, H.; Vreven, T.; Montgomery, J. A., Jr.; Peralta, J. E.; Ogliaro, F.; Bearpark, M.; Heyd, J. J.; Brothers, E.; Kudin, K. N.; Staroverov, V. N.; Kobayashi, R.; Normand, J.; Raghavachari, K.; Rendell, A.; Burant, J. C.; Iyengar, S. S.; Romasi, J.; Cossi, M.; Rega, N.; Millam, J. M.; Klene, M.; Knox, J. E.; Cross, J. B.; Bakken, V.; Adamo, C.; Jaramillo, J.; Gomperts, R.; Stratmann, R. E.; Yazyev, O.; Austin, A. J.; Cammi, R.; Pomelli, C.; Ochterski, J. W.; Martin, R. L.; Morokuma, K.; Zakrzewski, V. G.; Voth, G. A.; Salvador, P.; Dannenberg, J. J.; Dapprich, S.; Daniels, A. D.; Farkas, Ö.; Foresman, J. B.; Ortiz, J. V.; Cioslowski, J.; Fox, D. J. *Gaussian 09, Revision D.01*; Gaussian, Inc., Wallingford, CT, USA (2009).

[11] Chai, J.-D.; Head-Gordon, M. Systematic Optimization of Long-Range Corrected Hybrid Density Functionals. *J. Chem. Phys.* **2008**, *128*, 084106.

[12] Garçon, M.; White, A. J. P.; Crimmin, M. R. Palladium-Catalysed Magnesiation of Benzene. *Chem. Commun.* **2018**, *54*, 12326–12328.

[13] *NBO 6.0.* Glendening, E. D.; Badenhoop, J. K.; Reed, A. E.; Carpenter, J. E.; Bohmann, J. A.; Morales, C. M.; Landis, C. R.; Weinhold, F. Theoretical Chemistry Institute, University of Misconsin, Madison (2013).

[14] AIMAll (Version 13.10.19), Keith, T. A. TK Gristmill Software, Overland Park, KS, USA (2013) (aim.tkgristmill.com).

[15] Johnson, E. R.; Keinan, S.; Mori-Sánchez, P.; Contreras-García, J.; Cohen, A. J.; Yang, W. Revealing Noncovalent Interactions. *J. Am. Chem. Soc.* **2010**, *132*, 6498–6506.
